# Achieving Extreme Solubility and Green Solvent-Processed Organic Field-Effect Transistors: A Viable Asymmetric Functionalization of [1]Benzothieno[3,2‑*b*][1]benzothiophenes

**DOI:** 10.1021/acsami.5c12618

**Published:** 2025-08-22

**Authors:** Tevhide Ayça Yıldız, İbrahim Deneme, Hakan Usta

**Affiliations:** Department of Materials Science and Nanotechnology Engineering, 346448 Abdullah Gül University, Kayseri 38080, Turkey

**Keywords:** asymmetric functionalization, p-type semiconductor, organic field-effect transistor, green-solvent processing, Hansen solubility parameters

## Abstract

Novel structural engineering strategies for solubilizing high-mobility semiconductors are critical, which enables green solvent processing for eco-friendly, sustainable device fabrication, and unique molecular properties. Here, we introduce a viable asymmetric functionalization approach, synthesizing monocarbonyl [1]­benzothieno­[3,2-*b*]­[1]­benzothiophene molecules on a gram scale in two transition-metal-free steps. An unprecedented solubility of up to 176.0 mg·mL^–1^ (at room temperature) is achieved, which is the highest reported to date for a high-performance organic semiconductor. The single-crystal structural analysis reveals a herringbone motif with multiple edge-to-face interactions and nonclassical hydrogen bonds involving the carbonyl unit. The asymmetric backbones adopt an antiparallel arrangement, enabling face-to-face π-π interactions. The mono­(alkyl-aryl)­carbonyl-BTBT compound, ***m*-C_6_PhCO-BTBT** enables formulations in varied green solvents, including acetone and ethanol, all achieving *p*-channel top-contact/bottom-gate OFETs in ambient conditions. Charge carrier mobilities of up to 1.87 cm^2^/V·s (μ_eff_ ≈ 0.4 cm^2^/V·s; I_on_/I_off_ ≈ 10^7^–10^8^) were achieved. To the best of our knowledge, this is one of the highest OFET performances achieved using a green solvent. Hansen solubility parameters (HSP) analysis, combined with Scatchard–Hildebrand regular solution theory and single-crystal packing analysis, elucidates this exceptional solubility and reveals unique relationships between molecular structure, interaction energy densities, cohesive energetics, and solute–solvent distances (*R*_a_). An optimal solute–green solvent interaction distance in HSP space proves critical for green solvent-processed thin-film properties. This asymmetric functionalization approach, with demonstrated unique solubility insights, provides a foundation for designing green solvent-processable π-conjugated systems, potentially advancing innovation in sustainable (opto)­electronics and bioelectronics.

## Graphical abstract

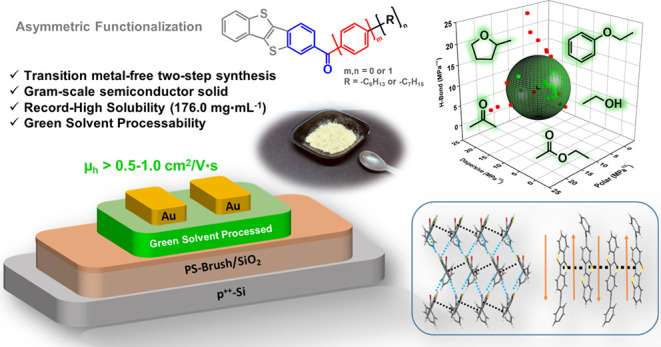

## Introduction

With the rising demand for eco-friendly device fabrication, the rational design and development of high-solubility, high-mobility molecular π-architectures—capable of being solution-processed into semiconducting thin films using green solvents—are of great interest for use in organic (opto)­electronics.^1,2^ To date, novel structural engineering approaches for realizing solubility in green solvents remain scarce, and most of the recent green solvent processing efforts have focused on already existing high-mobility semiconductors (Table S1). In addition, some portion of these green solvent studies employ only nonchlorinated solvents,^2,3^ without considering multiple dimensions of solvent greenness, such as health, safety, environmental footprint, and sustainability.^4^ Novel molecular engineering strategies to solubilize high-mobility (μ > 0.5–1.0 cm^2^/V·s) semiconductors in truly green solvents remain highly desirable.^1,5^ Furthermore, a robust understanding of the factors governing solubility in these solvents, supported by well-established quantitative thermodynamic correlations among molecular structures, physicochemical properties, and solubility, as well as their influence on the properties of semiconducting thin films, is critical to advancing the field. As the field of soluble organic semiconductors continues to expand, small molecules based on coplanar diacene-fused thienothiophene (DAcTT) π-scaffolds have emerged as one of the most appealing organic semiconductor family in the past two decades.^6^ Soluble [1]­benzothieno­[3,2-*b*]­[1]­benzothiophene (BTBT) π-system in this family enables large frontier orbital coefficients on the sulfur atoms and forms a favorable solid-state electronic structure for efficient hole transport.^7,8^ As a direct result of their phene-like π-electronic structure, BTBTs have large band gaps (>3.5 eV) and deep HOMO levels (<−5.5 eV). Today, BTBT remains an unprecedented π-framework enabling facile synthetic modification and good solubility in common organic solvents and showing optical transparency and ambient-stable high hole mobility. In addition, from a materials production standpoint, the synthesis of BTBTs could typically be performed in a small number of steps with convenient chromatographic purifications, which makes these semiconductors quite attractive for industrial-scale applications. Over the past decade, a large variety of π-electron-rich BTBT semiconductors (Table S2) have been developed including mono- and dialkyl-substituted BTBTs (e.g., **(mono)-C**_**13**_ **-BTBT** ^9^ and **C**_**8**_ **-BTBT** ^10^), diaryl-substituted BTBTs (e.g., **DPh-BTBT** ^7^), and monoaryl/monoalkyl-substituted BTBTs (e.g., **C**_**n**_ **-BTBT-Ph** ^11^ and **BTBT-Ph–C**_**6/12**_ ^12^) for use in *p*-channel organic field-effect transistors (OFETs). In these semiconductors, although the BTBT π-core is the active charge-transporting moiety, an alkyl and/or aryl substitution has been employed to realize proper semiconductor thin-film formation with efficient hole transport and solid-state characteristics.^12^ Among these BTBTs, some structures have been reported to have good solubility in common organic solvents, and their synthetic purification and thin-film fabrication were carried out using solution-based techniques (Table S2). Typically, medium-length linear alkyl chain (-*n*-C_n_H_2n+1_ (*n* = 3–8)) substitution has been employed to realize proper solution-processability, and the highest reported solubility was for dialkyl-substituted BTBTs (**C**_**8**_ **-BTBT**s^10^) with values up to 90 mg·mL^–1^, followed by solubilities of up to 9–11 mg·mL^–1^ for **C**_**n**_ **-BTBT-Ph**s.^11^

With the goal of expanding further the versatility of the BTBT π-scaffold, we have recently employed a different molecular engineering approach beyond the previously used alkyl/aryl substitutions and explored a difunctionalization motif with electron-withdrawing units.^13^ This approach allowed us to demonstrate the first examples of both functionalized and electron-transporting (*n*-type) BTBTs. In these studies, we demonstrated that carbonyls and dicyanovinylenes in difunctionalized BTBT π-frameworks are very effective electron-withdrawing functional groups that extend the π-conjugation and considerably stabilize frontier orbital energies (−ΔE_HOMO_ ≈ 0.7–0.9 eV and −ΔE_LUMO_ ≈ 1.4–1.6 eV). To the best of our knowledge, these are the only known examples of carbonyl-functionalized BTBT semiconductors reported to date. It is important to note that during the development of novel semiconductor structures in the past few decades, carbonyls have typically been used either to realize electron transport or to reduce the HOMO level of π-electron-rich oligothiophenes for ambient-stable hole transport.^14,15^ Therefore, as far as hole transport is concerned, carbonyl functionalization has never been considered as a potential strategy for BTBTs since they already have highly stabilized HOMOs (−5.5 to −5.8 eV, Table S2).

A novel monocarbonyl functionalization approach on the fused BTBT π-system could generate an asymmetric molecular structure, enabling unique solubility in green solvents through polar and hydrogen-bonding interactions while introducing new structural and electronic properties. Given that green solvents typically exhibit high polar and hydrogen-bonding solubility parameters (δ_P_ and δ_H_), this functionalization could facilitate eco-friendly processing and enhance the versatility of DAcTTs and other intrinsically insoluble organic semiconductors in sustainable (optoe)­electronics.^16,17^ However, a critical question remains as to whether monocarbonyl functionalization delivers high-performance BTBT-based *p*-channel OFETs. Equally important is a thorough understanding of the relationships among structure, electronic properties, intermolecular interactions, physicochemical properties, and solubility.

We herein present a unique molecular engineering on the BTBT π-system by employing mono­(aryl)­carbonyl functionalization with one hexyl (*n*-C_6_H_13_) substituent, and demonstrate the design, synthesis, and characterization of a new high-mobility asymmetric BTBT semiconductor, ***m*** **-C**_**6**_ **PhCO-BTBT** (Figure 1). Specifically, introducing a phenyl group not only extends the π-system of the new molecular structure but also, being unfused to the main BTBT π-core, retains conformational flexibility, enabling optimized intermolecular interactions in both the solid state and solution. The new molecule was produced on a gram scale through a two-step transition-metal-free synthesis, and detailed structural, physicochemical, and (opto)­electronic characterizations were performed. Mono­(aryl)­carbonyl functionalization stabilizes frontier molecular orbitals (ΔE_HOMO_ = −0.29 eV vs ΔE_LUMO_ = −0.65 eV) and induces an asymmetric π-electronic structure having a ground-state molecular dipole moment (μ_g_) of 3.17 D and a large excited-state dipole moment (μ_e_) of 12.69 D. Moreover, the asymmetric π-backbone demonstrates a significant degree of polarizability. The single-crystal structural analysis reveals a herringbone motif with multiple edge-to-face interactions and nonclassical hydrogen bonds involving the carbonyl unit. The antiparallel arrangement of the asymmetric backbones also facilitates face-to-face π-π interactions. The new π-electronic structure yields excellent solubility behavior in varied organic solvents, including a number of eco-friendly green solvents. Hansen solubility parameters (HSP) analysis, combined with thermodynamics data from differential scanning calorimetry (DSC), was employed to explore the solubility behavior of the new molecule and to elucidate unique structure-solubility-cohesive energetics relationships. In this analysis, two other novel molecules, ***m*** **-PhCO-BTBT** and ***m*** **-C**_**7**_ **CO-BTBT**, were also synthesized and used as the reference molecules, along with some structurally related BTBT semiconductors from the earlier studies in the literature. The HSPs for ***m*** **-C**_**6**_ **PhCO-BTBT** are determined to be δ_D_ = 18.9 MPa^1/2^, δ_P_ = 5.7 MPa^1/2^, and δ_H_ = 5.8 MPa^1/2^ with an interaction radius (*R*_0_) of 8.0 MPa^1/2^. Strong thermodynamic correlations of the molecular solubility with thermal properties are established for the first time in the literature. ***m*** **-C**_**6**_ **PhCO-BTBT** exhibits prompt dissolution at room temperature in chloroform, displaying a remarkable room temperature solubility of 176.0 mg·mL^–1^ (0.41 M), which, to the best of our knowledge, represents the highest solubility ever reported for a high-performance organic semiconductor. This impressive solubility allowed for the preparation of semiconductor solutions (solubility up to ∼12.5 mg·mL^–1^ at room temperature) in eco-friendly green solvents such as 2-methyltetrahydrofuran, ethyl acetate, ethoxybenzene, acetone, and ethanol, suitable for thin-film solution processing. The charge-transport characteristics of the new molecule were studied in solution-processed top-contact/bottom-gate (TC/BG) OFETs. In the field of π-conjugated small molecules, ***m*** **-C**_**6**_ **PhCO-BTBT** is now a rare example of a high-mobility *p*-type semiconductor with a deep HOMO level of −6.04 eV. The maximum saturation hole mobilities were achieved for thin films spin-coated from 2-methyltetrahydrofuran (μ_h_^max^ = 1.87 cm^2^/V·s, μ_h_^avg^ = 1.21 cm^2^/V·s (I_on_/I_off_ = 10^7^-10^8^) and ethyl acetate (μ_h_^max^ = 0.62 cm^2^/V·s, μ_h_^avg^ = 0.41 cm^2^/V·s (I_on_/I_off_ = 10^6^-10^7^) solutions. Relatively lower μ_h_s of 0.07–0.11 cm^2^/V·s (I_on_/I_off_ = 10^5^-10^6^ were realized for spin-coated thin films from acetone and ethoxybenzene, while drop-casted thin films from ethanol yielded μ_h_s of ∼0.001 cm^2^/V·s (I_on_/I_off_ ≈ 10^4^). Microstructural and morphological characterizations shed light on the observed transistor behaviors, revealing the key effects of differences in the solute–green solvent interactions.

**Figure 1:**
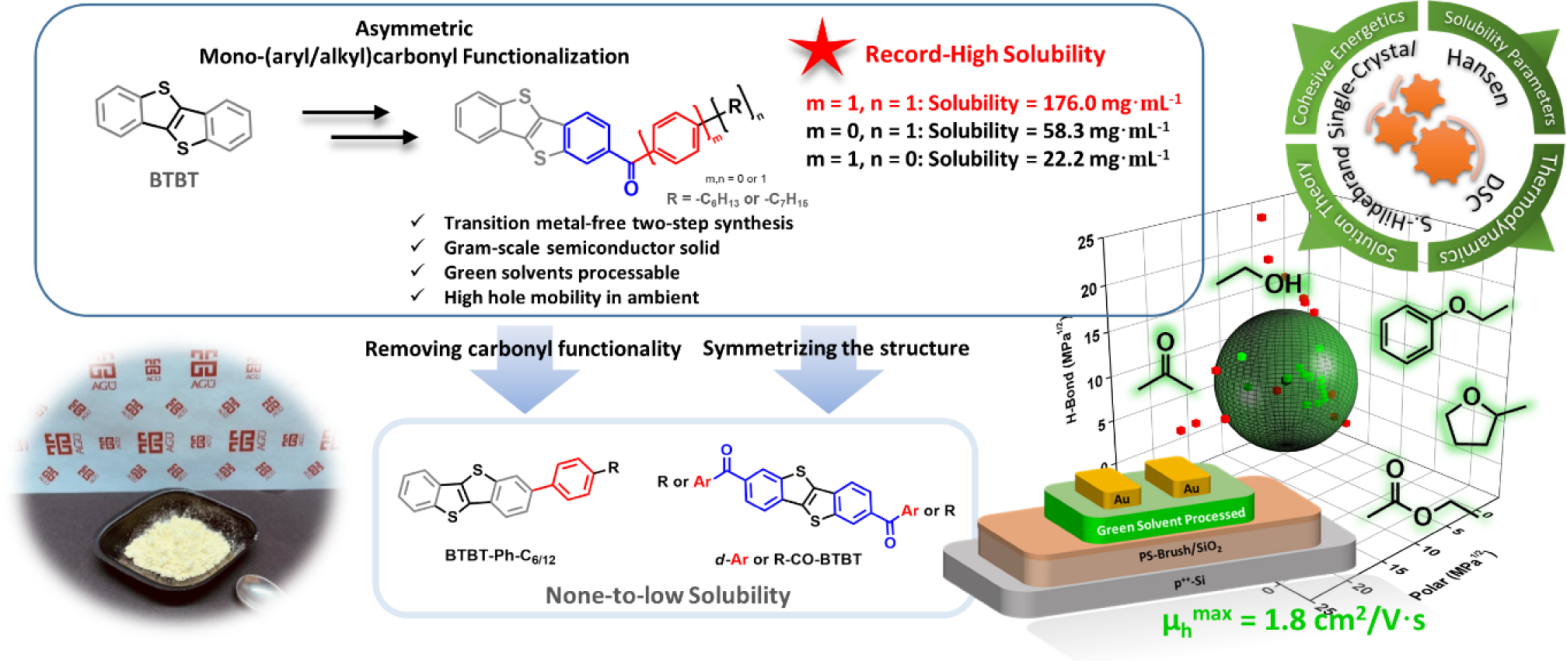
Asymmetric mono­(aryl/alkyl)­carbonyl functionalization approach for synthesizing our new molecules: ***m*** **-C**_**6**_ **PhCO-BTBT** (*m* = 1, *n* = 1), ***m*** **-PhCO-BTBT** (*m* = 1, *n* = 0), and ***m*** **-C**_**7**_ **CO-BTBT** (*m* = 0, *n* = 1). The structural design and key advantages are illustrated. Insets display a photograph of the gram-scale ***m*** **-C**_**6**_ **PhCO-BTBT** solid, schemes of the Hansen sphere, green solvents used in this study, and the fabricated OFET structure.

## Results and Discussion

### Synthesis, Characterizations, and Molecular Properties

The syntheses of the new monocarbonyl molecules, ***m*** **-C**_**6**_ **PhCO-BTBT**, *m* **-PhCO-BTBT**, and *m* **-C**_**7**_ **CO-BTBT**, are shown in Figure 2. The monocarbonyl-functionalized BTBTs were synthesized on a gram scale via regioselective Friedel–Crafts acylation under ambient conditions at the 2-position of the BTBT π-core using 4-*n*-hexylbenzoyl chloride (4-*n*-C_6_H_13_–PhCOCl), benzoyl chloride (PhCOCl), and 4-*n*-octanoyl chloride (*n*-C_7_H_15_–COCl) reagents, respectively, in the presence of an AlCl_3_ Lewis acid catalyst. As the new molecules were found to have excellent solubility (*vide infra*) in common organic solvents, the purifications were performed via column chromatography to yield the final pure solids on a gram scale as single batches (∼85%, ∼71%, and ∼73% yields, respectively). While a stoichiometric amount (1.0 *equiv*) of C_7_H_15_–COCl reagent was used to prevent dicarbonyl functionalization, excess (∼5.0 *equiv*) amounts were safely used for 4-*n*-C_6_H_13_–PhCOCl and PhCOCl reagents without any dicarbonyl product formation. This suggests that the benzoyl unit deactivates the BTBT π-system toward second acylation,^18^ which is quite different from the Friedel–Crafts acylation with alkanoyl chloride (RCOCl) that typically yields dicarbonyl products in good yields (>70%).^10,13^ The molecular structure and the chemical purity of the new molecules were characterized by using ^1^H and ^13^C NMR spectroscopies, atmospheric-pressure chemical ionization mass spectrometry (APCI-MS) (Figures S1–S9), and elemental analysis. The phene-like electronic structure of the BTBT π-system, which reduces the π-electronic interaction between the outer phenyl rings, was evident in the ^1^H NMR spectra (Figures S1, S4 and S7) of the new molecular structures. In the case of ***m*** **-C**_**6**_ **PhCO-BTBT**, as shown in Figure 2, the chemical shifts of the nonexchangeable aromatic protons “e, g,” which are on the outer phenyl ring adjacent to the 4-*n*-hexylbenzoyl unit, show downfield shifts of ∼0.5 ppm relative to those of the unfunctionalized BTBT. However, the other outer phenyl ring protons “a–d” show minimal changes in their chemical shifts. This indicates a reduced electron density (π-electron deficiency) on one side of the BTBT unit due to the asymmetric electron-withdrawing effect of the monocarbonyl functionalization. The π-density on the other outer phenyl side remains similar to that of the parent BTBT π-system. The asymmetric π-electronic structure in ***m*** **-C**_**6**_ **PhCO-BTBT** leads to a large ground-state dipole moment (Figure 2a, μ_g_ = 3.17 D) and solvent polarity-dependent optoelectronic characteristics with a single excitonic character and a significant excited-state (S_1_) dipole moment (μ_e_) of 12.69 D (*vide infra*). Moreover, the π-backbone demonstrates a significant degree of polarizability, as detailed below.

**Figure 2:**
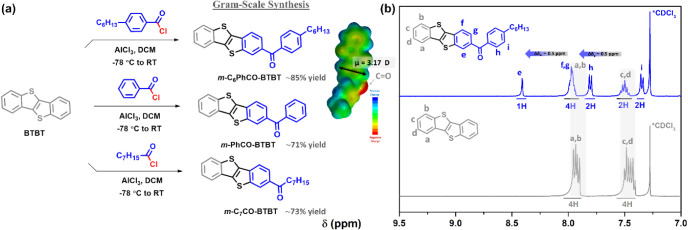
(a) Gram-scale syntheses of new monocarbonyl molecules, ***m*** **-C**_**6**_ **PhCO-BTBT**, ***m*-** **PhCO-BTBT**, and ***m*-** **C**_**7**_ **CO-BTBT**. The inset shows the DFT­(B3LYP/6–31G**)-calculated electrostatic potential map for ***m*** **-C**_**6**_ **PhCO-BTBT** and the ground-state dipole moment (μ_g_ = 3.17 D). (b) ^1^H NMR spectra of ***m*** **-C**_**6**_ **PhCO-BTBT** (top) and the parent **BTBT** (bottom) molecules in CDCl_3_ showing the nonexchangeable aromatic protons “a–i” and the downfield shifts for “e and g” (Δδ ∼ + 0.5 ppm).

On the basis of the thermogravimetric analysis, the new monocarbonyl BTBTs exhibit high thermolysis onset temperatures (T_onset_ ≈ 5% weight loss) of 313–351 °C and nearly quantitative decomposition behavior (Figure S10). The inclusion of both alkyl and aryl substitutions in ***m*** **-C**_**6**_ **PhCO-BTBT** increases its T_onset_ by ∼35–40 °C compared to that of ***m*** **-PhCO-BTBT** and ***m*** **-C**_**7**_ **CO-BTBT**, suggesting enhanced thermal stability. As shown in Figure S11, differential scanning calorimetry (DSC) measurements exhibit one major endothermic melting transition for each compound at 180.5 °C (84.45 J/g for ***m*** **-C**_**7**_ **CO-BTBT**), 225.0 °C (103.51 J/g for ***m*** **-PhCO-BTBT**), and 152.7 °C (85.85 J/g for ***m*** **-C**_**6**_ **PhCO-BTBT**). Only in the case of ***m*** **-C**_**7**_ **CO-BTBT**, an endothermic transition prior to melting was observed at 120.8 °C (27.32 J/g), indicating a liquid crystal phase transition.^10–12,18^ The corresponding crystallizations were evident in the cooling scans. The melting temperature of ***m*** **-C**_**6**_ **PhCO-BTBT** is ∼30–35 °C higher than those of the previously disclosed dialkyl-BTBTs.^10^ This increase stems from the removal of one flexible alkyl chain and the addition of a polar carbonyl and a rigid aryl unit, creating an extended π-framework with stronger solid-state cohesive energetics. Despite its higher melting temperature and the removal of one of the alkyl substituents, ***m*** **-C**_**6**_ **PhCO-BTBT** exhibits a much higher solubility as compared to well-known highly soluble dialkyl-BTBTs (e.g., **C**_**6/8**_ **-BTBTs**:^10^ 70–80 mg·mL^–1^). ***m*** **-C**_**6**_ **PhCO-BTBT** shows an instantaneous dissolution behavior at room temperature in chloroform with a remarkable solubility of 176.0 mg·mL^–1^. This corresponds to a molarity of 0.41 M, which, to the best of our knowledge, is the highest solubility ever reported for a high performance organic semiconductor.^19–25^ Note that this is >20–40× higher than those of the previously reported **C**_**n**_ **-BTBT-Ph**s (solubility <9–11 mg·mL^–1^)^11^ and **BTBT-Ph–C**_**n**_s (solubility ≪5–10 mg·mL^–1^),^12^ which carry the same alkyl and phenyl fragments on the BTBT π-system. This suggests that our asymmetric mono­(aryl)­carbonyl design is very effective to induce extreme solubility in common organic solvents. The molar solubility achieved for ***m*** **-C**_**6**_ **PhCO-BTBT** (0.41 M) is approximately twice the combined solubility values obtained for either an alkyl or an aryl substituent alone in our other synthesized monocarbonyl-BTBTs (0.16 M for ***m*** **-C**_**7**_ **CO-BTBT** and 0.06 M for ***m*** **-PhCO-BTBT**). These observations suggest that the structure of ***m*** **-C**_**6**_ **PhCO-BTBT** provides a unique molecular architecture for developing stronger interactions with solvent molecules.

### Single-Crystal Structure Analysis

Light yellow crystals of ***m*** **-C**_**6**_ **PhCO-BTBT**, suitable for single-crystal X-ray diffraction analysis, were grown using the solvent layering method (dichloromethane–ethanol). The molecule crystallizes in the orthorhombic space group P*bca* with an extremely elongated crystallographic *c*-axis (*a* = 7.99 Å, *b* = 11.81 Å, and *c* = 47.27 Å). As shown in Figure 3a, the BTBT π-system adopts a substantially coplanar backbone arrangement, and the carbonyl unit exhibits an out-of-plane torsion of 24.48°. A large dihedral angle of 51.64° was measured between the planes intersecting the phenyl units of the BTBT and the monofunctionalization part. These angles align with those in the DFT-optimized molecular geometry (23.91° and 49.48°, respectively). Along the crystallographic *c*-axis, a layer-by-layer stacking motif was observed, consisting of alternately stacked “BTBT-CO-Ph” π- and “-*n*-C_6_H_13_” σ-layers (Figure 3b). The π-layer extends into the *a*- and *b*-axes through multiple intermolecular interactions of varied origins. As shown in Figure 3c-left, the antiparallel arrangement of the asymmetric backbones facilitates face-to-face π-π interactions (3.62–3.73 Å) along the *a*-axis. Additionally, a herringbone motif (Figure 3c-right) was observed in the *ab*-plane, showing multiple edge-to-face interactions (3.01–3.64 Å: S···S/C = 3.60/3.64 Å, S···H–C­(BTBT) = 3.01 Å, (BTBT)­C···H–C­(BTBT) = 3.31 Å) and nonclassical hydrogen bonds involving the carbonyl unit. Hirshfeld surface analysis reveals the presence of these multiple weak-to-moderate nonclassical hydrogen bonds ((BTBT)­C–H···O = C (2.64 Å, ∠(D–H···A) = 175.6°) and (Ph)­C–H···O = C (2.49 Å, ∠(D–H···A) = 128.6° and 2.69 Å, ∠(D–H···A) = 119.4°) < *r*_vdw_(H) + *r*_vdw_(O) = 2.72 Å) between the carbonyl unit and the neighboring BTBT and phenyl units (Figure 3d). These O···H interactions constitute 6.5% of the total Hirshfeld surfaces and highlight the critical role of phenyl substituents in promoting nonclassical hydrogen bonds with carbonyl units (Figure 3e).^26^ All S-based interactions (i.e., S···S, S···C, and S···H; no S···O interactions) constitute 16.1% of the total Hirshfeld surfaces. Finally, C···H interactions constitute 15.3% of the total Hirshfeld surfaces.

**Figure 3:**
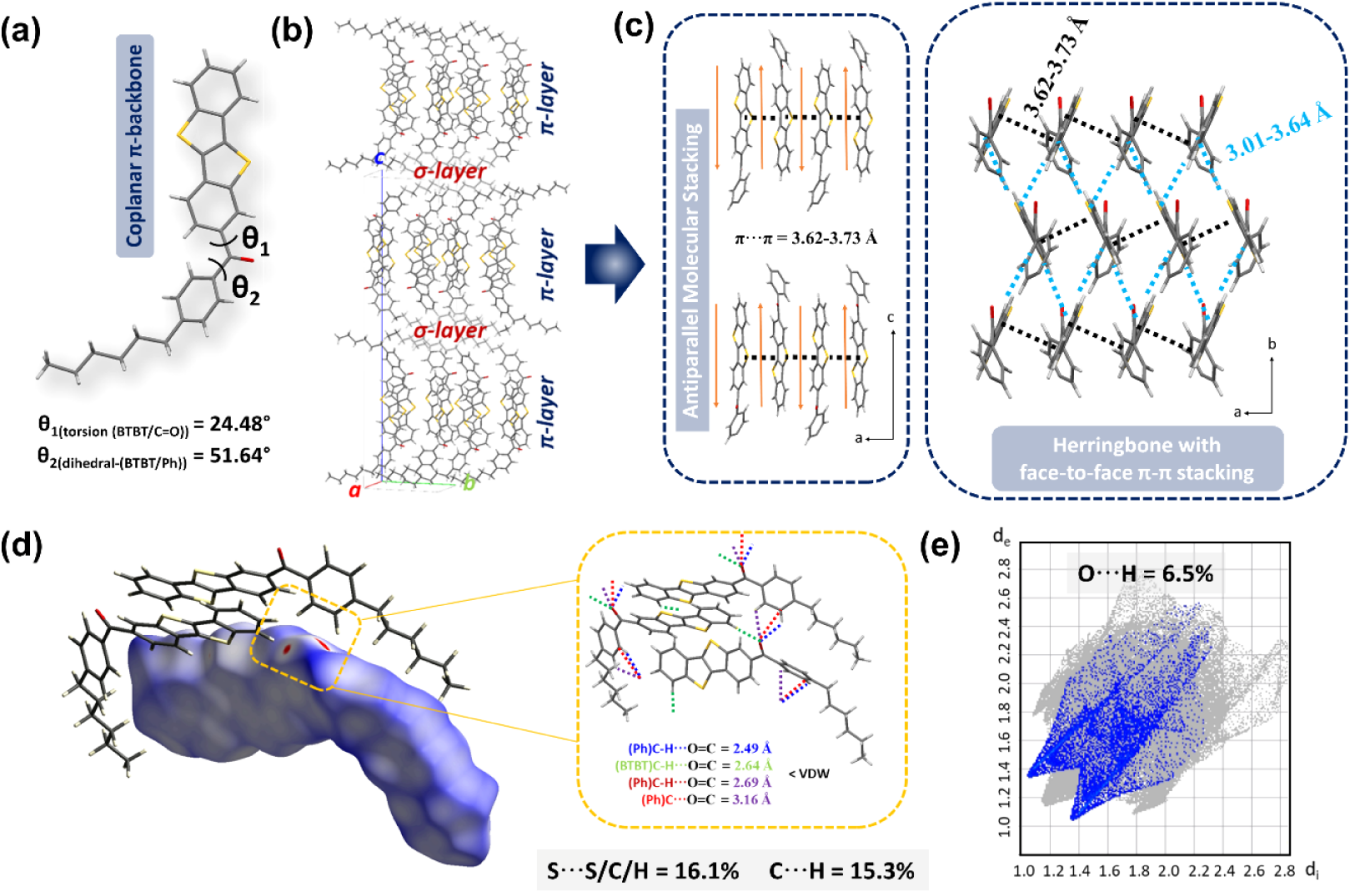
(a) Capped-stick drawings of the crystal structure of ***m*** **-C**_**6**_ **PhCO-BTBT** showing the corresponding dihedral and torsion angles and π- backbone coplanarity. (b,c) Perspective views of the molecular arrangements along the *c*-axis showing the alternately stacked layers and along the *ac*- and *ab*-planes showing the antiparallel molecular stacking (note alkyl chains are omitted for clarity only in panel c) and herringbone motifs, respectively, with the observed π-interaction distances. (d) Hirshfeld surfaces of ***m*** **-C**_**6**_ **PhCO-BTBT** mapped with d_norm_ representing three major hydrogen bonding and one O···C interactions based on carbonyl functionality. The percentage contributions of S···S/C/H and C···H interactions in the herringbone packing are provided. (e) The 2D fingerprint plot representing the major O···H nonclassical hydrogen bonding interactions.

### Hansen Solubility Parameters and Structure–Solubility Relationships

As demonstrated in Figure 4a, based on our analysis of the physicochemical properties of previously reported (i.e., **C**_**n**_ **-BTBT**,^10^ **C**_**n**_ **-BTBT-Ph**,^11^ **DPh-BTBT**,^7^ and **BTBT-Ph–C**_**n**_^12^) and our synthesized (i.e., **BTBT**,^13^ **D­(Ph**_**F**_ **CO)-BTBT**,^13^ **D­(C**_**7**_ **CO)-BTBT**,^13^ *m* **-C**_**7**_ **CO-BTBT**, and *m* **-PhCO-BTBT**) BTBT molecules, a clear inverse correlation is evident between the melting temperature and the maximum molecular solubility.^11^ Nevertheless, the solubility value achieved with ***m*** **-C**_**6**_ **PhCO-BTBT** is significantly higher (shown with a gray arrow in Figure 4a) than the expected trend value. To gain insights into the molecular design that enables ***m*** **-C**_**6**_ **PhCO-BTBT**s record-high solubility behavior, we studied the Hansen solubility parameters, along with the comparative molecules ***m*** **-PhCO-BTBT** and ***m*** **-C**_**7**_ **CO-BTBT**. The latter two molecules allow for a direct comparison with ***m*** **-C**_**6**_ **PhCO-BTBT**, thereby revealing the individual effects of the aryl and alkyl units. The solubility of the new molecules was explored in a diverse set of organic solvents, spanning nonpolar solvents, chlorinated alkanes, aromatics, alcohols, polar aprotic solvents, terpenes, and esters, having a wide range of dispersion (δ_D_ = 14.5–20), polar (δ_P_ = 0–21.7), and hydrogen-bonding (δ_H_ = 0–22.3) interactions. UV–vis absorption spectroscopy (see Figure S12 for calibration curves) and gravimetric methods were employed for this purpose (see the Experimental details).^27^ As shown in Table 1, the new molecules exhibited solubility across a wide range, from complete insolubility to as high as 176.0 mg·mL^–1^ in 28 different organic solvents. While chlorinated alkanes and aromatics yield the highest solubilities for all three molecules, reasonable solubility values are recorded in polar aprotic solvents, esters, and terpenes as well. On the basis of a threshold concentration value of 4.0 mg·mL^–1^ at room temperature, solubility scores of “1” (indicating a good solvent) and “0” (indicating a nonsolvent) are assigned. Note that this is a usual spin-coating concentration to yield semiconducting thin films.^27,28^ The solubility spheres are calculated in the HSPiP program using the Genetic algorithm.^29,30^ The genetic algorithm is a heuristic stochastic global optimization method successfully applied in diverse fields ranging from vehicle routing to quantum control of atomic/molecular dynamics.^31^ For our current monocarbonyl BTBTs, this method yielded the best fitting accuracies (0.929–1.000) with minimum errors in solvent selection, as it aims to find the least number of incorrect solvents within the smallest possible radius.

**Figure 4:**
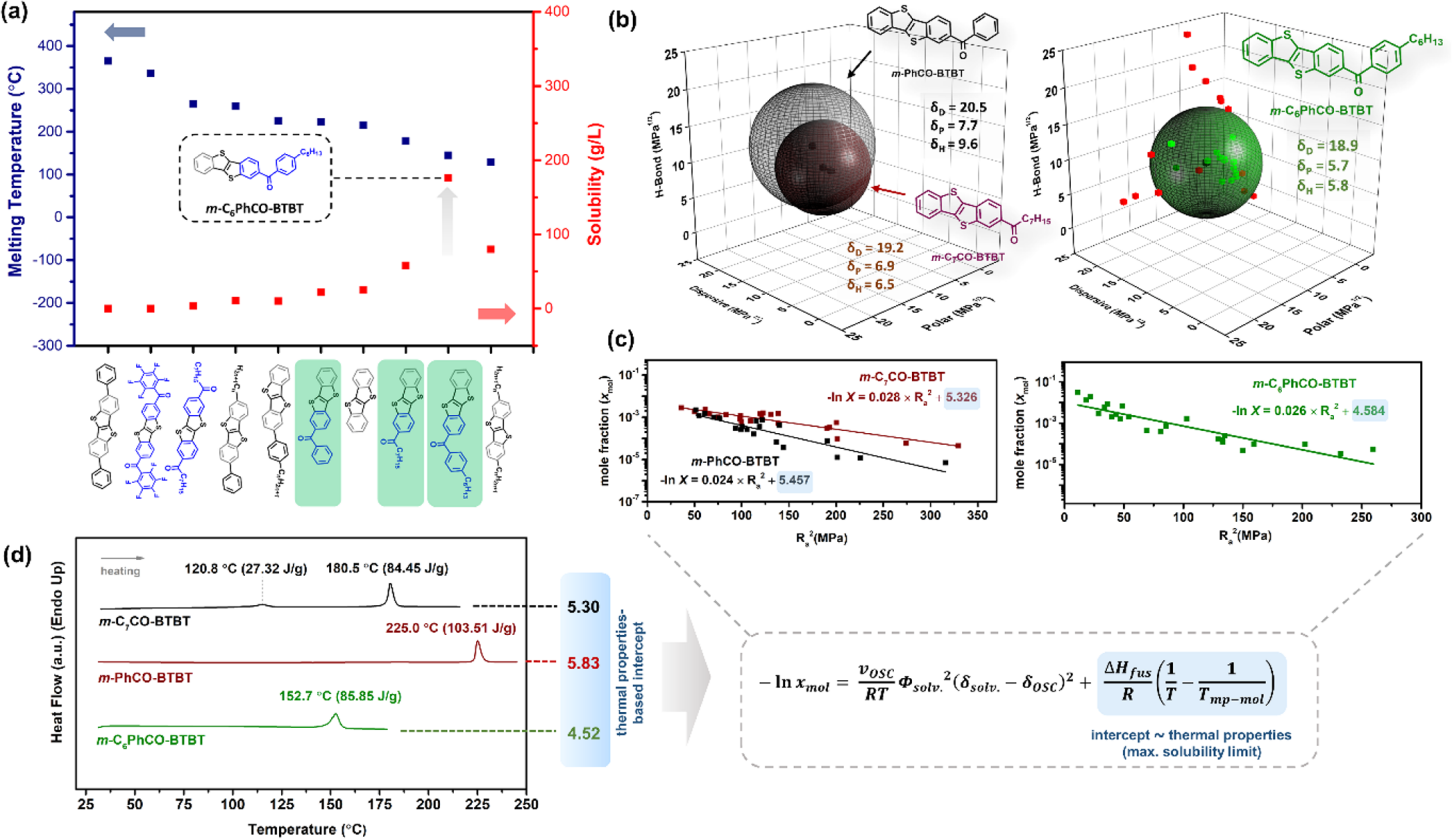
(a) The melting temperatures (blue squares) and maximum solubilities (red squares) of previously developed BTBT-based semiconductors, **DPh-BTBT**,^7^ **D­(Ph**_**F**_ **CO)-BTBT**,^13^ **D­(C**_**7**_ **CO)-BTBT**,^13^ **C**_**n**_ **-BTBT-Ph**,^11^ **BTBT-Ph–C**_**n**_,^12^ **BTBT**,^13^ **C**_**n**_ **-BTBT**,^10^ shown from left to right, along with our synthesized molecules ***m*** **-PhCO-BTBT**, ***m*** **-C**_**7**_ **CO-BTBT**, and ***m*** **-C**_**6**_ **PhCO-BTBT**, respectively, shown in green from left to right. For solids of **DPh-BTBT** and **BTBT-Ph–C**_**n**_, which have been purified and processed via nonsolution-based techniques, solubility values are taken as <10 mg·mL^–1^. The solubility values of **D­(Ph**_**F**_ **CO)-BTBT**, **D­(C**_**7**_ **CO)-BTBT**, and **BTBT** solids (in chloroform) were determined in our laboratory. (b) Hansen solubility spheres and parameters of ***m*** **-PhCO-BTBT**, ***m*** **-C**_**7**_ **CO-BTBT**, and ***m*** **-C**_**6**_ **PhCO-BTBT**, as determined using the Genetic algorithm (HSPiP Program) with a solubility limit of 4 mg·mL^–1^. The Hansen solubility parameters (δ_D_/δ_P_/δ_H_) are in MPa^1/2^, and the bad and the good solvents are shown in the 3D Hansen solubility space with red and green spheres, respectively, for ***m*** **-C**_**6**_ **PhCO-BTBT**. (c) The logarithmic correlations between the semiconductor solubility in the mole fraction unit (*x*_mol_) and the squared solute–solvent distance (R_a_^2^ in the HSP space (correlation coefficient ≈ −0.91). The corresponding equations are derived based on linear fittings and correspond to the Hansen-adapted Scatchard–Hildebrand regular solution theory equation (*v*_osc_ is the molar volume of the subcooled liquid of the pure molecular solid, Φ_solv_ is the solvent volume fraction (≈1 for dilute solutions), ΔH_fus_ is enthalpy of fusion, T_mp‑mol_ is the melting temperature, *R* is the gas constant, and*T* is the solubility measurement temperature (absolute)). (d) The differential scanning calorimetry (10 °C·min^–1^ heating ramp under N_2_) heating scans of ***m*** **-PhCO-BTBT**, ***m*** **-C**_**7**_ **CO-BTBT**, and ***m*** **-C**_**6**_ **PhCO-BTBT** solids showing the temperature and enthalpy values for the corresponding thermal transitions from solid to isotropic liquid.

**Table 1:** Solubility Values (in mg·mL^–1^) of ***m*** **-C**_**7**_ **CO-BTBT**, ***M*** **-PhCO-BTBT**, and ***m*** **-C**_**6**_ **PhCO-BTBT** in 28 Different Organic Solvents Determined via Spectroscopic (UV-Vis Absorption) and Gravimetric Methods, and the Solvent Hansen Solubility Parameters (δ_D_, δ_P_, δ_H_ in MPa^0.5^)­^a^

	Hansen Solubility Parameters, MPa^0.5^			Solubility, mg·mL^–1^ (Solubility Score)		
Solvent	δ_D_	δ_P_	δ_H_	*m* **-**C_7_CO-	*m*-PhCO-	*m*-C_6_PhCO-
**Aromatics**						
Benzene	18.4	0	2.0	5.50 (1)	3.02 (0)	18.2 (1)
Toluene	18.0	1.4	2.0	7.31 (1)	2.37 (0)	34.0 (1)
*o*-Xylene	17.6	1	3.1	3.07 (0)	1.83 (0)	21.3 (1)
**Alcohols**						
1-Butanol	16	5.7	15.8	0.34 (0)	0.12 (0)	0.73 (0)
*tert*-Butanol	15.2	5.1	14.7	0.20 (0)	0.13 (0)	0.54 (0)
Ethanol	15.8	8.8	19.4	0.10 (0)	Insoluble	0.24 (0)
Ethylene glycol	17	11	26	Insoluble	Insoluble	0.04 (0)
Methanol	14.7	12.3	22.3	0.08 (0)	0.06 (0)	0.26 (0)
2-Propanol	15.8	6.1	16.4	0.48 (0)	Insoluble	0.26 (0)
**Polar aprotic**						
Acetone	15.5	10.4	7.0	1.57 (0)	0.75 (0)	2.70 (0)
Acetonitrile	15.3	18	6.1	0.15 (0)	0.08 (0)	0.77 (0)
Diethyl ether	14.5	2.9	4.6	1.03 (0)	0.25 (0)	2.94 (0)
Dimethyl sulfoxide (DMSO)	18.4	16.4	10.2	0.60 (0)	1.47 (0)	1.52 (0)
N,N-Dimethylformamide (DMF)	17.4	13.7	11.3	2.98 (0)	4.03 (1)	9.26 (1)
1,4-Dioxane	17.5	1.8	9	4.20 (1)	4.02 (1)	4.8 (1)
Ethylene carbonate	18	21.7	5.1	0.12 (0)	0.86 (0)	0.36 (0)
Methyl *iso*-butyl ketone (MIBK)	15.3	6.1	4.1	2.40 (0)	1.19 (0)	7.21 (1)
*N*-Methyl-2-pyrrolidone (NMP)	18	12.3	7.2	4.02 (1)	7.97 (1)	31.2 (1)
Propylene carbonate	20	18	4.1	0.24 (0)	0.27 (0)	0.50 (0)
Tetrahydrofuran	16.8	5.7	8.0	28.3 (1)	6.44 (1)	102.5 (1)
**Esters**						
*n*-Amyl acetate	15.8	3.3	6.1	2.68 (0)	0.84 (0)	7.72 (1)
Ethyl Acetate	15.8	5.3	7.2	1.61 (0)	0.90 (0)	8.80 (1)
Propylene glycol monoethyl ether acetate	15.6	6.3	7.7	1.53 (0)	0.90 (0)	5.05 (1)
**Chlorinated alkanes**						
Chloroform	17.8	3.1	5.7	58.3 (1)	22.2 (1)	176.0 (1)
Methylene dichloride	17	7.3	7.1	25.1 (1)	6.32 (1)	87.5 (1)
**Nonpolar**						
Cyclohexane	16.8	0	0.2	0.53 (0)	0.04 (0)	1.60 (0)
Hexane	14.9	0.0	0.0	0.23 (0)	Insoluble	0.56 (0)
**Terpenes**						
*d*-Limonene	17.2	1.8	4.3	2.09 (0)	0.54 (0)	8.02 (1)

a The solubility scores “1” (for good solvent) and “0” (for nonsolvent) are assigned based on the threshold concentration value of 4.0 mg·mL^–1^.

As shown in Figure 4b, the HSPs for ***m*** **-C**_**6**_ **PhCO-BTBT** are determined to be δ_D_ = 18.9 MPa^1/2^, δ_P_ = 5.7 MPa^1/2^, and δ_H_ = 5.8 MPa^1/2^ with an interaction radius (*R*_0_) of 8.0 MPa^1/2^. Considering the specific energy densities (δ^2^), the dispersion-based interactions appear to be the major contributor to the solid-state cohesive energetics. It is crucial to note that while Hansen’s original solubility theory does not specifically identify π-interactions, the δ_D_ term derived herein indeed includes π-interactions (i.e., π···π, C–H···π, and S···π) between extended monocarbonyl π-systems, along with the dispersion interactions between aliphatic substituents.^32,33^ The prevalence of dispersion interactions in the HSPs was additionally verified through the group contribution approach, as depicted in Figure S13. This technique, utilized in the HSPiP using Neural Network methods, determines the HSPs by calculating contributions from specific molecular constituents, such as aromatics, aliphatics, and functional groups.^34,35^ On the other hand, the HSPs for the comparative molecules ***m*** **-PhCO-BTBT** and ***m*** **-C**_**7**_ **CO-BTBT** are determined to be δ_D_ = 20.5 MPa^1/2^/δ_P_ = 7.7 MPa^1/2^/δ_H_ = 9.6 MPa^1/2^ (*R*_0_ = 8.9 MPa^1/2^) and δ_D_ = 19.2 MPa^1/2^/δ_P_ = 6.9 MPa^1/2^/δ_H_ = 6.5 MPa^1/2^ (*R*_0_ = 6.8 MPa^1/2^), respectively (Figure 4b). Transititoning from either only phenyl (as in ***m*** **-PhCO-BTBT**) or only alkyl (as in ***m*** **-C**_**7**_ **CO-BTBT**) substituents to an alkyl-aryl substituent combination (as in ***m*** **-C**_**6**_ **PhCO-BTBT**) results in a decrease in δ_P_ (−Δ = 1.2–2.0 MPa^1/2^) and δ_H_ (−Δ = 0.7–3.8 MPa^1/2^) interaction parameters. This effect arises from the relative contributions of these substituents and the carbonyl unit to overall cohesive energetics, which are closely tied to their relative volumes compared to the total molecular volumes in 3D crystal structures. Because δ_P_ and δ_H_ are directly linked to polarity and hydrogen bonding ability, respectively, the observed reductions are evidently due to volume dilution of the carbonyl unit. This may enable fine-tuning of δ_P_ and δ_H_ values for specific solubility characteristics, which could be used as a key design principle in other π-systems as well. Changing the substituent from an alkyl to an aryl unit when transitioning from ***m*** **-C**_**7**_ **CO-BTBT** to *m* **-PhCO-BTBT** results in an increase of δ_D_ by 1.3 MPa^1/2^ and δ_H_ by 3.1 MPa^1/2^. Given the similar molecular weights of these molecules, the increase in δ_D_ reflects a more condensed structure of the phenyl aromatic ring compared to that of the linear aliphatic heptyl chain. The increase in δ_H_, on the other hand, indicates the nonclassical hydrogen bonding (C–H···π) ability of the aryl unit, which the aliphatic chain lacks.^36^ Notably, the δ_D_ and δ_H_ trends in these monocarbonyl BTBT molecules resemble those observed for hexane, cyclohexane, and benzene, respectively (δ_D_/δ_H_ = 14.9/0.0 MPa^1/2^ → 16.8/0.2 MPa^1/2^ → 18.4/2.0 MPa^1/2^).

On the basis of the calculated HSPs, chloroform, which appears to be the best solvent for all three molecules, gives the closest solute–solvent interaction distances (*R*_a_ = (4Δδ_D_^2^ + Δδ_P_^2^ + Δδ_H_^2^)^1/2^), as compared to the rest of the solvents. More importantly, the *R*_a_ value gradually decreases to 8.05 MPa^1/2^ (for ***m*** **-PhCO-BTBT**), 4.79 MPa^1/2^ (for ***m*** **-C**_**7**_ **CO-BTBT**), and 3.36 MPa^1/2^ (for ***m*** **-C**_**6**_ **PhCO-BTBT**), which aligns well with the corresponding gradual solubility increase of 0.06 → 0.16 → 0.41 M (Table 2). Due to the 4-fold effect of the dispersion parameter (Δδ_D_) on R_a_, ***m*** **-C**_**6**_ **PhCO-BTBT** with the closest δ_D_ value to that of chloroform yields the highest solubility. This highlights a key design advantage of alkyl–aryl substitution over alkyl or aryl substitution alone, particularly in enhancing the molecular solubility of a rigid π-system through dispersion interactions.

**Table 2:** Summary of Hansen Solubility Parameters (δ_D_, δ_P_, and δ_H_ in MPa^1/2^), Interaction Radii (*R*_0_ in MPa^1/2^), and Solubilities (at 25 °C) in Chloroform for ***m*** **-PhCO-BTBT**, ***m*** **-C**_**7**_ **CO-BTBT**, and ***m*** **-C**_**6**_ **PhCO-BTBT**, and the Corresponding HSP Interaction Distances (*R_a_* in MPa^1/2^) with Respect to Chloroform

	HSPs and Interaction Radii^a^					Solubility in Chloroform	
Compounds	δ_D_	δ_P_	δ_H_	R_0_	Fitting, WI/WO	R_a_ ^b^	Solubility^c^ (mg·mL^–1^/M)
***m*** **-PhCO-BTBT**	20.5	7.7	9.6	8.9	1.000, 0/0	8.05	22.2/0.06
***m*** **-C**_**7**_ **CO-BTBT**	19.2	6.9	6.5	6.8	0.929, 0/2	4.79	58.0/0.16
***m*** **-C**_**6**_ **PhCO-BTBT**	18.9	5.7	5.8	8.0	0.964, 0/1	3.36	176.0/0.41

a Hansen solubility parameters and interaction radii are determined by the HSP sphere approach using the genetic algorithm. Fitting and the number of wrong-in/out (WI/WO) solvents are also provided for each semiconductor.

b *R*_a_ is calculated from the equation *R*_a_ = (4­(δ_D,1_ – δ_D,2_)^2^ + (δ_P,1_ – δ_P,2_)^2^ + (δ_H,1_ – δ_H,2_)^2^)^1/2^ in which “1” is the compound and “2” is chloroform.

c Solubilities in chloroform are measured using gravimetric and spectroscopic methods.

The excellent solubility of ***m*** **-C**_**6**_ **PhCO-BTBT** is compared with that of previously reported **BTBT-Ph–C**_**6**_ **/C**_**12**_ semiconductors, which have exactly the same subunits and “π-π-σ” sequence as our molecule but lack an electron-withdrawing carbonyl unit. Note that one of these molecules has an even longer alkyl chain (-*n*-C_12_H_25_). In earlier studies with **BTBT-Ph–C**_**6**_ **/C**_**12**_,^12^ the material purifications and OFET thin-film depositions have been performed via nonsolvent-based techniques (i.e., vacuum sublimation and physical vapor deposition). It has also been noted that structural engineering is necessary to solubilize these compounds. Therefore, it is evident that the solubility of **BTBT-Ph–C**_**6**_ **/C**_**12**_ molecules is well below ∼5–10 mg·mL^–1^, which is typically the minimum requirement for solution processing.^37^ This vis-à-vis experimental comparison clearly demonstrates that inserting a monocarbonyl unit between the π-core and the aryl substituent, transitioning from “π-π-σ” to a “π-CO-π-σ” molecular architecture, notably enhances solubility. On the other hand, transitioning from ***m*** **-C**_**7**_ **CO-BTBT** to **D­(C**_**7**_ **CO)-BTBT**, which was synthesized in our previous study,^13^ although an additional solubilizing alkyl substituent is introduced, the molecular solubility decreases more than 10-fold (58.3 mg·mL^–1^ → 3.5 mg·mL^–1^). One may also compare the dicarbonyl semiconductor **D­(C**_**7**_ **CO)-BTBT** with the analogous noncarbonyl derivatives of **(di)-C**_**7/8**_ **-BTBT** that have the same alkyl substituents.^10,13^ Despite having the same alky substituents, dicarbonyl functionalization significantly decreases molecular solubility (70–80 mg·mL^–1^ → 3.5 mg·mL^–1^). Therefore, it is evident that while single carbonyl functionalization greatly improves solubility, transitioning from an asymmetric monocarbonyl to a symmetric dicarbonyl configuration (“π-CO-substituent” → “substituent-CO-π-CO-substituent”) results in a significant decrease in molecular solubility.

Theoretical calculations aided by DFT have shown that monocarbonyl functionalization in BTBT results in a substantial ground-state molecular dipole (μ_g_ = 3.17 D, Figure 2a). This value is significantly higher than those observed for mono/disubstituted BTBTs mentioned earlier, which exhibit μ_g_ = 0 D for **(di)-C**_**6/8**_ **-BTBTs** and **D­(C**_**7**_ **CO)-BTBT**, 0.45 D for **C**_**n**_ **-BTBT-Ph**, and 0.63 D for **BTBT-Ph–C**_**n**_ (Figure S14). This ground-state molecular dipole is expected to lead to strong dipolar interactions with solvent molecules, particularly with polar green solvents.^38,39^ In a solvent medium, an increased molecular dipole (Δμ_g_ ≈ + 1.3 D in tetrahydrofuran (ε = 7.4) and +1.6 D in dimethylformamide (ε = 37)) was calculated for ***m*** **-C**_**6**_ **PhCO-BTBT**, whereas the dicarbonyl-functionalized and aryl/alkyl-substituted BTBTs showed minimal changes (Δμ_g_ ≈ 0–0.1 D) (Figure S14). The observed increase of μ_g_ in a solvent medium is not due to a conformational change; it is rather due to an induced dipole via π-polarization. This is confirmed by repeating the DFT calculation in the gas phase using the solvent-optimized molecular geometry. Furthermore, the polarizability of the BTBT molecular π-system was shown to be enhanced in all directions after monocarbonyl functionalization, with the most significant increase along the *z*-axis (19.10 Å^3^ → 22.08 Å^3^) (Figure S14). The polarizability of the new mono­(aryl)­carbonyl-functionalized π-architecture is expected to lead to enhanced dispersion and induced dipole interactions with solvent molecules.^33,40,41^ DFT studies (Figure S14) also showed that the monocarbonyl insertion in ***m*** **-C**_**6**_ **PhCO-BTBT** twists the phenyl ring out of the BTBT π-plane (θ_dihedral_ = 51°) as compared to low-solubility molecules in which there is a direct BTBT-Ph bond (θ_dihedral_ = 2.5/6.2° in **BTBT-Ph–C**_**n**_ **/C**_**n**_ **-BTBT-Ph** based on single-crystal^11,42^ ). This reduces the structural rigidity and solid-state cohesive energetics in ***m*** **-C**_**6**_ **PhCO-BTBT**,^23^ and it contributes to enhanced solubility.^38^ Consistently, all three Hansen parameters for ***m*** **-C**_**6**_ **PhCO-BTBT**, despite being a larger molecular system, are lower than those of its nonaryl counterpart *m* **-C**_**7**_ **CO-BTBT** (*vide supra*), confirming a lower cohesive energy density.

### Thermodynamic Correlation of the Molecular Solubility with Thermal Properties

For our new monocarbonyl BTBTs in the HSP space, a quantitative thermodynamic relationship is explored between the measured solubility values and the calculated interaction distances (*R*_a_s). One could also consider this quantitative relationship as providing further confirmation of the accuracy of the calculated Hansen parameters. As depicted in Figure 4c, strong negative correlations were established when the solubility mole fraction (*x*_mol_) in a particular solvent is logarithmically plotted against the specific solvent-molecule R_a_^2^ value. A different regression equation was established for each molecule, as expected. These equations follow a modified version of the original equation proposed by Scatchard and Hildebrand for regular solutions, adapting Hansen parameters as shown in the dashed box in Figure 4c.^43,44^ In this modification, *R*_a_ values better define molecule-solvent interactions compared to the simpler Hildebrand solubility parameters distance.^43,45^ On the other hand, we note that the Flory–Huggins correction term for the entropy of mixing in this equation is taken to be significantly smaller than the HSP term. Consequently, it has been excluded from the calculations.^45^ Since the slopes for all three molecules are similar (0.024–0.028), the intercept value becomes the most critical aspect of these equations, which indeed represents the maximum molecular solubility in an ideal solvent as *R*_a_^2^ approaches 0. According to textbook equations in physical chemistry, this part actually defines a molecule’s solubility based on the ideal solution approach, utilizing the solid-to-isotropic liquid transition properties of enthalpy of fusion (ΔH_fus_) and melting temperature (*T*_mp‑mol_). The intercept values are found to decrease as 5.457, 5.326, and 4.584 for ***m*** **-PhCO-BTBT**, ***m*** **-C**_**7**_ **CO-BTBT**, and ***m*** **-C**_**6**_ **PhCO-BTBT**, respectively. This trend matches well with the observed solubility trend in organic solvents, as ***m*** **-C**_**6**_ **PhCO-BTBT**, having the smallest intercept value, exhibits the highest solubility. More importantly, these intercept values could be estimated also from the thermal properties measured via DSC measurements. Using enthalpy values and transition temperatures determined via DSC (Figure 4d), the intercept values are closely estimated as 5.83, 5.30, and 4.52 for ***m*** **-PhCO-BTBT**, ***m*** **-C**_**7**_ **CO-BTBT**, and ***m*** **-C**_**6**_ **PhCO-BTBT**, respectively. To the best of our knowledge, these values are estimated for the first time in the literature from both Hansen-based solubility studies and thermal characterizations. These quantitative correlations and their strong link to thermal properties could pave the way for new solubility research in organic semiconductors, both fundamental and applied. For example, computer-aided rapid solvent screening could estimate solubility in specific solvents or mixtures before testing, offering unique practical benefits in various industrial settings.

### Hansen-Based and Theoretical Cohesive Energy Densities

The δ_T_^2^ value is defined as δ_D_^2^ + δ_P_^2^ + δ_H_^2^, and it represents the total cohesive energy effective per molar volume (*E*_cohesive_/*V*_molar_) of the molecules studied. For ***m*** **-PhCO-BTBT**, ***m*** **-C**_**7**_ **CO-BTBT**, and ***m*** **-C**_**6**_ **PhCO-BTBT**, these values decrease in the order 571.7, 458.5, and 423.3 MPa, respectively. This trend is consistent with the observed maximum solubilities for these molecules. Employing the obtained single-crystal lattice for ***m*** **-C**_**6**_ **PhCO-BTBT**, the total cohesive energy density was computed by applying the pairwise interaction energy summation method and using previously described CE-B3LYP model energies.^46,47^ For this, the DFT method with the 6–31G­(d,p) basis set was employed, and the nearest-neighbor shell interaction energy (NNSE) was determined by summing the pairwise interaction energies of 15 molecules within a 3.8 Å distance range (Figure S15). The NNSE was calculated as 166.0 kJ/mol, which aligns closely with the HSPs-estimated total cohesive energy density of 423.3 MPa, corresponding to 142.1 kJ/mol (obtained using unit cell volume = 4461.9 Å^3^ and Z= 8). The difference is considered quite reasonable, as theoretical NNSE tends to overestimate the experimental cohesive energy (obtained from sublimation enthalpy) because energy changes associated with solid-to-gas phase molecular relaxation are not taken into account.^47^ Furthermore, the largest contribution to the NNSE comes from the dispersion interaction energies (*E*_dis_ vs *E*_ele_/*E*_pol_/*E*_rep_ in Figure S15), which aligns with the contributions of δ_D_ compared to δ_P_ and δ_H_ in the HSPs. When the observed trend in the *E*_cohesive_/*V*_molar_ values is compared with the chemical potential changes for the corresponding solid-to-liquid thermal transitions, a correlation is evident. The μ^*°*^_*(s)*_ → μ^*°*^_(*l)*_ transition (at room temperature) is related to molecular solubility and is employed in the ideal solubility expression at room temperature. The Δ*G* ^*°*^_*s→l*_ (25 °C) could be estimated based on the solid-to-liquid transitions observed in the DSC scan, using the formula Δ*G* ^*°*^_*s→l*_ *= ∑*ΔH°_trans._(1–298.15/T_trans._), where ΔH°_trans._ is the enthalpy of a specific transition and T_trans._ is the corresponding transition temperature. Notably, the Δ*G* ^*°*^_*s→l*_ values at room temperature follow the same trend as the cohesive energy densities and maximum solubilites, estimated at 14.45 kJ/mol *for* **m-PhCO-BTBT**, 13.14 kJ/mol for ***m*** **-C**_**7**_ **CO-BTBT**, and 11.21 kJ/mol for ***m*** **-C**_**6**_ **PhCO-BTBT**.

### Photophysical and Electrochemical Properties

The electronic properties of the new semiconductor were studied using UV–vis absorption and photoluminescence spectroscopies, cyclic voltammetry, and (TD-)­DFT calculations. As shown in Figure 5a, consistent with its colorless solution, ***m***-**C**_**6**_ **PhCO-BTBT** shows two low-energy absorption maxima at 327/362 nm with the onset wavelength of 391 nm (*E*_g_^opt^ = 3.17 eV) in dichloromethane. The absorption maxima and the onset wavelengths show a significant bathochromic shift (Δλ ∼ 40–55 nm) in the spin-coated thin film, and a typical vibronic structure (∼1200 cm^–1^) of an aromatic π-system is formed. This indicates that the “PhCO-BTBT” π-scaffold is coplanarized and rigidified in the solid state through intermolecular forces. The observed spectral changes suggest J-type aggregation.^13^ In this arrangement, ***m*** **-C**_**6**_ **PhCO-BTBT** π-backbones likely assume a slipped-stacked configuration through ground-state dipolar interactions.^48^ When ***m*** **-C**_**6**_ **PhCO-BTBT** is excited with an excitation wavelength of 327 or 362 nm, the recorded fluorescence spectrum shows a broad emission profile with λ_fl_^max^ at 435 nm, corresponding to large Stokes shifts of 73–108 nm. When the solvent polarity is screened from hexane (*f*(ε,*n*) = 0.001) to acetonitrile (*f*(ε,*n*) = 0.305), the fluorescence spectrum exhibits an additional bathochromic shift of ∼36 nm (Figure 5b). On the other hand, the absorption profile for ***m*** **-C**_**6**_ **PhCO-BTBT** exhibits a relatively small bathochromic shift across the same solvents (Δλ_abs_^max^ ∼6–7 nm). These solvatochromic photophysical characteristics are much more pronounced than the unfunctionalized parent BTBT π-backbone (Figure S16). This is undoubtedly the result of monocarbonyl asymmetric π-electronic structure of the new molecule, and it suggests that the radiative excited state (S_1_) possesses a higher dipole moment than the ground state. The excited-state (S_1_) dipole moment (μ_e_) could be estimated from the slope of the Lippert–Mataga plot (Stokes shifts (ν_*abs*_ - ν_*fl*_) against *f­(ε,n)*) using the DFT-calculated ground-state dipole moment (μ_g_) (see Table S4 for data details, Supporting Information).^49^ Based on this model, a good linearity between “ν_abs_-ν_fl_” and *f­(ε,n*) was found to persist across the whole solvent polarity region (hexane (*f*(ε,*n*) = 0.001) → acetonitrile (*f*(ε,*n*) = 0.305)) (Figure 5b) and yielding a single excitonic character with a relatively large excited-state dipole moment (μ_e_) of 12.69 D (∼0.26 charge·nm). The theoretical UV–vis absorption spectrum, obtained via TD-DFT, revealed that the high-intensity electronic transitions occur to the S_1_ (HOMO→LUMO) and S_3_ (HOMO–1→LUMO) states at 366.3 nm (*f* = 0.24) and 323.8 nm (*f* = 0.57) (see Table S5). This aligns perfectly with the optical absorption spectrum in the solution. The frontier orbital topographies for the unoccupied orbitals, LUMO and LUMO+1, show a complete molecular π-backbone delocalization, whereas the occupied frontier orbitals, HOMO and HOMO–1, exhibit more limited delocalization, mainly on the BTBT π-system. These observations, combined with the spectroscopic findings, suggest the presence of a hybridized local and charge transfer (HLCT) excited state for ***m*** **-C**_**6**_ **PhCO-BTBT**.

**Figure 5:**
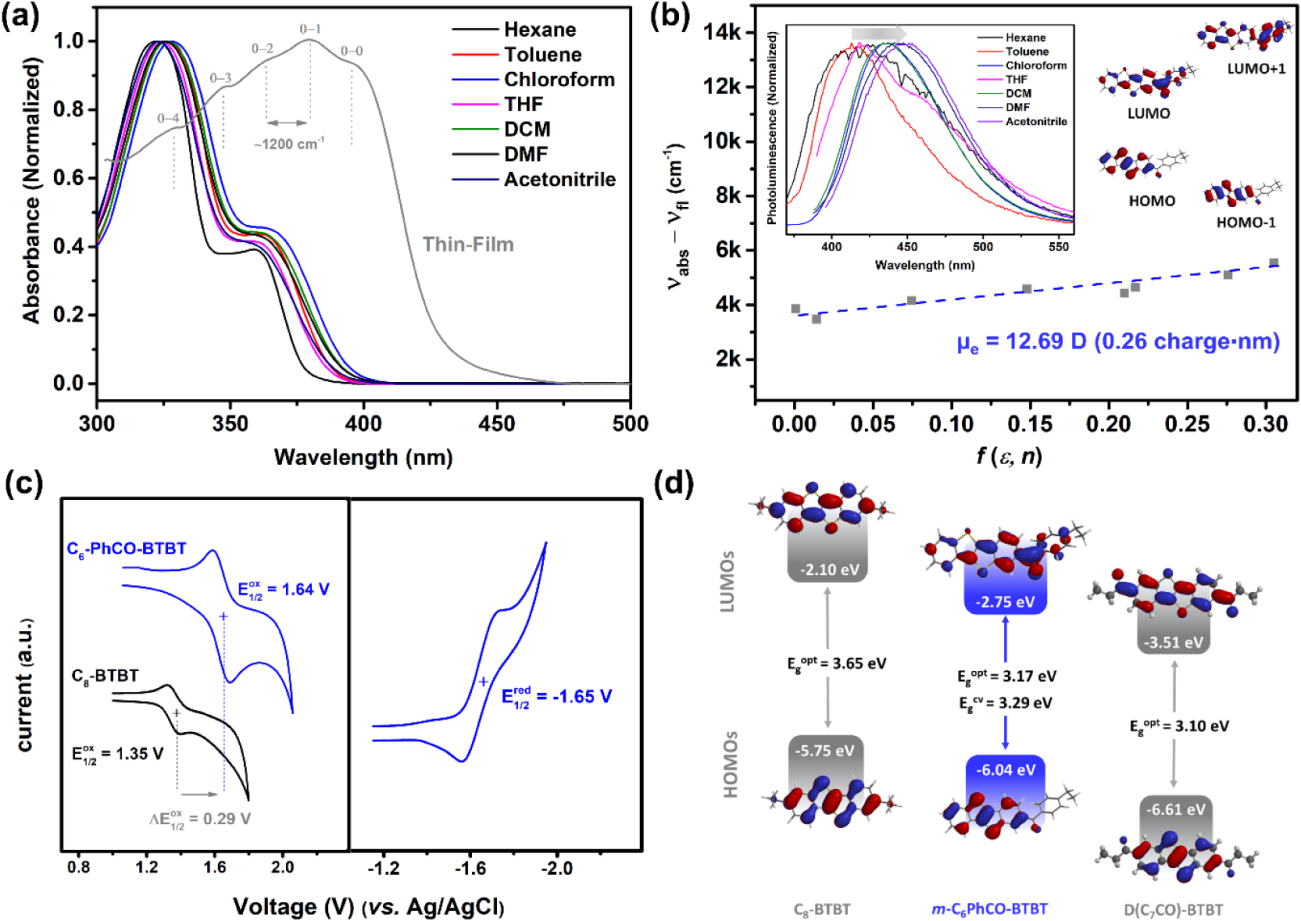
(a) Solvatochromic optical absorption spectra of ***m*** **-C**_**6**_ **PhCO-BTBT** in different solvents with increasing polarity (hexanes (*f­(ε,n)* = 0.001) → acetonitrile (*f­(ε,n)* = 0.305)) and the solid-state optical absorption spectrum of a spin-coated thin film (annealed at 120 °C) on glass, showing vibronic structure. (b) Solvatochromic Lippert-Mataga model for *m* **-C**_**6**_ **PhCO-BTBT** showing the fitted linear correlation (ν_abs_-ν_*fl*_ vs *f*(ε,n)) and the corresponding solvatochromic photoluminescence spectra in different solvents with increasing polarity (hexanes (*f­(ε,n)* = 0.001) → acetonitrile (*f­(ε,n)* = 0.305)). (c) Cyclic voltammograms of ***m*** **-C**_**6**_ **PhCO-BTBT** and **C**_**8**_ **-BTBT** (measured in house for comparison), showing oxidation and reduction peaks in 0.1 M TBAPF_6_/CH_2_Cl_2_ solution vs Ag/AgCl (3.0 M NaCl) at a scan rate of 100 mV/s. (d) The experimental HOMO and LUMO energy levels, frontier orbital topographies (DFT/B3LYP/6–31G**), and optical/electrochemical band gaps.

The cyclic voltammograms in dichloromethane solution show clear (*quasi*)­reversible oxidation and reduction peaks at +1.64 V and −1.65 V (vs Ag/AgCl), respectively (Figure 5c). Considering that the dialkyl-substituted counterpart **C**_**8**_ **-BTBT** shows only an oxidation peak at +1.35 V (vs Ag/AgCl) in the same experimental system, the presence of a reduction peak and an anodically shifted (Δ*E*_1/2_^ox^ = +0.29 V) oxidation peak undoubtedly reflects the electronic effects of mono­(aryl)­carbonyl functionalization. As shown in Figure 5d, HOMO and LUMO energy levels are estimated to be −2.75 eV and −6.04 eV, which falls between those of unfunctionalized (substituted with alkyl chains) and dicarbonyl-functionalized BTBT molecules. The electrochemical band gap is calculated to be 3.29 eV (*E*_g_^opt^ = 3.17 eV and *E*_g_^theory^ = 3.38 eV). Our results revealed that mono­(aryl)­carbonyl functionalization stabilizes the frontier molecular orbitals in different scales (Δ*E*_HOMO_ = −0.29 eV and Δ*E*_LUMO_ = −0.65 eV). This is because while mono­(aryl)­carbonyl stabilizes the HOMO only via a negative inductive (-*I*) effect (i.e., no π-orbital density on the (aryl)­carbonyl moiety), it stabilizes the LUMO via both negative resonance (-*R*) effect and π-extension on the (aryl)­carbonyl unit (Figure 5d).

### Green Solvents for Field-Effect Transistor Fabrications and Thin-Film/Electrical Characterizations

Considering the excellent solubility of ***m*** **-C**_**6**_ **PhCO-BTBT** in common organic solvents, a set of potential green solvents have been evaluated for thin-film processing. ***m*** **-C**_**6**_ **PhCO-BTBT** exhibited reasonable room temperature solubilities of 12.5 mg·mL^–1^ in ethoxybenzene (δ_D_ = 18.4, δ_P_ = 4.5, δ_H_ = 4.0), 10.4 mg·mL^–1^ in 2-methyltetrahydrofuran (δ_D_ = 16.9, δ_P_ = 5.0, δ_H_ = 4.3), 8.80 mg·mL^–1^ in ethyl acetate (δ_D_ = 15.8, δ_P_ = 5.3, δ_H_ = 7.2), and 2.70 mg·mL^–1^ in acetone (δ_D_ = 15.5, δ_P_ = 10.4, δ_H_ = 7.0). The determined HSPs for ***m*** **-C**_**6**_ **PhCO-BTBT** (δ_D_ = 18.9 MPa^1/2^, δ_P_ = 5.7 MPa^1/2^, and δ_H_ = 5.8 MPa^1/2^) indicate that these green solvents have relatively small solute–solvent interaction distances (*R*_a_ = (4Δδ_D_^2^ + Δδ_P_^2^ + Δδ_H_^2^)^1/2^) of 2.4 MPa^1/2^, 4.4 MPa^1/2^, 6.4 MPa^1/2^, and 8.3 MPa^1/2^, respectively, which aligns with the observed solubility trend across these green solvents. On the other hand, ***m*** **-C**_**6**_ **PhCO-BTBT** exhibits a room temperature solubility of 0.24 mg·mL^–1^ in ethanol (δ_D_ = 15.8, δ_P_ = 8.8, δ_H_ = 19.4), which is consistent with its relatively larger *R*_a_ value of 15.3 MPa^1/2^. However, note that the semiconductor solution in ethanol processed at moderate temperatures (solubility = 1.5 mg·mL^–1^ at ∼70 °C) could still yield electroactive thin films via drop-casting (*vide infra*). Considering that ***m*** **-C**_**6**_ **PhCO-BTBT** does not employ a special oligo­(ethylene glycol) side chain or an ionic functional group, the observed solubilities in these five different polar green solvents, especially in ethyl acetate, acetone, and ethanol, are very attractive. When compared with highly toxic and environmentally hazardous chlorinated aliphatic/aromatic or nonchlorinated aromatic solvents that are commonly used to prepare semiconductor solutions, these solvents present much less of a health and environmental hazard, and they could potentially be sourced from renewable biomass.^50,51^

Describing an organic solvent as “green” is indeed a complex notion that needs to consider various factors, including health, safety, environmental impact, and sustainability, which sometimes lead to differing assessment approaches. The selected green solvents for studying ***m*** **-C**_**6**_ **PhCO-BTBT**-based transistor fabrication each have their own strengths and weaknesses in terms of greenness. Among them, ethoxybenzene demonstrates an overall high GlaxoSmithKline (GSK) greenness composite score (G) of 7.2.^4^ Here, it is crucial to note that this G value for ethoxybenzene could potentially be even higher, as it received a relatively conservative health score of 4.9, due to limited available information. Another solvent, 2-methyltetrahydrofuran, has a relatively lower G score of 4.4. However, it stands out as a biorenewable green solvent with a reduced life cycle footprint. This compound can be derived from corn cob waste (*eco*MeTHF) with a significantly lower carbon footprint (0.150 kg CO_2_ production per kg) compared to petrochemical feedstock-based tetrahydrofuran (THF) (5.46 kg CO_2_ production per kg).^52^ The other solvents, ethyl acetate, acetone, and ethanol have excellent health, environment, and safety subcategory scores of 7.1–8.9.^4^ In addition, all three of these solvents could be obtained from renewable, sustainable biobased materials, leading to a considerable reduction in their environmental impact.^53^ These solvents have been listed among the preferred or recommended eco-friendly green solvents for industrial applications including in varied medicinal chemistry laboratories.^50,51,54^ Especially, ethanol is widely regarded as the most ideal environmentally friendly solvent whenever water is not suitable for processing.^38^ To the best of our knowledge, there are very limited examples of small molecular OFETs in general,^53,55^ and no reported examples of a BTBT-based OFET fabricated using the green solvents we are interested in. Only recently, a few reports have demonstrated OFETs based on solution processing of BTBT-based semiconductors from different green solvents including anisole, cyclohexanone, and diethyl carbonate.^56,57^ The charge transport characteristics of the new mono­(aryl)­carbonyl functionalized BTBT molecule were explored in top-contact/bottom-gate (TC/BG) OFETs by spin-coating ***m*** **-C**_**6**_ **PhCO-BTBT** solutions (4.0 mg·mL^–1^) in 2-methyltetrahydrofuran, ethoxybenzene, ethyl acetate, and acetone, and by drop-casting ***m*** **-C**_**6**_ **PhCO-BTBT** solution in ethanol (1.5 mg·mL^–1^). The semiconducting thin films (∼40–45 nm) were deposited onto p^+2^-Si/SiO_2_ (300 nm)/PS-brush (*M*_n_ = 5 kDa) substrates, which has an ultrathin (∼3.6 nm), densely packed (grafting density ≈ 0.45 chains·nm^–2^), smooth (R_q_ = 0.17–0.18 nm for a 10 × 10 μm^2^ area) hydrophobic surface.^28^ This surface promotes the formation of an optimal thin-film microstructure for efficient charge transport. The microstructural and morphological characterizations were performed by atomic force microscopy (AFM) and out-of-plane θ-2θ X-ray diffraction (i.e., in Bragg–Brentano geometry) (Figure 6a,c). The electrical characterizations conducted under ambient conditions revealed that all transistors based on ***m*** **-C**_**6**_ **PhCO-BTBT**, processed from five different green solvents, exhibit clear *p*-channel semiconductor behavior with high current-modulation characteristics (Figures 6b, S17 and S18). The transistors function only under a negative gate bias. The absence of an *n*-channel transport is attributed to the high-lying LUMO energy level (−2.75 eV) and the relatively larger intramolecular reorganization energy for electron transport vs hole transport (λ_e_ = 310 meV > λ_h_ = 265 meV, see Supporting Information for calculation details).^58–60^ While poor hole mobilities (μ_h_) of ∼10^–4^ cm^2^/V·s were measured for the devices with only spin coated semiconductor layers, μ_h_ progressively increased to 0.001 cm^2^/V·s and 0.01 cm^2^/V·s with thermal annealing at 70 and 90 °C, respectively. The highest hole mobility for all green solutions was achieved with the semiconductor thin films annealed at 120 °C (for 20 min). The highly responsive semiconductor behavior of ***m*** **-C**_**6**_ **PhCO-BTBT** (Δμ_h_ ≈ 20,000×) to thermal annealing was evident with the morphological characterizations. As shown in Figure 6e, thermal annealing facilitates the progressive growth of smooth and micrometer-sized terraced 2D islands with sharp edges and well-defined steps, evolving from nanometer-sized (∼100–200 nm) small granular domains obtained immediately after the spin-coating process. Additionally, the crystallinity increases, and the molecular packing becomes denser (i.e., decrease in *d*-spacing) in the out-of-plane direction after thermal annealing (Figure 6c, top scans). It is worth noting that when the semiconductor thin films were annealed at a higher temperature (*T*_annealing_ = 130–140 °C), the transistor characteristics were found to considerably deteriorate (I_on_/I_off_ < 10). AFM analysis reveals that the semiconductor layer dewets from the PS surface and forms discontinuous micron-sized domains (∼70–80 nm thickness), which hinder the OFET semiconducting channel (Figure S19).^10^

**Figure 6:**
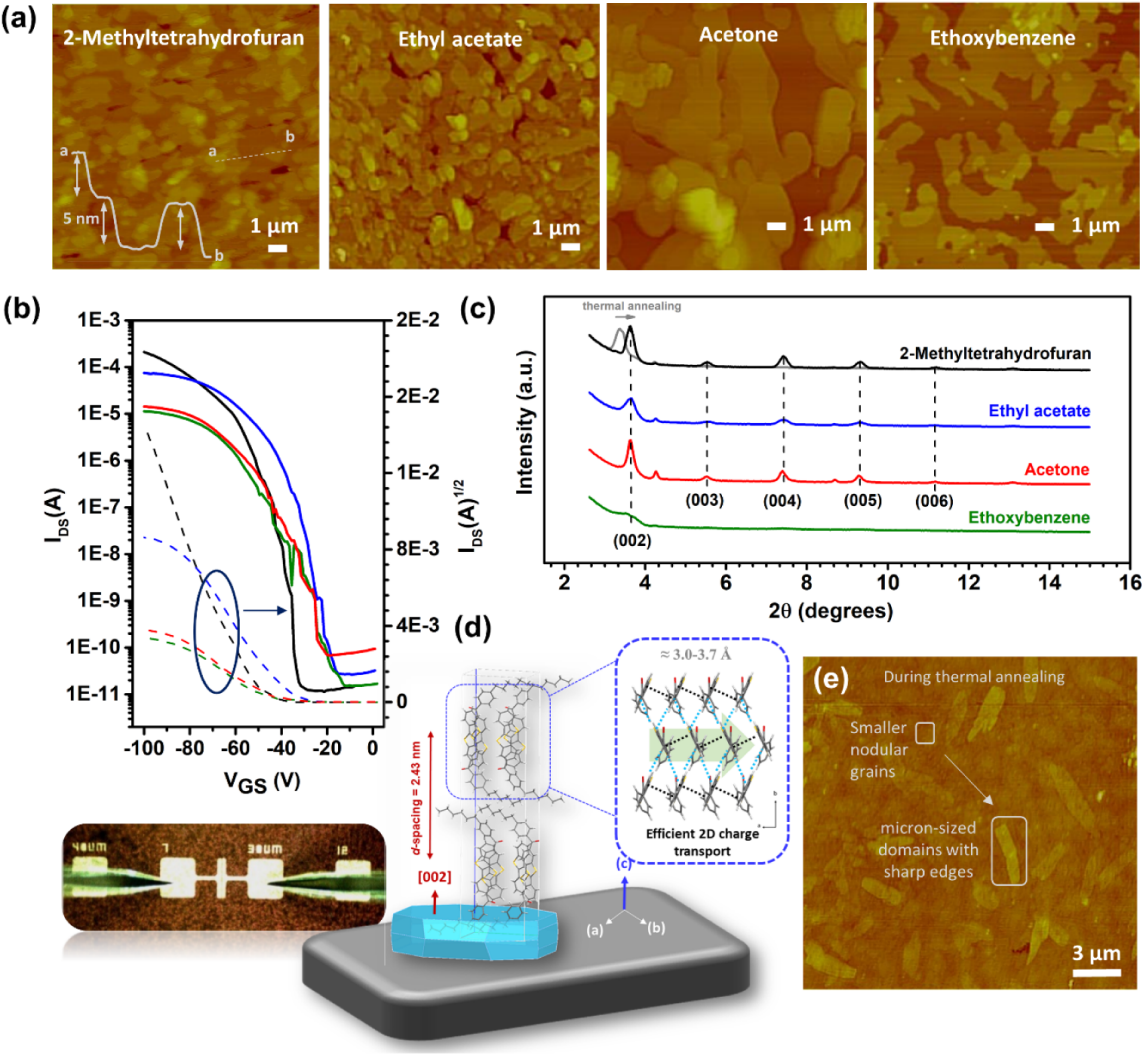
Tapping mode atomic force microscopy (AFM) topographic images (a) and θ-2θ out-of-plane X-ray diffraction (Bragg–Brentano configuration) scans with the assigned planes (c) for p^+2^-Si/SiO_2_ (300 nm)/PS-brush (*M*_n_ = 5 kDa)/***m*** **-C**_**6**_ **PhCO-BTBT** (40–50 nm) thin films that are spin-coated from four different green solvents (2-methyltetrahydrofuran, ethyl acetate, acetone, and ethoxybenzene) and annealed at 120 °C (for 20 min). (a) The measured step-height profile (∼2.5×*n* nm (*n (integer)* ≥ 1)) of the 2D terraced multilayer molecular islands. In the top XRD scan, the spin-coated sample without thermal annealing (gray solid line) is also provided for comparison. (b) Transfer (V_DS_ = −100 V) characteristics for p^+2^-Si/SiO_2_ (300 nm)/PS-brush (*M*_n_ = 5 kDa)/***m*** **-C**_**6**_ **PhCO-BTBT** (40–50 nm)/Au (50 nm) OFET devices processed from four different green solvents. The I_DS_^1/2^ vs V_G_ plots (dashed curves) are used for hole mobility calculations. The inset shows a representative top-view image of an OFET device during measurement. (d) The molecular arrangement in the out-of-plane [002] and in-plane (*ab*-plane) directions. (e) The morphological change from small nodular grains to micrometer-sized domains with sharp edges, as observed during the thermal annealing process for an unfinished sample.

The maximum saturation hole mobilities were achieved for thin films spin-coated from 2-methyltetrahydrofuran (μ_h_^max^ = 1.87 cm^2^/V·s, μ_h_^avg^ = 1.21 cm^2^/V·s (I_on_/I_off_ = 10^7^-10^8^)) and ethyl acetate (μ_h_^max^ = 0.62 cm^2^/V·s, μ_h_^avg^ = 0.41 cm^2^/V·s (I_on_/I_off_ = 10^6^-10^7^)) solutions (Figures 6b and S17). On the other hand, ***m*** **-C**_**6**_ **PhCO-BTBT** thin films spin-coated from acetone and ethoxybenzene showed relatively lower but still respectable hole mobilities of 0.07–0.11 cm^2^/V·s (I_on_/I_off_ = 10^5^-10^6^) (Figure 6b). As shown in Figure 6a, semiconductor thin films processed from all four green solvents exhibited terraced 2D islands that oriented along the substrate plane. Despite all being micron-sized grains, the grain sizes for 2-methyltetrahydrofuran and ethyl acetate were observed to be relatively smaller, yet they showed better intergrain connectivity across large distances (>30 μm). On the other hand, while ethoxybenzene and acetone appear to have larger domains, two different issues were identified with them. Ethoxybenzene thin-film domains exhibited very poor crystallinity (Figure 6(c)-green line), and acetone thin-film domains, despite being crystalline (Figure 6c, red line), were observed to be isolated from each other and showed poor intergrain connectivity (R_q_ = 30.25 nm for a 30 × 30 μm^2^ area, Figure S20). The thin films processed from 2-methyltetrahydrofuran, ethyl acetate, and acetone showed the best crystallinities with multiple high-intensity sharp peaks. As shown in Figure 6c, based on the low-angle diffraction peak observed at 2θ = 3.63°, the *d*-spacing value is calculated as 2.43 nm. This peak matches perfectly with the (002) crystal plane simulated based on the single-crystal structure and corresponds to the half of the unit cell parameter along the *c*-axis. This unit cell arrangement in the thin-film phase indicates a highly favorable edge-on oriented crystallization behavior in the out-of-plane direction (Figure 6d). The higher-order diffraction peaks ((003)-(006)) were also evident in the same XRD spectra, confirming the presence of a long-range crystal ordering in the out-of-plane direction. As a result, a very favorable 2D herringbone packing motif (multiple π-interactions at 3.1–3.7 Å, *vide supra*) becomes effective along the *ab*-plane, including face-to-face π-π interactions along the *a*-axis. This is crucial for efficient charge transport in semiconductor thin films from source to drain.^58^ The *d*-spacing value matches with the measured step-height profiles (∼2.5×*n* nm (*n (integer)* ≥ 1)) of the 2D terraced multilayer molecular islands (Figure 6a inset). Using the Scherrer equation and taking the first three diffraction peaks into consideration,^61^ the (002) coherence lengths (L) are estimated to be ∼34–36 nm for 2-methyltetrahydrofuran, ∼27–29 nm for ethyl acetate, and ∼40–45 nm for acetone (see Figure S21 for Gaussian fittings). The extents of coherence lengths for all solvents are on the scale of the thin-film thickness, which suggests an excellent degree of molecular ordering in the out-of-plane direction. On the other hand, the absence of any broad peaks at 2θ ≈ 20–25° in the out-of-plane direction, which typically corresponds to π-interactions, suggests that all π-interactions are mainly along the thin-film plane, which is important for charge transport in molecular semiconductors.^58^

High hole mobility and suitable microstructures/morphologies are achieved when the semiconductor is processed using 2-methyltetrahydrofuran and ethyl acetate, which have moderate *R*_a_ values of 4.4 and 6.4 MPa^1/2^, respectively, along with reasonable solvent boiling points of 77–78 °C. When *R*_a_ decreases in ethoxybenzene (2.4 MPa^1/2^) or increases in acetone (8.3 MPa^1/2^), both mobility and the microstructure/morphology deteriorate. In ethoxybenzene, with its relatively low *R*_a_, poor crystallinity was observed due to a low crystallization tendency. Conversely, in acetone, with its relatively high *R*_a_, rapid crystallization occurred, resulting in a poorly connected domain formation. It is also worth noting that the low boiling point of acetone (56 °C) and the high boiling point of ethoxybenzene (169 °C) may further influence the observed crystallization behaviors. These results suggest that an optimal solute–solvent interaction distance (*R*_a_) is the key to a proper thin-film self-assembly process. In other words, as the solvent evaporates during spin-coating, molecules should not immediately precipitate or crystallize due to a large *R*_a_ value. Instead, they should gradually saturate, leading to a more controlled thin-film crystallization and molecular self-assembly process.^62,63^ On the other hand, semiconductor thin films drop-casted from ethanol, which has a much larger *R*_a_ value of 15.3 MPa^1/2^, yielded OFETs with a lower μ_h_ of ∼0.001 cm^2^/V·s (I_on_/I_off_ ≈ 10^4^ and V_th_ = −39 V) (Figures S18). The excellent thin-film crystallization and morphological behaviors observed with 2-methyltetrahydrofuran solution align with our recent findings using an ambient stable *n*-type small molecule.^27^

To the best of our knowledge, there are very limited examples of OFETs processed from some of these solvents, especially from ethyl acetate, acetone, and ethanol.^53,55^ Additionally, this is the first time that a BTBT-based OFET has been fabricated from these green solvents. The excellent *p*-channel performances, having high μ_h_s and I_on_/I_off_ ratios, achieved with 2-methyltetrahydrofuran and ethyl acetate green solvents, despite the absence of any meticulous process optimization, rank among the highest reported for a green solvent.^56^ Here, it is noteworthy that the V_th_ values for these OFETs typically fall within the range of −30 V to −35 V. Therefore, considering the ideal linear characteristics of the “I_DS_^1/2^ *vs.* V_GS_” transfer curves of the green solvent-processed OFETs, yet with noticeable threshold voltages, our saturation hole mobilities could be further evaluated by applying a reliability factor (*r*_sat_), as described in recent experimental/modeling studies.^64,65^ The reliability factor is used to define the equivalent electrical performance of an OFET device with a near-zero threshold voltage, following the ideal Shockley equations.^65^ This procedure shows that *r*_sat_s for our OFETs are 32–38%, yielding μ_eff_s of 0.17–0.35 cm^2^/V·s for the equivalent ideal transistor according to the Shockley FET equations (i.e., a linear transfer characteristics with V_th_ = 0 V) (see Supporting Information for details, Figure S22). Among all known high-performance *p*-type semiconductors, ***m*** **-C**_**6**_ **PhCO-BTBT** exhibits one of the deepest HOMO energy levels ever recorded at −6.04 eV.^6^ The large V_th_s for the current OFETs are undoubtedly the result of ***m*** **-C**_**6**_ **PhCO-BTBT**s extremely low-lying HOMO energy level and unoptimized metal–semiconductor interface, as well as the existence of possible trap states.^66,67^ Further optimizations with regard to the metal–semiconductor interface and semiconducting thin film could significantly reduce the observed threshold voltage and further enhance the transistor performance.

## Conclusions

In summary, we have successfully demonstrated a novel asymmetric mono­(aryl)­carbonyl functionalization approach to synthesize a new, highly soluble [1]­benzothieno­[3,2-*b*]­[1]­benzothiophene (BTBT) semiconductor, ***m*** **-C**_**6**_ **PhCO-BTBT**. The new semiconductor was synthesized on a gram scale via a two-step, transition-metal-free synthesis, followed by comprehensive structural, physicochemical, (opto)­electronic, and solubility characterizations, along with theoretical calculations. An asymmetric π-electronic structure with significant ground-state (μ_g_ = 3.17 D) and excited-state (μ_e_ = 12.69 D) dipole moments was revealed together with a high degree of polarizability. We have achieved an unprecedented solubility of 176.0 mg·mL^– 1^ (0.41 M), which, to our knowledge, represents the highest room-temperature solubility ever reported for a high-performance organic semiconductor. The single-crystal structural analysis reveals a herringbone motif with multiple edge-to-face interactions and nonclassical hydrogen bonds involving the carbonyl unit. The antiparallel arrangement of the asymmetric backbones also facilitates face-to-face π-π interactions. Hansen solubility parameters (HSP) analysis of ***m*** **-C**_**6**_ **PhCO-BTBT**, combined with two novel comparative molecules, ***m*** **-PhCO-BTBT**/***m*** **-C**_**7**_ **CO-BTBT**, and prior BTBTs from the literature, has revealed distinctive relationships between molecular structure, specific interaction energy density, cohesive energetics, and solubility. Compared to nonfunctionalized “π-π-σ” or dicarbonyl-functionalized “(π/σ)-OC-π-CO-(π/σ)” molecular architectures, the asymmetric monocarbonyl insertion (i.e., “π-CO-π-σ”) achieves exceptional solubility by tuning specific interaction energy densities (δ_D_^2^, δ_P_^2^, δ_H_^2^) to minimize the solute–solvent interaction distances (R_a_) and optimize cohesive energetics. Among monocarbonyl BTBTs, alkyl-aryl substitution offers a key design advantage over alkyl or aryl substitution alone, demonstrated through better HSPs, particularly dispersion interactions. Using Scatchard–Hildebrand regular solution theory, thermodynamic correlations in the HSP space between solubility and interaction distances were established for all three new compounds across 28 solvents, revealing strong connections to physicochemical properties. To our knowledge, this represents the first integration of Hansen-based solubility study and thermal characterization in the literature, potentially paving the way for new fundamental and applied research in organic semiconductor solubility.

This asymmetrically functionalized BTBT π-structure enables semiconductor formulations in environmentally friendly green solvents, such as 2-methyltetrahydrofuran, ethyl acetate, ethoxybenzene, acetone, and ethanol, achieving solubilities of up to ∼12.5 mg·mL^–1^ (25 °C), thanks to low *R*_a_ values in the HSP space. This has enabled us to fabricate top-contact/bottom-gate (TC/BG) OFETs using these green solvents. To the best of our knowledge, this work represents the first instance of BTBT-based semiconducting thin films produced from these green solvents. High hole mobilities of up to 1.87 cm^2^/V·s (μ_eff_ ≈ 0.4 cm^2^/V·s) were achieved, with I_on_/I_off_ ratios of 10^7^-10^8^, in properly microstructured thin films exhibiting well-connected, 2D plate-like crystalline domains with edge-on oriented molecules. Solute-green solvent interaction distances in the HSP space were found to critically influence the microstructural and morphological properties. ***m*** **-C**_**6**_ **PhCO-BTBT** is now a rare high-mobility *p*-type semiconductor processed from these green solvents, with a notably deep HOMO level of −6.04 eV. Transistor performance from green solvents could be further enhanced by expanding device strategies with additional solvents or mixtures, adopting varied OFET architectures, and optimizing metal–semiconductor interfaces (to lower threshold voltage) and semiconducting thin films.

This asymmetric mono­(aryl)­carbonyl functionalization approach and our findings in this study provide unique insights and key information into the design and development of green solvent-processable π-conjugated molecules and could open new directions in the field of green optoelectronics, biosensors, and bioelectronics. We envision that novel semiconductor design perspectives for future solubility approaches and green solvent processing could emerge, and it could be extended to other intrinsically insoluble DAcTTs and (hetero)­acenes. Furthermore, ***m*** **-C**_**6**_ **PhCO-BTBT**s strong solubility in polar (a)­protic solvents (e.g., ethanol, ethyl acetate, and acetone), which are typically orthogonal to most π-conjugated material, along with its wide optical band gap, deep HOMO, excellent green processing, and efficient hole transport properties, makes this new molecular π-framework highly appealing for multilayer (opto)­electronic devices. Our findings that monobenzoyl functionalization deactivates the BTBT π-core toward further functionalization eliminates purification challenges and simplifies the synthesis of new mono­(aryl)­carbonyl-BTBT molecules in future exploratory synthesis research. Further studies on green-OFET devices and molecular engineering of fused π-architectures by tailoring our current approach are currently underway in our laboratory.

## Experimental Section

### Materials and Methods

Friedel–Crafts acylation reactions were conducted under ambient conditions, and all reagents were used as received without any purifications. Chromatographic purification was carried out using 230–400 mesh silica gel. ^1^H and ^13^C NMR spectroscopy characterizations were carried out on a Bruker 400 spectrometer (^1^H, 400 MHz; ^13^C, 100 MHz). Elemental analyses were recorded on a Thermo Scientific FLASH 2000 instrument. Atmospheric pressure chemical ionization mass spectra (APCI-MS) were recorded by using a molecular solid sample on an Advion-Expression^L^-CMS instrument. Thermogravimmetric analysis (TGA) and differential scanning calorimetry (DSC) measurements were performed under nitrogen (heating rate ≈ 10 °C/min) using Mettler Toledo-TGA/STDA 851 and Mettler Toledo-DSC 822e model instruments, respectively. For the calibration process in DSC, In and Zn standards (Mettler Toledo, Schwerzenbach, Switzerland) were used. Conventional melting temperatures were recorded on Electrothermal IA9000 instrument. Cyclic voltammograms were recorded on the BAS-Epsilon potentiostat/galvanostat system (Bioanalytical Systems Inc. (Lafayette, IN) equipped with a C3-cell stand electrochemical station, using a ferrocene/ferrocenium redox couple (Fc/Fc^+^: *E*_1/2_ = +0.40 V) as the internal standard. Pt wire was used as the working and the counter electrodes, and the reference electrode was Ag/AgCl (3 M NaCl). The UV–vis absorption studies were performed on a Shimadzu UV-1800 spectrophotometer, and photoluminescence (PL) spectra were recorded with an Agilent-Cary Eclipse fluorescence spectrophotometer. Density Functional Theory (DFT) calculations of equilibrium geometry, electrostatic potential map, frontier molecular orbitals, molecular dipole moment, polarizability, and single-point energy were performed using the B3LYP functional and 6–31G** basis set as implemented in Spartan’24 version 1.0.0 (Wave function Inc., 2024, Irvine, CA).^68^ The theoretical UV −Vis spectrum was calculated via the TD-DFT method using the B3LYP functional and 6–31G** basis set. The intramolecular reorganization energies for hole (λ_h_) and electron (λ_e_) transport were calculated using the standard procedure described in the literature (see Supporting Information for details).^59^ Data collection for single crystals was carried out using a Bruker APEXII CCD diffractometer (λ = 0.71073 Å). The X-ray crystallographic coordinates for ***m-C***_***6***_ ***PhCO-BTBT*** are deposited at the Cambridge Crystallographic Data Centre (CCDC) under the deposition number 2451055. These data can be obtained free of charge from the Cambridge Crystallographic Data Center via www.ccdc.cam.ac.uk/data_request/cif. Hirshfeld surface and fingerprint plots were produced with the CrystalExplorer21 software.^69^ Intermolecular interaction energies were calculated using the DFT method and the 6–31G­(d,p) basis set with CE-B3LYP model energies, as implemented in CrystalExplorer21.^70^ The atomic coordinates for these calculations were derived from crystallographic data.

### Synthesis and Characterization

[1]­Benzothieno­[3,2-*b*]­[1]­benzothiophene (**BTBT**), 2,7-dioctyl[1]­benzothieno­[3,2-*b*]­[1]­benzothiophene (**C**_***8***_ **-BTBT**), and 1,1’-(benzo­[*b*]­benzo­[4,5]­thieno­[2,3-*d*]­thiophene-2,7-diyl)­bis­(octan-1-one) **(D­(C**_***7***_ **CO)-BTBT)** were synthesized according to the reported procedures.^10,13^

#### Synthesis of benzo­[*b*]­benzo­[4,5]­thieno­[2,3-d]­thiophen-2-yl­(4-hexylphenyl)­methanone (**m-C**_***6***_ **PhCO-BTBT**)

AlCl_3_ (2.42 g, 18.14 mmol) was added into a solution of [1]­benzothieno­[3,2-*b*]­[1]­benzothiophene (BTBT) (0.80 g, 3.32 mmol, 1.0 equiv) in anhydrous dichloromethane (70 mL) at −10 °C. The resulting solution was stirred at −10 °C for 30 min, and then it was cooled down to −78 °C. 4-Hexylbenzoyl chloride (3.74 g, 16.64 mmol, 5.0 equiv) was subsequently added dropwise, and the mixture was stirred for 1 h at the same temperature. The reaction mixture was allowed to warm to room temperature and stirred for 2 days. After extraction of the reaction mixture with CHCl_3_, the organic layer was dried with Na_2_SO_4_, filtered, and concentrated to yield the crude product. The crude was then purified by column chromatography on silica gel using chloroform as the mobile phase to afford the final product as an off-white solid (1.21 g, 84.9% yield). Melting point: 151–152 °C; ^1^H NMR (400 MHz, CDCl_3_), δ (ppm): 8.40 (s, 1H), 7.99–7.93 (m, 4H), 7.81 (d, 2H, *J* = 8.0 Hz), 7.51–7.45 (m, 2H), 7.35 (d, 2H, *J* = 8.0 Hz), 2.73 (t, 2H, *J* = 8.0 Hz), 1.69 (m, 2H), 1.35 (m, 6H), 0.91 (m, 3H, *J* = 8.0 Hz); ^13^C NMR (100 MHz, CDCl_3_), δ (ppm): 195.8, 148.2, 142.8, 141.9, 136.7, 135.9, 135.3, 134.4, 133.0, 132.8, 130.3, 128.4, 126.8, 126.5, 125.8, 125.2, 124.1, 122.1, 121.2, 36.1, 31.8, 31.3, 28.8, 22.7, 14.1; MS (APCI) *m*/*z* calcd for C_27_H_24_OS_2_: 428.13 [M^+^]; found: 428.440 [M^+^]; elemental analysis calcd (%) for C_27_H_24_OS_2_: C, 75.66; H, 5.64; found: C, 75.63; H, 5.70.

#### Synthesis of benzo­[*b*]­benzo­[4,5]­thieno­[2,3-d]­thiophen-2-yl­(phenyl)­methanone (**m-PhCO-BTBT**)

AlCl_3_ (0.81 g, 6.09 mmol) was added into a solution of [1]­benzothieno­[3,2-*b*]­[1]­benzothiophene (BTBT) (1.20 g, 4.98 mmol, 1.0 equiv) in anhydrous dichloromethane (100 mL) at −10 °C. The resulting solution was stirred at −10 °C for 30 min, and then it was cooled down to −78 °C. Benzoyl chloride (3.51 g, 24.96 mmol, 5.0 equiv) was subsequently added dropwise, and the mixture was stirred for 1 h at the same temperature. The reaction mixture was allowed to warm to room temperature and stirred for 2 days. After extraction of the reaction mixture with CHCl_3_, organic layer was dried with Na_2_SO_4_, filtered, and concentrated to yield the crude product. The crude product was then purified by column chromatography on silica gel using chloroform as the mobile phase to afford the final product as an off-white solid (1.22 g, 71.2% yield). Melting point: 224–225 °C; ^1^H NMR (400 MHz, CDCl_3_), δ (ppm): 8.41 (s, 1H), 7.97–7.93 (m, 4H), 7.87 (d, 2H, *J* = 6.0 Hz), 7.65 (t, 1H, J = 6.0 Hz), 7.56–7.46 (m, 4H); ^13^C NMR (100 MHz, CDCl_3_), δ (ppm): 196.4, 142.9, 141.9, 137.9, 136.9, 136.1, 134.0, 133.1, 132.8, 132.4, 130.1, 128.5, 126.9, 126.8, 125.9, 125.2, 124.2, 122.2, 121.2; MS (APCI) *m*/*z* calcd for C_21_H_12_OS_2_: 344.03 [M^+^]; found: 344.540 [M^+^]; elemental analysis calcd (%) for C_21_H_12_OS_2_: C, 73.23; H, 3.51; found: C, 73.42; H, 3.73.

#### Synthesis of 1-(benzo­[*b*]­benzo­[4,5]­thieno­[2,3-d]­thiophen-2-yl)­octan-1-one (**m-C**_***7***_ **CO-BTBT**)

AlCl_3_ (0.62 g, 4.66 mmol) was added into a solution of [1]­benzothieno­[3,2-*b*]­[1]­benzothiophene (BTBT) (1.12 g, 4.66 mmol, 1.0 equiv) in anhydrous dichloromethane (80 mL) at −10 °C. The resulting solution was stirred at −10 °C for 30 min, and then it was cooled down to −78 °C. Octanoyl chloride (0.76 g, 4.66 mmol, 1.0 equiv) was subsequently added dropwise, and the mixture was stirred for 1 h at the same temperature. The reaction mixture was allowed to warm to room temperature and stirred for 2 days. After extraction of the reaction mixture with CHCl_3_, organic layer was dried with Na_2_SO_4_, filtered, and concentrated to yield the crude product. The crude was then purified by column chromatography on silica gel using chloroform as the mobile phase to afford the final product as an off-white solid (1.24 g, 72.6% yield). Melting point: 179–180 °C; ^1^H NMR (400 MHz, CDCl_3_), δ (ppm): 8.56 (s, 1H), 8.06 (d, 1H, *J* = 8.0 Hz), 7.97–7.95 (m, 3H), 7.51–7.46 (m, 2H), 3.08 (t, 2H, *J* = 8.0 Hz), 1.82–1.79 (m, 2H), 1.40–1.32 (m, 8H), 0.92 (t, 3H, *J* = 8.0 Hz); ^13^C NMR (100 MHz, CDCl_3_), δ (ppm): 199.7, 142.8, 142.2, 136.8, 136.2, 133.7, 133.0, 132.8, 125.8, 125.1, 124.8, 124.5, 124.2, 122.1, 121.4, 38.8, 31.4, 29.4, 28.7, 24.6, 22.6, 14.1; MS (APCI) *m*/*z* calcd for C_22_H_22_OS_2_: 366.11 [M^+^]; found: 366.469 [M^+^]; elemental analysis calcd (%) for C_22_H_22_OS_2_: C, 72.09; H, 6.05; found: C, 72.33; H, 6.14.

### Solubility Measurements and HSP Analysis

UV–vis absorption spectroscopy method is used in order to determine the semiconductor solubility in different solvents. Initially, a linear calibration curve was generated (*R* ^2^ = 0.995–0.997) at the semiconductor’s absorption peak (λ_max_ = 328 nm for ***m*** **-C**_**6**_ **PhCO-BTBT**, 330 nm for ***m*** **-PhCO-BTBT**, and 329 nm for ***m*** **-C**_**7**_ **CO-BTBT**) in chloroform. This was achieved by recording the UV–vis absorption spectra of the semiconductor standard solutions (ranging from 9.3 × 10^–7^ M to 5.8 × 10^–5^ M). Subsequently, saturated solutions of the semiconductor were prepared by weighing approximately 10.0 mg of the organic semiconductor solid into a vial and adding 500 μL of a specific solvent using a micropipette. After preparation, the mixture was stirred and sonicated for 10 min at room temperature. Subsequently, the mixture was filtered through a PTFE syringe filter with a pore size of 0.20 μm (VWR, part of Avantor). The filtrate was diluted (up to 100–5000 times depending on the specific solvent) with chloroform to bring the optical absorption within the range of the calibration curve. For each solvent, the absorbance at the absorption peak was measured, and the corresponding solubility value was calculated by substituting this absorbance value into the Beer–Lambert law equation (A = ε·b·c), where A is the absorbance, ε is the molar absorptivity, b is the path length, and c is the concentration. For solubilities exceeding 10.0 mg·mL^–1^, an additional gravimetric method is employed. In this method, approximately 10.0–15.0 mg of the semiconductor solid is accurately weighed in a vial. Incremental volumes of the solvent (in 50–100 μL portions) are then added using a micropipette. After each addition, the solution is stirred or sonicated for 10 min at room temperature. This process continues until complete dissolution is visually confirmed. Once dissolution is complete, the semiconductor solution is filtered through a PTFE syringe filter (VWR, part of Avantor, 0.20 μm pore size) and then evaporated to dryness by using a rotary evaporator. The gravimetric solubility was determined by calculating the ratio of the recovered semiconductor solid weight (*m*_osc_) to the total amount of solvent (*V*_solvent_), using the equation “solubility = *m*_osc_/*V*_solvent_.” Typically, the difference in solubilities determined via spectroscopic and gravimetric methods was found to be less than 4%. The Hansen solubility sphere parameters and fitting accuracy were determined using the Genetic algorithm in the HSPiP Program (5^*th*^ *Edition Version 5.4.08*), with a solubility limit of 4.0 mg·mL^–1^.^29^ Solubility scores of “1” (indicating a good solvent) and “0” (indicating a nonsolvent) were assigned based on the solubility tests described above. The group contribution methodology was employed using Neural Network techniques in the same HSPiP program.

### OFET Device Fabrication and Electrical Characterization

Top-contact/bottom-gate (TC/BG) organic field-effect transistors (OFETs) were fabricated on a heavily *p*-doped (100) silicon substrate (p^++^-Si) having a 300 nm thermally grown silicon dioxide (SiO_2_) as the gate dielectric layer. The substrates were cleaned via sonication in an ultrasonic bath with hexane, acetone, and ethanol, respectively, for 10 min each, dried with nitrogen and treated with air plasma for 3 min (Harrick Plasma, 30W). An ultrathin (∼3.6 nm) polystyrene brush (PS-brush) layer (grafting density ≈ 0.45 chains·nm^–2^) was formed on the p^+2^-Si/SiO_2_ (300 nm) substrates using hydroxyl-terminated polystyrene (*M*_*n*_ = 5.0 kDa, *M*_*w*_ */M*_*n*_ *= 1.05, Polymer Source Inc.*) via a “grafting-to” method. Organic semiconductor films (40–50 nm thick) were spin-coated onto p^+2^-Si/SiO_2_ (300 nm)/PS-brush (*M*_n_ = 5 kDa) substrates from green solvent solutions (4 mg·mL^–1^ in 2-methyltetrahydrofuran, ethyl acetate, ethoxybenzene, and acetone) at 1200 rpm for 60 s. For deposition from ethanol (1.5 mg·mL^–1^), the drop-casting method was used at solution and substrate temperatures of 70 °C. The semiconductor thin films were then annealed at varied temperatures (90–140 °C) under vacuum (≈0.01 Torr). The surface morphology and the microstructure of the solution-processed semiconductor thin films were characterized using atomic force microscopy (NanoSurf FlexAFM C3000) and X-ray diffraction (Malvern Panalytical Empyrean diffractometer) techniques. Finally, Au source-drain electrodes (50 nm thickness) with variable channel lengths of 30, 40, 50, 60, and 80 μm (width = 1000 μm) were deposited via thermal evaporation (growth rate = 0.2–0.3 Å/s) under high vacuum (∼10^–6^ Torr) using high-density deposition masks (Ossila, E322). The electrical characterizations of the p^++^-Si/SiO_2_ (300 nm)/PS-brush (*M*_n_ = 5 kDa)/***m*** **-C**_**6**_ **PhCO-BTBT** (40–45 nm)/Au (50 nm) OFET devices were performed in an ambient probe station (Everbeing BD-6) using a Keithley 2614B source-measure unit (without excluding natural or fluorescent lighting). Charge carrier mobility (μ_h_) was estimated in the saturation regime from the I_DS_^1/2^ vs V_GS_ transfer plots based on the conventional metal-oxide-semiconductor field-effect transistor (MOSFET) model using the formula, μ_sat_ = (2*I*_DS_ *L*)/[*WC*_i_(V_GS_ – V_th_),^2^ where I_DS_ is the source–drain current, *L* is the channel length, *W* is the channel width, C_i_ is the areal capacitance of the gate dielectric with the PS-brush layer (10.4 nF/cm^2^ based on our large grafting density of 0.45 chains·nm^–2^ and *M*_n_ = 5 kDa),^28^ V_GS_ is the gate voltage, and V_th_ is the threshold voltage. The μ_h_^avg^ values are based on the averages of at least 10 different OFET devices.

## Supplementary Material





## Notes

Previously reported green solvent processed OFETs and BTBT-based semiconductor characteristics (Tables S1 and S2); ^1^H/^13^C NMR and APCI-MS spectra (Figures S1–S9); TGA/DSC plots (Figures S10 and S11); absorption calibration curves (Figure S12); thermal-based thermodynamic characteristics (Table S3); calculated HSPs via theory (Figure S13); DFT-calculated molecular properties (Figure S14); solvatochromic characteristics (Table S4); DFT-calculated pairwise intermolecular interaction energies (Figure S15); photophysical characteristics for BTBT (Figure S16); computed excited state transition properties (Table S5); OFET characteristics (Figures S17 and S18); atomic force microscopy images (Figures S19 and S20); gauss fittings for the XRD spectra (Figure S21); reliability factors and effective mobilities (Figure S22)­(PDF)
Crstallographic data­(CIF)
