# Supplementary material for: Achieving Extreme Solubility and Green Solvent-Processed Organic Field-Effect Transistors: A Viable Asymmetric Functionalization of [1]Benzothieno[3,2‑b][1]benzothiophenes
Source: ACS Appl Mater Interfaces. 2025 Aug 22;17(35):49720–36. doi: 10.1021/acsami.5c12618 (PMC12412106; doi:10.1021/acsami.5c12618)
Supplement: Supplementary file 1 [file am5c12618_si_001.pdf]

SUPPORTING INFORMATION

Achieving Extreme Solubility and Green Solvent-Processed Organic Field-Effect Transistors: A Viable Asymmetric Functionalization of [1]Benzothieno[3,2-*b*][1]Benzothiophenes

Tevhide Ayça Yıldız, İbrahim Deneme, Hakan Usta*

Department of Materials Science and Nanotechnology Engineering, Abdullah Gül University, 38080 Kayseri, Türkiye.

*Address correspondence to: hakan.usta@agu.edu.tr

Table S1. The molecular structures, OFET device configurations/performances, semiconductor thin-film deposition methods, and the green and non-chlorinated solvents employed during deposition for previously reported solution-processable *n*-type and *p*-type semiconductors. The abbreviated names for each molecular structure are presented as indicated in their respective references.

Molecular Structure	Solution-based deposition method	Green Solvent	OFET Performance I_{on}/I_{off}, $\mu(cm^2/V\cdot s)$, $V_{th}(V)$	OFET Device Configuration	OFET Character. Environment	References
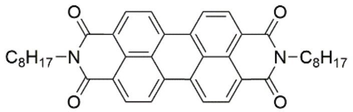 **PTCDI-C_8**	Solution-shearing	Anisole	1.5×10^7,0.13, 36 (*n*-channel)	Si/SiO_2/PS-brush/**PTCDI-C_8**/Au	Vacuum	*Ho et al.*[1]
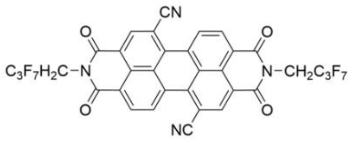 **PDIF-CN_2**	Solution-shearing	Purasolv EHL	2.3×10^3, 0.069, -32 (*n*-channel)	Si/SiO_2/PS-brush/**PDIF-CN_2**/Au	Vacuum	
		Methyl laurate	1.3×10^2, 0.000094, -52 (*n*-channel)			
		n-Amyl acetate	3.4×10^2, 0.000010, -52 (*n*-channel)			

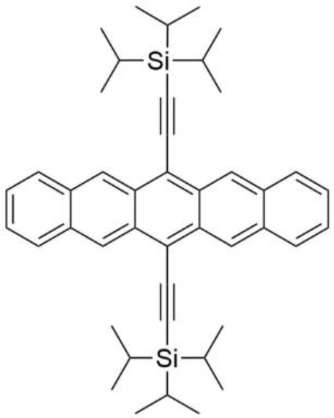 **TIPS-PEN**	Solution-shearing	t-Amyl methyl ether	5.1×10^{4}, 1.40, -32 (*p*-channel)	Si/SiO_2/PS-brush/**TIPS-PEN**/Au	Vacuum	*Ho et al.*[1]
		Anisole	4.4×10^{5},1.90, -33 (*p*-channel)			
		Isopropyl acetate	2.2×10^{4},0.67, -33 (*p*-channel)			
		Isobutyl acetate	3.7×10^{4}, 2.60, -29 (*p*-channel)			
		Dimethyl Carbonate	4.6×10^{4}, 1.30, -25 (*p*-channel)			
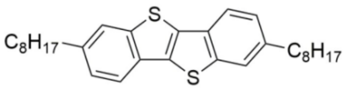 **C_8-BTBT**	Solution-shearing	t-Amyl methyl ether	$3.5\times10^{8,}$ 0.73, -30 (*p*-channel)	Si/SiO_2/PS-brush/**C_8-BTBT** /Au	Vacuum	
		Isobutyl acetate	4.6×10^{6}, 0.057, -31 (*p*-channel)			
		Isopropyl acetate	2.0×10^{8}, 0.92, -56 (*p*-channel)			
		Dimethyl Carbonate	2.3×10^{6}, 0.031, -19 (*p*-channel)			
		Anisole	1.1×10^{7}, 0.92, -38 (*p*-channel)			

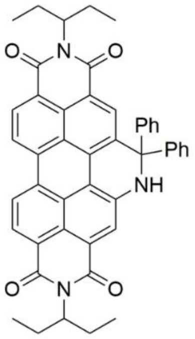<br>**X1**	Spin-coating	Propanol/n-butylamine	NA, 10^{-5},10 (*n*-channel)	Si/SiO_2/OTCS/**X1**/Au	Vacuum	Harris *et al.*[2]
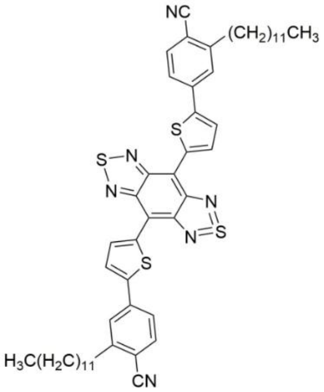<br>**TU-3**	Solution-shearing	Chlorobenzene/diethl succinate	6.43×10^4-2.84×10^5, 0.13-0.33, 0.2-4 (*n*-channel)	Si/SiO_2/PS-brush/**TU-3**/Au	Vacuum	Lee *et al.* [3]

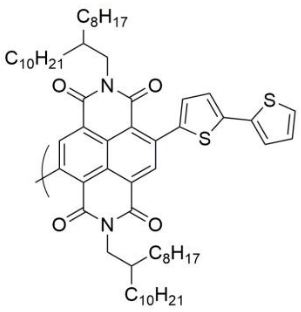 **P(NDI2OD-T2)**	Spin-coating	Chloroform/2-methyl tetrahydro furan	1.05-1.25×10^4, 0.086-0.11, 1.6-1.9 (*n*-channel)	Si/SiO_2/PS-brush/**P(NDI2OD-T2)**/Au	Vacuum	Lee *et al.* [3]
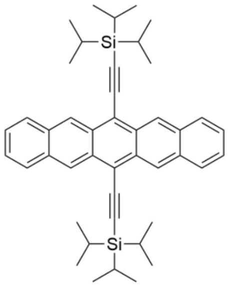 **TIPS-pentacene**	Solution-shearing	Toluene/ n-amyl acetate	1.67-3.31×10^7, 1.71-2.61, -23-(-16) (*p*-channel)	Si/SiO_2/PS-brush/**TIPS-pentacene**/Au	Vacuum	
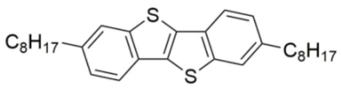 **C_8-BTBT**	Solution-shearing	Chloroform/2-methyl tetrahydro furan	2.85-9.81×10^7, 1.84-2.10, -30 (*p*-channel)	Si/SiO_2/PS-brush/**C_8-BTBT**/Au	Vacuum	

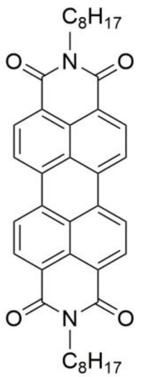 **PTCDI-C_8**	Solution-shearing	Anisole	(1.5 ± 0.7)×10^5, 0.31, 29 (*n*-channel)	Si/SiO_2/Shellac/**PTCDI-C_8**/Au	Vacuum	Lee *et al.* [4]
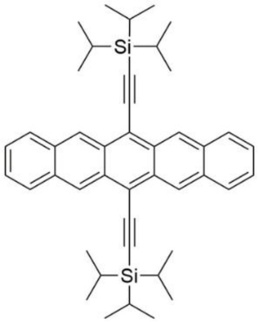 **TIPS-pentacene**	Solution-shearing	Tert-amyl methyl ether	(7.0±3.5)×10^6, 0.61, -31±2.3 (*p*-channel)	Si/SiO_2/Shellac/**TIPS-pentacene**/Au	Vacuum	
		Isobutyl acetate	(19±9.1)×10^6, 1.52, -30±3.8 (*p*-channel)			
		Anisole	(4.0±2.5)×10^6, 1.11, -25±8.0 (*p*-channel)			

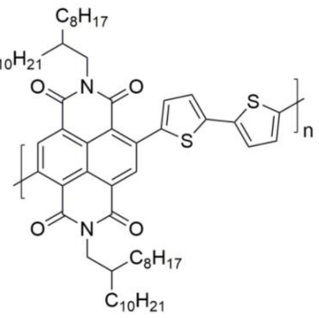<br>**P(NDI2OD-T2)**	Solution-shearing	Propylene glycol methyl ether acetate / 1,2- dichlorobenzene	NA, 0.28-1.03, 26.8-31.8 (*n*-channel)	Glass/Au-Ni/**P(NDI2OD-T2)**/PMMA/Al	Under N_2	Opoku *et al.* [5]
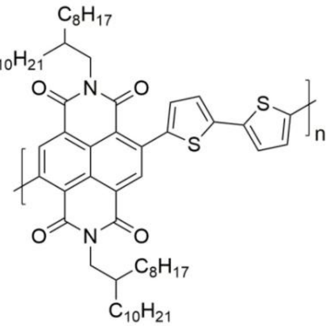<br>**P(NDI2OD-T2)**	Solution-shearing	Mesitylene/ acetophenone	$1.23\text{-}2.83\times10^3$, 0.448-0.574, 19.8-22.1 (*n*-channel)	Glass/Au-Ni/**P(NDI2OD-T2)**/ PMMA/Al	Under N_2	Opoku *et al.* [6]

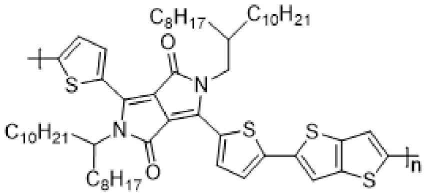<br>**DPPT-TT**	Solution-shearing	Mesitylene/ acetophenone	7.25×10^{1}-1.18×10^{2}, 0.445-0.634, -34.9-(-32.5) (*p*-channel)	Glass/Au-Ni/ **DPPT-TT**/PMMA/Al	Under N_2	Opoku *et al.* [6]
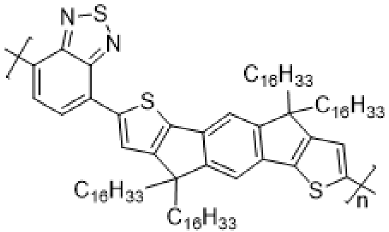<br>**IDT-BT**	Solution-shearing	Mesitylene/ acetophenone	1.46×10^{2}-5.59×10^{3}, 0.131-0.785, -26.7-(-18.8) (*p*-channel)	Glass/Au-Ni/ **IDT-BT** /PMMA/Al	Under N_2	

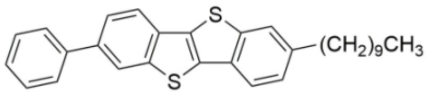<br>**Ph-BTBT-C$_{10}$**	Solution-shearing	Anisole	10^4-10^5, 0.01-3.96, -23-(-5) (*p*-channel)	Si/SiO$_2$/PS-brush/**Ph-BTBT-C$_{10}$**/Au	Vacuum	Yun *et al.* [7]
		Cyclohexanone	10^4-10^9, 0.01-5.07, -16-(-6) (*p*-channel)			
		Diethyl Carbonate	10^5-10^9, 0.04-3.22, -18-(-6) (*p*-channel)			
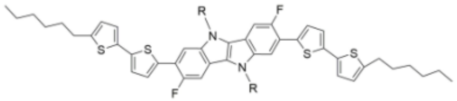<br>**PEG-IDIDF**	Solution-shearing	Ethanol-water	10^3-10^6, 5.53×10^{-4}-2.01×10^{-3}, (-1±4)-(1±3) (*p*-channel)	Si/SiO$_2$/PVN/**PEG-IDIDF**/Au	Under N_2	Hong *et al.* [8]

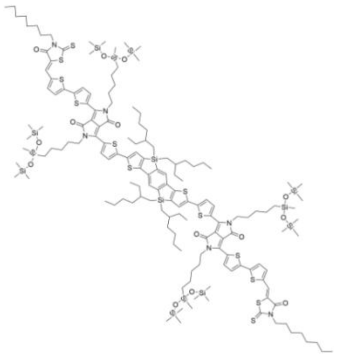<br>**LGC-D118**	Spin-coating	2-methyl tetrahydro furan	0.82×10^{6}, 2.60, -5.11±1.58 (*p*-channel)	Glass/Au-Ni/**LGC-D118**/CYTOP/Al	Under N_2	Lim *et al.* [9]
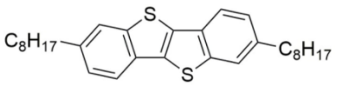<br>**C_8-BTBT**	Spin-coating	Cyclohexanone	10^{7},<br>4.6,<br>-7.0±1.9<br>(*p*-channel)	Glass/PVP/Au-Cr/**C_8BTBT**/CYTOP/Al	Vacuum	Sanda *et al.* [10]
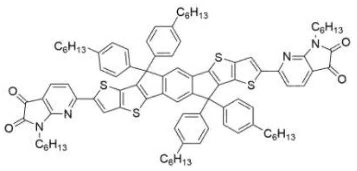<br>**IDTT-IDD-N**	Spin-coating	Ethyl acetate	3.13×10^{6} - 2.48×10^{7},<br>0.54-1.49,<br>-29.5-(-14.2)<br>(*p*-channel)	Si/SiO_2/OTS/**IDTT-IDD-N**/Au	Vacuum	Zhang *et al.* [11]
	Spin-coating	Tetrahydrofuran	9.81×10^{6}- 4.43×10^{7},<br>0.46-1.01,<br>-27.1-(-13.3)<br>(*p*-channel)	Si/SiO_2/OTS/**IDTT-IDD-N**/Au	Vacuum	

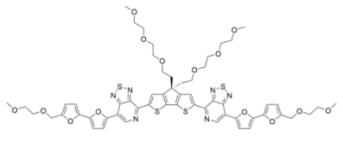 **4**	Spin coating	Ethyl acetate	10^3, 10^{-5}, NA (*p*-channel)	Si/SiO_2/HMDS/**4**/Au	Vacuum	Henson *et al.* [12]
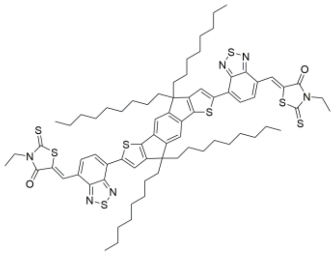 **O-IDTBR**	Blade-coating	Eucalyptol	>10^5, 0.37-0.91, 38.8 (*n*-channel)	Glass/Au/**O-IDTBR**/CYTOP CTL-809M/Al	Under N_2	Corzo *et al.* [13]
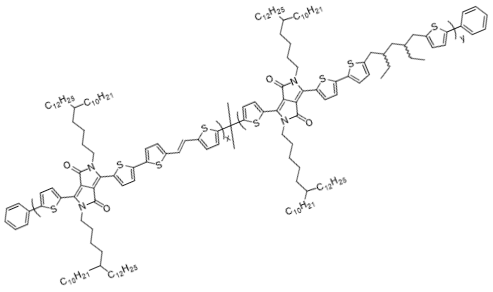 **DPP-10C$_5$DE**	Solution-shearing	Mesitylene	>10^5, 2.03, 0.7 (*p*-channel)	Si/SiO_2/OTMS/ **DPP-10C$_5$DE**/Au	Under N_2	Wang *et al.* [14]
	Solution-shearing	2-methyl tetrahydro furan	>10^6, 1.02, 12.4 (*p*-channel)			
	Solution-shearing	*p*-xylene	>10^5, 1.69, -3.7 (*p*-channel)			

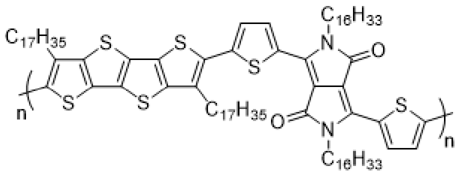 **PTDPPTFT4**	Spin coating	*p*-xylene	~10^6, 0.47-2.1, 9.7-15 (*p*-channel)	Si/SiO_2/ OTS-**C8/PTDPPTFT4**/Au	Under N_2	Matthews *et al.* [15]
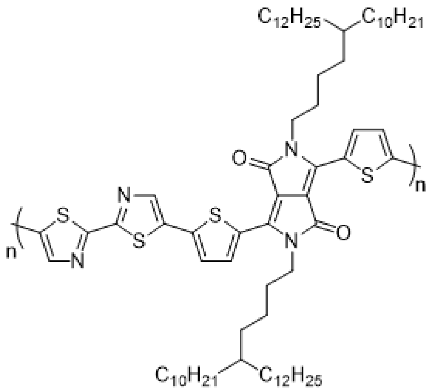 **PDBTz**	Spin coating	*p*-xylene	10^5, 0.31, 4 (*n*-channel)	Si/SiO_2/BCB/ **PDBTz/**Ca-Al	Under N_2	Fu *et al.* [16]

Table S2. The molecular structures, purification methods, solubility values (if available), highest occupied molecular orbital (HOMO) energy levels, organic field-effect transistor (OFET) semiconductor thin-film processing method, and the maximum field-effect charge carrier mobilities for previously reported BTBT-based semiconductors.

Compound	Molecular Structure	Purification Method (Solubility Value - if available)	HOMO Energy Level	OFET Semiconductor Thin-Film Processing Method (Maximum Field-Effect Charge Carrier Mobility)	Reference
**1 (DPh-BTBT)**	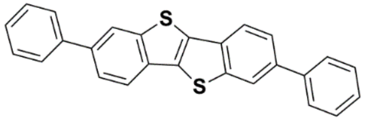	Vacuum Sublimation	-5.60 eV	Thermal Evaporation (1.0-2.0 $cm^2/V.s$)	[17]
**2 (C_6-BTBT)**	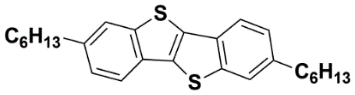	Chromatography (70 g/L in Chloroform)	-5.50 eV	Spin-Coating from chloroform (0.36-0.45 $cm^2/V.s$)	[18]
**3 (C_8-BTBT)**	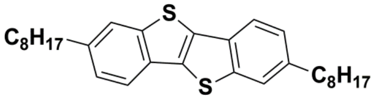	Chromatography (≈10 g/L in toluene, *p*-xylene, and cyclohexanone)	not disclosed	Spin-Coating from toluene:*p*-xylene (4.9 $cm^2/V.s$)	[10]
		Chromatography	not disclosed	Off-Centre Spin-Coating from *o*-dichlorobenzene (25-43 $cm^2/V.s$)	[19]
**4 (BTBT-Ph-C_{12})**	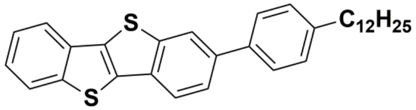	Vacuum Sublimation	-5.64 eV	Thermal Evaporation (8.7 $cm^2/V.s$)	[20]
**5**	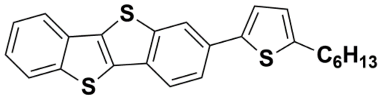	Chromatography	-5.48 eV	Thermal Evaporation (10.5 $cm^2/V.s$)	[21]

**6**	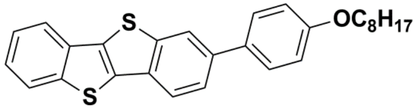	Vacuum Sublimation	-5.51 eV	Thermal Evaporation (8.25 cm^2/V.s)	[22]
**7 (BTBT-Ph-C_6)**	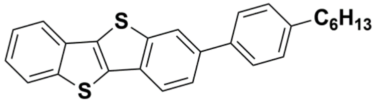	Vacuum Sublimation	-5.65 eV	Thermal Evaporation (4.6 cm^2/V.s)	[23]
**8**	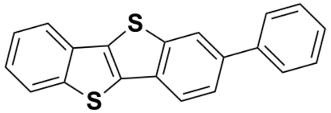	Vacuum Sublimation	-5.68 eV	Thermal Evaporation (0.034 cm^2/V.s)	[23]
**9**	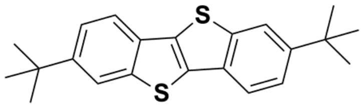	Recrystallization/ Chromatography	-5.70 eV	Physical Vapor Transport/Solution Shearing from tetralin (17/3.7 cm^2/V.s)	[24]
**10**	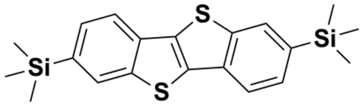	Recrystallization/ Chromatography	-5.60 eV	Physical Vapor Transport (0.6 cm^2/V.s)	[24]
**11**	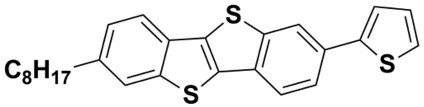	Chromatography	-5.45 eV	Solution Shearing from toluene/1,2,4-trichlorobenzene (0.1 cm^2/V.s)	[25]
**12**	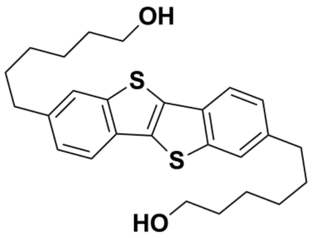	Recrystallization	-5.56 eV	Thermal Evaporation (0.17 cm^2/V.s)	[26]

**13**	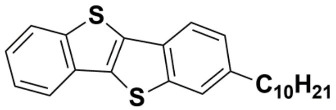	Recrystallization	-5.80 eV	Thermal Evaporation<br>(6 cm^2/V.s)	[27]
**14**<br>**((mono)-C_{13}-BTBT)**	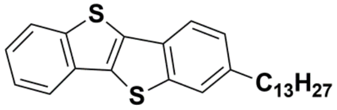	Recrystallization	-5.80 eV	Thermal Evaporation<br>(17.2 cm^2/V.s)	[28]
**15**<br>**(Ph-BTBT-C_{10})**	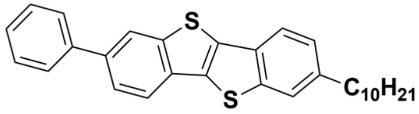	Recrystallization	not disclosed	Spin-Coating from diethylbenzene<br>(14.7 cm^2/V.s)	[29]
**16**	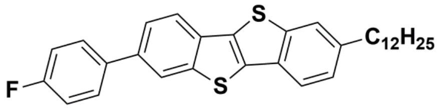	Recrystallization	not disclosed	Solution-Casting from *p*-xylene<br>(0.42 cm^2/V.s)	[30]
**17**<br>**(HexT-BTBT)**	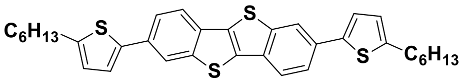	Chromatography and Crystallization (hexane)	-5.68 eV	Thermal Evaporation<br>(0.0085cm^2/V.s)	[31]
**18**<br>**(EHexT-BTBT)**	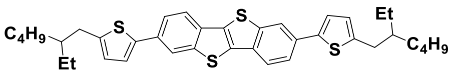	Chromatography and Crystallization (hexane)	-5.62 eV	Thermal Evaporation<br>(0.11cm^2/V.s)	[31]
**19**<br>**(O-2Th-BTBT)**	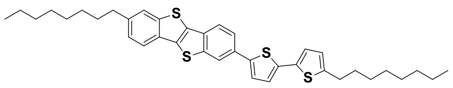	Chromatography (hot hexane) and Crystallization (heptane)	-5.30 eV	Solution Shearing<br>(0.19 cm^2/V.s)	[32]

**20 (E-2Th-BTBT)**	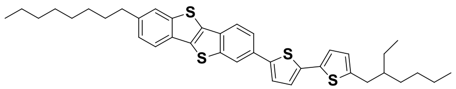	Chromatography (hot hexane) and Crystallization (heptane)	-5.30 eV	Solution Shearing (0.022 cm^2/V.s)	[32]
**21 (2Th-BTBT)**	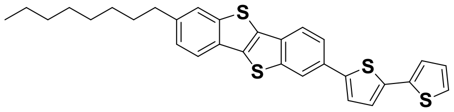	Chromatography (hot hexane) and Crystallization (heptane)	-5.36 eV	Solution Shearing (0.31 cm^2/V.s)	[32]
**22 (Compound 1)**	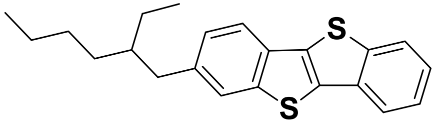	Chromatography (hexane)	-5.73 eV	Solution Shearing (0.013 cm^2/V.s)	[33]
**23 (Compound 2)**	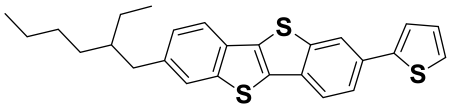	Crystallization (dichloromethane)	-5.51 eV	Solution Shearing (0.12 cm^2/V.s)	[33]
**24 (Compound 3)**	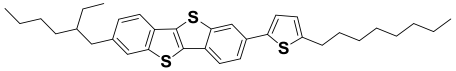	Chromatography (hexane)	-5.48 eV	Solution Shearing (0.0067 cm^2/V.s)	[33]
**25 (Compound 4)**	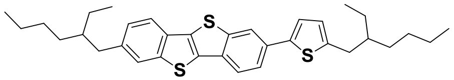	Chromatography (hexane)	-5.48 eV	Solution Shearing (0.0062 cm^2/V.s)	[33]
**26 (Ph-BTBT-10)**	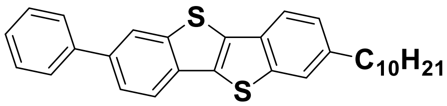	Commercial sources without purification	not disclosed	Solution Shearing from PhCI solution (0.48 cm^2/V.s)	[34]

**27 (An-BTBT)**	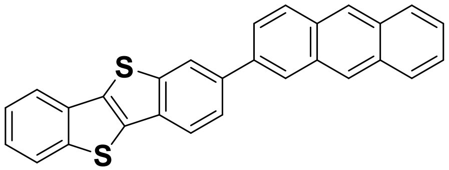	Vacuum Sublimation	-5.74 eV	Physical Vapor Transport (0.82 $cm^2/V.s$)	[35]
**28 (An-BTBT-An)**	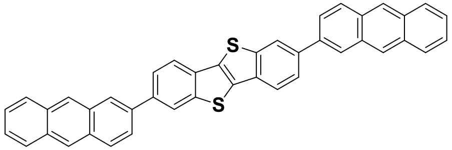	Vacuum Sublimation	-5.70 eV	Physical Vapor Transport (1.20 $cm^2/V.s$)	[35]
**29 (BTBT-An-BTBT)**	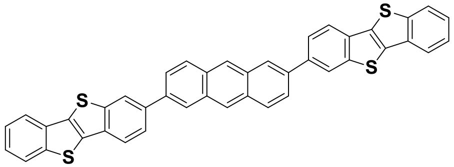	Vacuum Sublimation	-5.69 eV	Physical Vapor Transport (0.22 $cm^2/V.s$)	[35]

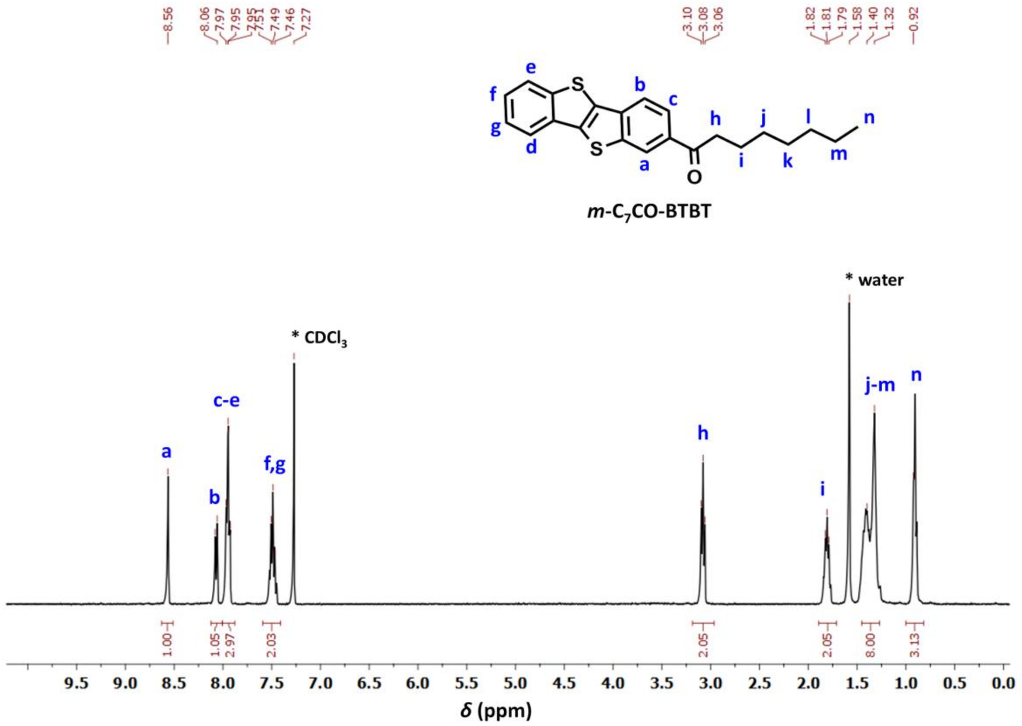

Figure S1. ^{1}H NMR spectrum of 1-(benzo[*b*]benzo[4,5]thieno[2,3-*d*]thiophen-2-yl)octan-1-one (***m*-C$_7$CO-BTBT**) in $CDCl_3$ at room temperature.

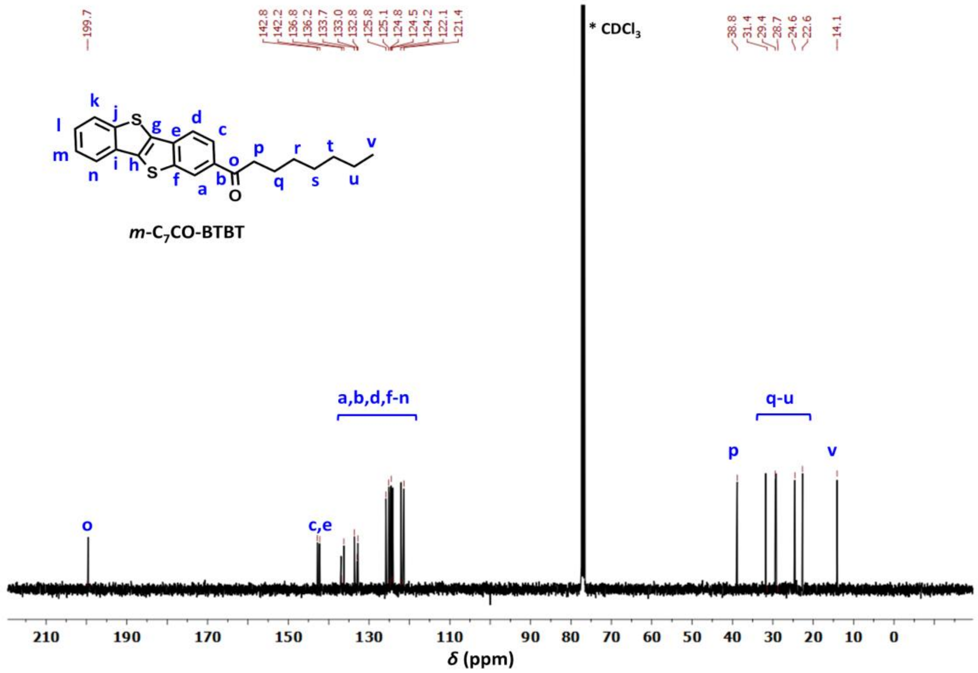

Figure S2. ^{13}C NMR spectrum of 1-(benzo[*b*]benzo[4,5]thieno[2,3-*d*]thiophen-2-yl)octan-1-one (***m*-C$_7$CO-BTBT**) in $CDCl_3$ at room temperature.

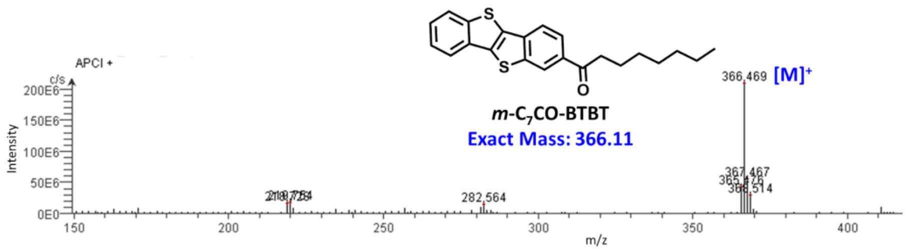

Figure S3. Positive ion mass spectrum of 1-(benzo[*b*]benzo[4,5]thieno[2,3-*d*]thiophen-2-yl)octan-1-one (***m*-C$_7$CO-BTBT**) measured by atmospheric pressure chemical ionization mass spectrometer (APCI-MS).

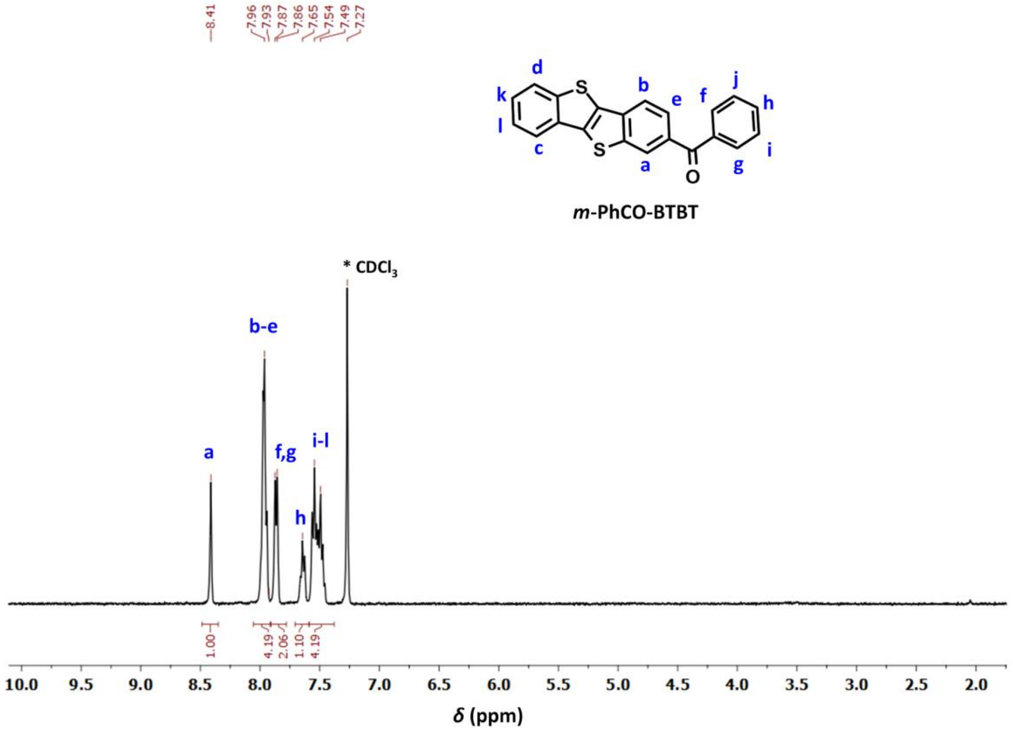

Figure S4. ^{1}H NMR spectrum of benzo[*b*]benzo[4,5]thieno[2,3-*d*]thiophen-2-yl(phenyl)methanone (***m*-PhCO-BTBT**) in CDCl$_3$ at room temperature.

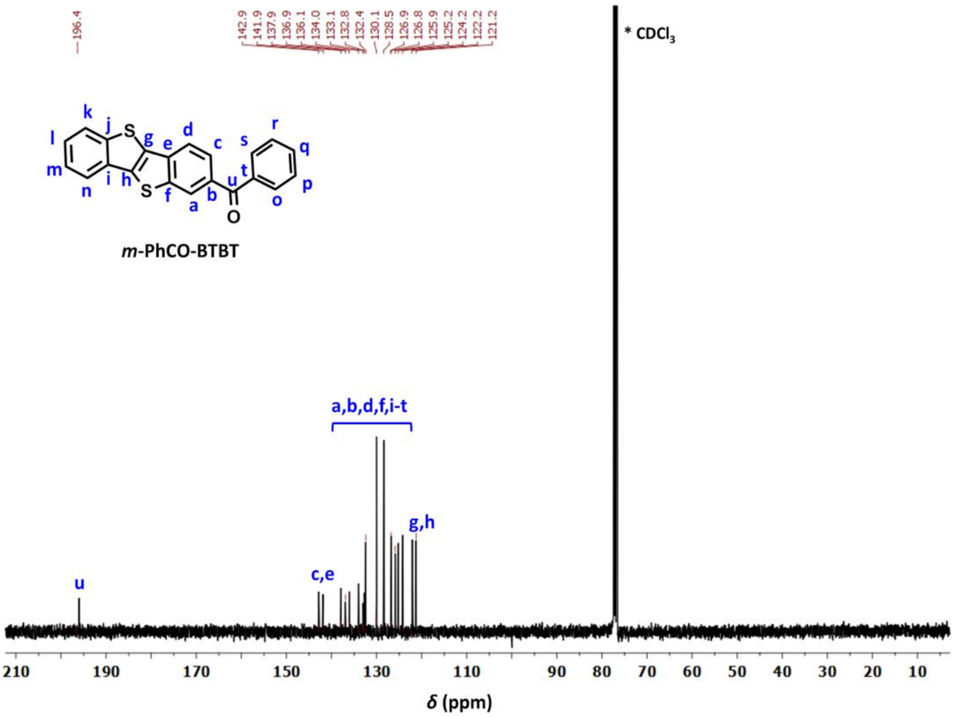

Figure S5. ^{13}C NMR spectrum of benzo[*b*]benzo[4,5]thieno[2,3-*d*]thiophen-2-yl(phenyl)methanone (***m*-PhCO-BTBT**) in $CDCl_3$ at room temperature.

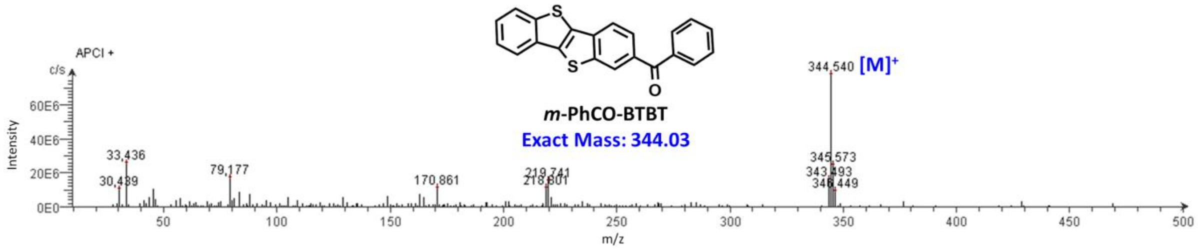

Figure S6. Positive ion mass spectrum of benzo[*b*]benzo[4,5]thieno[2,3-*d*]thiophen-2-yl(phenyl)methanone (***m*-PhCO-BTBT**) measured by atmospheric pressure chemical ionization mass spectrometer (APCI-MS).

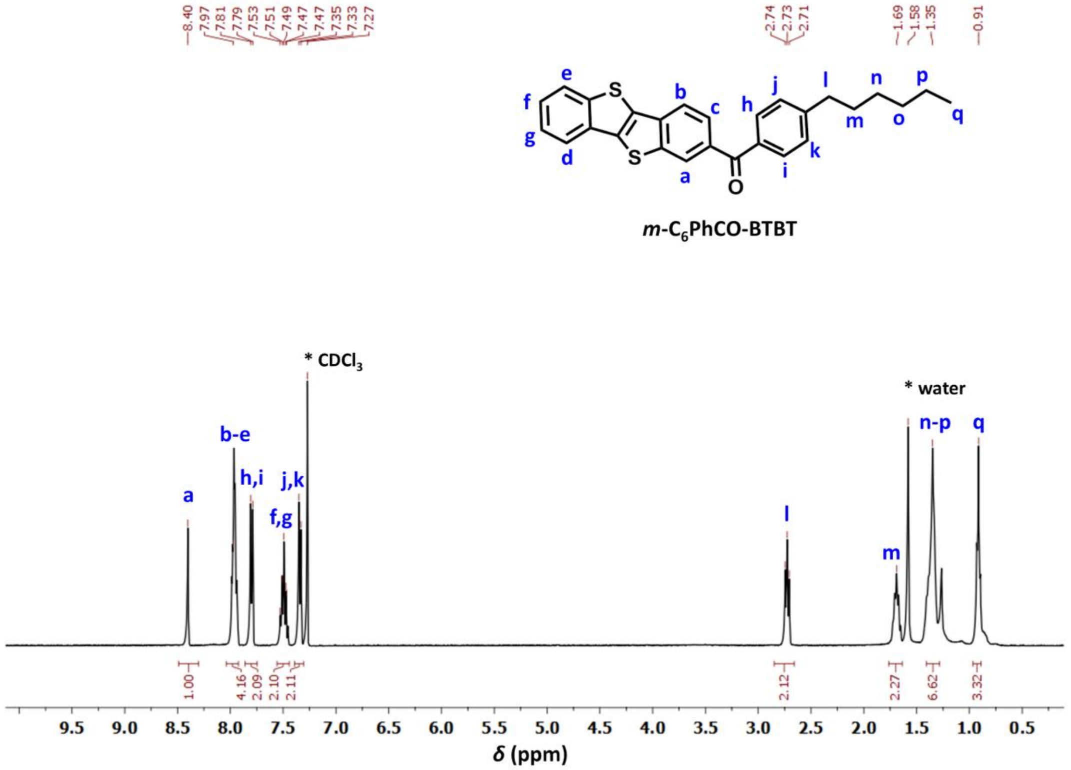

Figure S7. ^{1}H NMR spectrum of benzo[*b*]benzo[4,5]thieno[2,3-*d*]thiophen-2-yl(4-hexylphenyl)methanone (***m*-C$_6$PhCO-BTBT**) in $CDCl_3$ at room temperature.

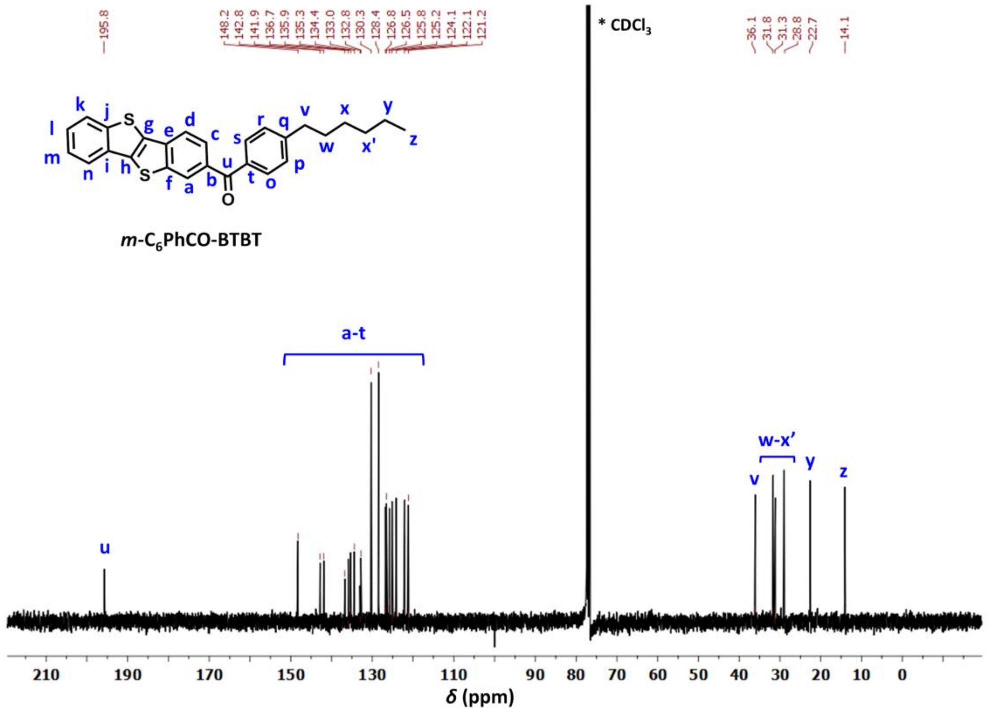

Figure S8. ^{13}C NMR spectrum of benzo[*b*]benzo[4,5]thieno[2,3-*d*]thiophen-2-yl(4-hexylphenyl)methanone (***m*-C$_6$PhCO-BTBT**) in $CDCl_3$ at room temperature.

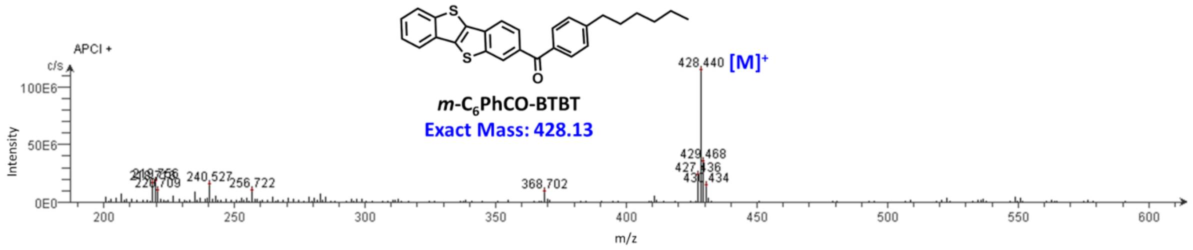

Figure S9. Positive ion mass spectrum of benzo[*b*]benzo[4,5]thieno[2,3-*d*]thiophen-2-yl(4-hexylphenyl)methanone (***m*-C$_6$PhCO-BTBT**) measured by atmospheric pressure chemical ionization mass spectrometer (APCI-MS).

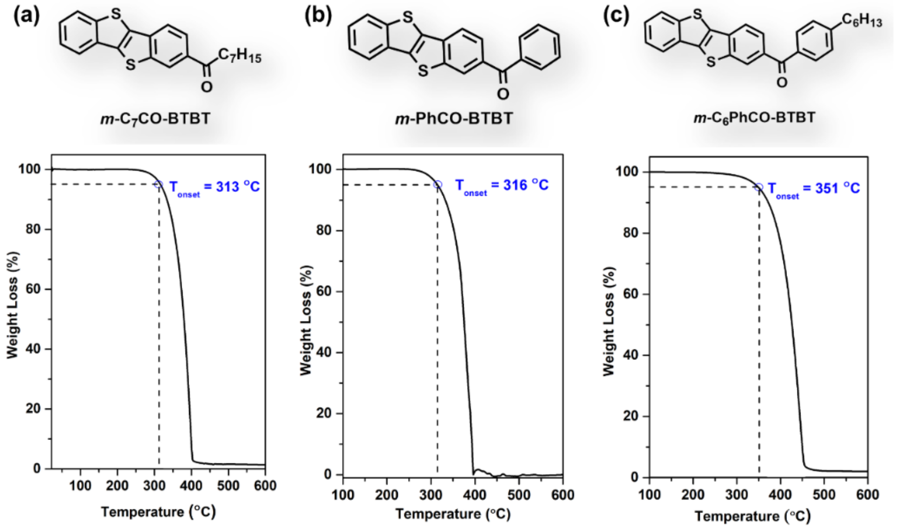

Figure S10. Thermogravimetric analysis (TGA) of mono-carbonyl BTBT small molecules***m*-C$_7$CO-BTBT** (a), ***m*-PhCO-BTBT** (b), and ***m*-C$_6$PhCO-BTBT** (c) at a temperature ramp of 10 °C/min under N_2.

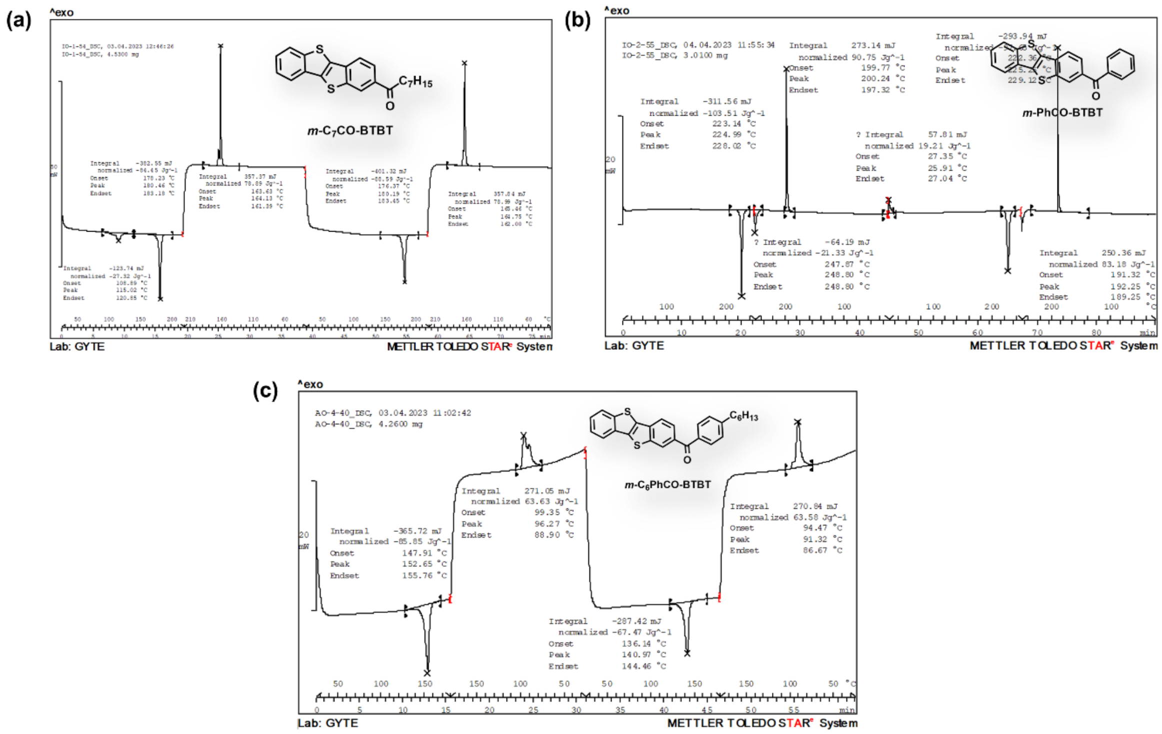

Figure S11. Differential scanning calorimetry scans (shown as raw scans from the instrument) of mono-carbonyl BTBT small molecules ***m*-C$_7$CO-BTBT** (a), ***m*-PhCO-BTBT** (b), and ***m*-C$_6$PhCO-BTBT** (c) at a temperature ramp of 10 °C min^{-1} under N_2.

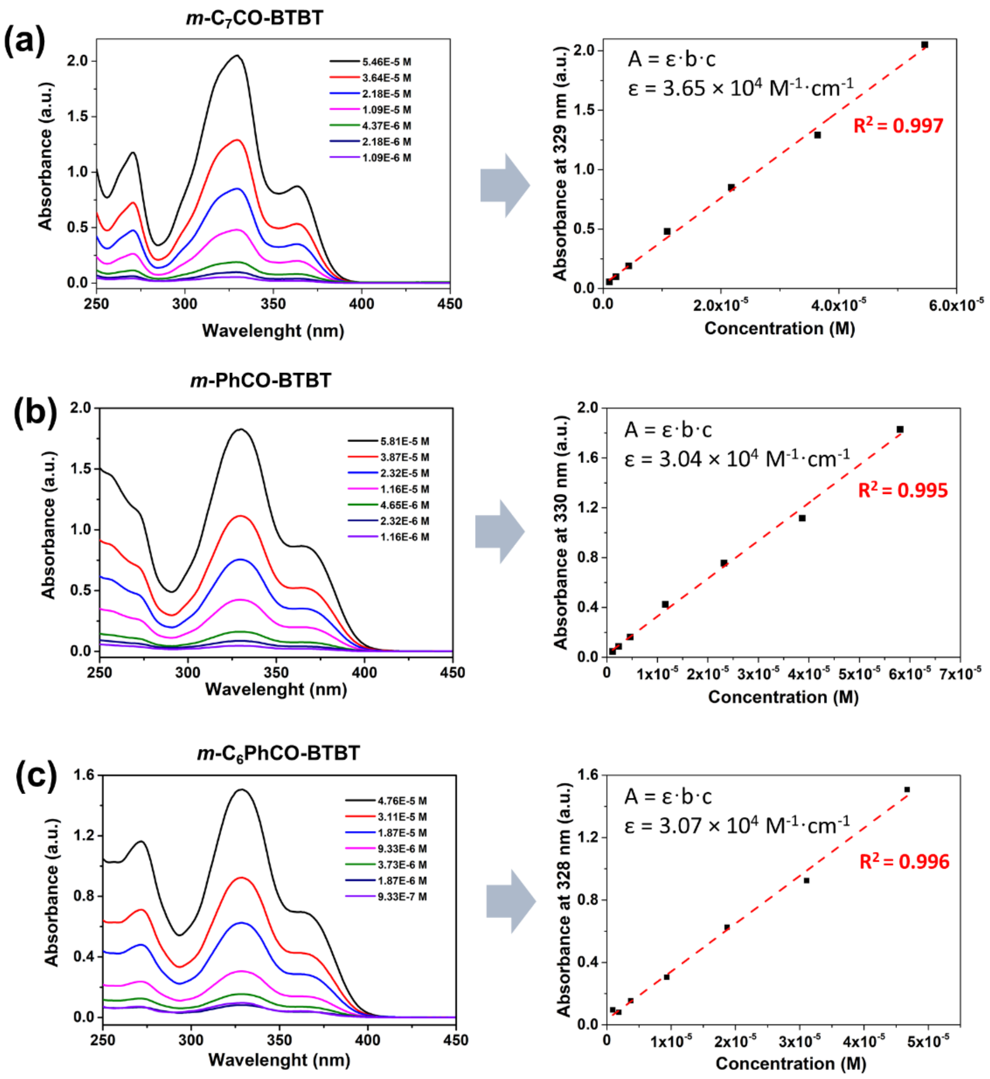

Figure S12. The calibration curve based on the absorbance of mono-carbonyl BTBT small molecules ***m*-C$_7$CO-BTBT** (a), ***m*-PhCO-BTBT** (b), and ***m*-C$_6$PhCO-BTBT** (c) in chloroform at varied standard solution concentrations recorded at their corresponding absorption maximum; the linear fitting was performed according to Beer-Lambert law (adjusted R^2's > 0.99).

Theoretical calculation of the intercept in the Hansen-adapted Scatchard-Hildebrand regular solution theory equation (1):

$$-\ln x_{OSC} = \frac{v_{OSC}}{RT}\Phi_{solv.}{}^2 R_a{}^2 + \frac{\Delta H_{fus}}{R}\left(\frac{1}{T} - \frac{1}{T_{mp-OSC}}\right) \quad (1)$$

By using the thermal properties of enthalpy of fusion (ΔH_{fus}) and melting temperature (T_{mp-OSC}) measured in the DSC scans of the current molecules (Figure S11), the intercept in equation (1) is calculated as shown in Table S2. Note that all these melting processes lead to a fully isotropic liquid phase, as visually confirmed through conventional melting point measurement. The endset of the melting temperature and enthalpy of fusion in the first-heating cycleare taken as it represents the completion of the melting process for the polycrystalline solid obtained in the synthesis. Note that the same polycrystalline solid is used for solubility measurements to determine the HSP sphere. When another endothermic transition is observed prior to the main melting process (as seen in the case of ***m*-C$_7$CO-BTBT**, Figure S11(a)), this corresponding prior enthalpy value and the thermal transition end set temperature (27.32 J/g at 120.85 °C) is taken into account for calculating the intercept value, as this endothermic transition is a part of the solid-to-isotropic liquid transition.

Table S3. The thermal properties of melting temperature end set ($T_{mp-endset}$) and enthalpy of fusion (ΔH_{fus}) measured in the DSC scans, molecular weights, and the calculated intercept values based on equation (1) shown above for the mono-carbonyl BTBT small molecules ***m*-C$_7$CO-BTBT**, ***m*-PhCO-BTBT**, and ***m*-C$_6$PhCO-BTBT**.

Molecule	$T_{mp-endset}$ (°C)	ΔH_{fus} (J/g)	Molecular Weight (g/mol)	ΔH_{fus} (J/mol)	Intercept
m*-C$_7$CO-BTBT	183.18	84.45	366.54	40968.1758	5.30
***m*-PhCO-BTBT**	228.02	103.51	344.45	35654.0195	5.83
***m*-C$_6$PhCO-BTBT**	155.76	85.85	428.61	36796.1685	4.52

* The prior endothermic transition is also taken into account for calculating the intercept value, as explained above.

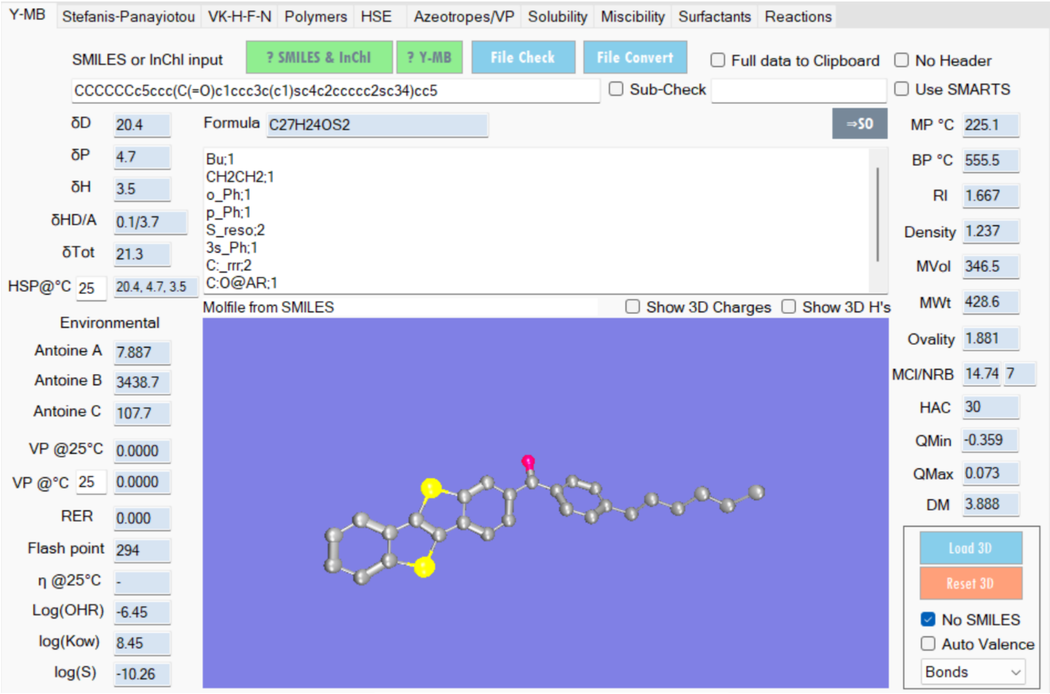

Figure S13. Hansen solubility parameters calculated for ***m*-C$_6$PhCO-BTBT** by group contribution methodology in the HSPiP software (*5th Edition Version 5.4.08*) with DIY and YMB modules using simplified molecular-input line-entry system (SMILES).

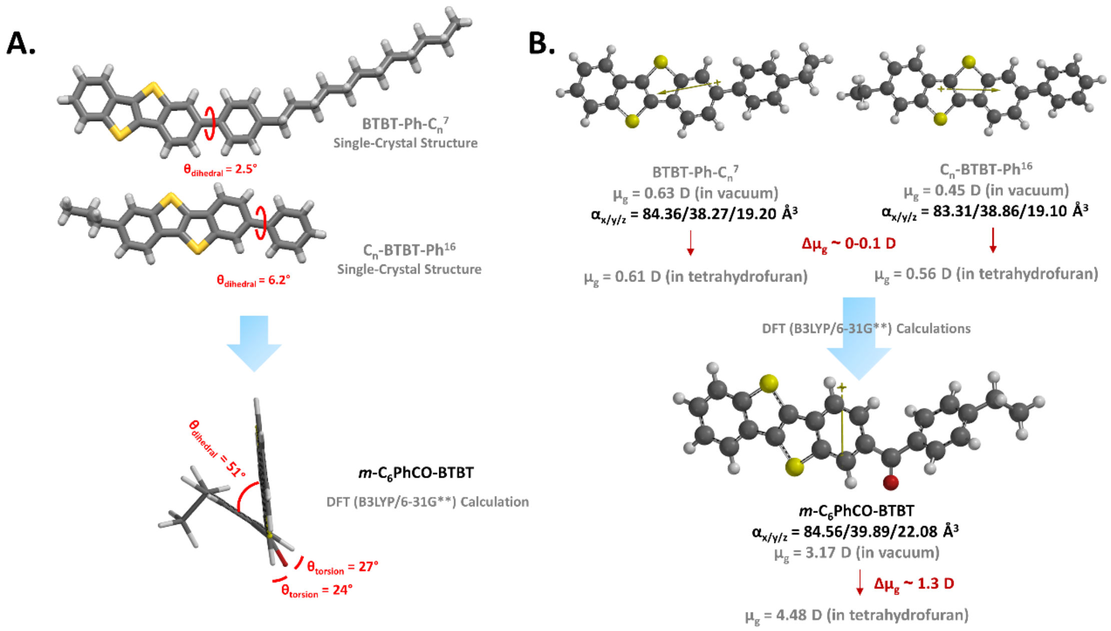

Figure S14. A. (*BTBT*)Ph-Ph dihedral angles for structurally similar **BTBT-Ph-C$_n$**and **C$_n$-BTBT-Ph**molecules based on their reported single-crystal structures,[22,36] and the DFT(B3LYP/6-31G**)-optimized molecular conformation of ***m*-C$_6$PhCO-BTBT** showing the (*BTBT*)Ph-Ph dihedral angle and (*BTBT*)Ph-CO/CO-Ph torsion angles. B. DFT(B3LYP/6-31G**)-calculated molecular dipole moments (in vacuum and in tetrahydrofuran) and polarizabilities for **BTBT-Ph-C$_n$**, **C$_n$-BTBT-Ph**,and***m*-C$_6$PhCO-BTBT**indicating a significant increase in ground-state dipole moments (μ_g (D)), dipole changes ($\Delta\mu_g$ (D)) going from vacuum to solution, and polarizabilities (α ($Å^3$)) upon carbonyl insertion.

The Solvatochromic Lippert-Mataga Model:

The Lippert-Mataga model is used according to the following equation (2) to estimate the dipole moments of S_1 state:

$$\nu_a - \nu_f = \frac{2(\mu_e - \mu_g)^2}{hca^3} f(\varepsilon, n) + (\nu_a^0 - \nu_f^0) \quad (2)$$

where h is Planck constant, c is the speed of light in vacuum, $\nu_a^0 - \nu_f^0$ is the Stokes shift when f is zero, μ_e and μ_g are dipole moments of excited state and ground state respectively. $f(\varepsilon, n)$ and a are the solvent orientation polarizability and the solvent Onsager cavity radius, respectively and can be calculated according to the following equation (3):

$$f(\varepsilon, n) = \frac{\varepsilon - 1}{2\varepsilon + 1} - \frac{n^2 - 1}{2n^2 + 1}, \quad a = (\frac{3M}{4N\pi d})^{1/3} \quad (3)$$

where ε is the solvent dielectric constant and n is the solvent refractive index. M is the molar mass, N is the Avogadro's constant, and d is the density of the solvents (d = 1.0 g/cm^3).

In the Equation (2), $\frac{2(\mu_e - \mu_g)^2}{hca^3}$ is the term corresponding to the slope of the plot of Stokes shift $(\nu_a - \nu_f)$ versus the solvent orientation polarizability $f(\varepsilon, n)$. By estimating the dipole moment of ***m*-C$_6$PhCO-BTBT**'s ground state ($\mu_g = 3.17$ D) from density functional theory (DFT) calculations and the slope from the plot of $(\nu_a - \nu_f)$ vs $f(\varepsilon, n)$, we could calculate the dipole moment of ***m*-C$_6$PhCO-BTBT**in the excited state, μ_e.

Table S4. Solvatochromic optical absorption and photoluminescence peak maxima (λ_{abs}^{max} and λ_{fl}^{max}) and Stokes shifts ($\nu_{abs} - \nu_{fl}$) for ***m*-C$_6$PhCO-BTBT** in different solvents with increasing polarity (hexanes → acetonitrile), and the solvent orientational polarizability values ($f(\varepsilon,n)$).

**Solvents**	***f (ε,n)***	λ_{abs}^{max}**(nm)**	λ_{fl}^{max}**(nm)**	$\nu_{abs} - \nu_{fl}$**(cm^{-1})**
Hexane	0.001	360	418	3854
Toluene	0.014	362	414	3470
Isopentyl Ether	0.0747	362	426	4160
Chloroform	0.148	364	437	4589
THF	0.210	361	430	4445
DCM	0.217	362	435	4636
DMF	0.276	362	444	5101
Acetonitrile	0.305	360	450	5555

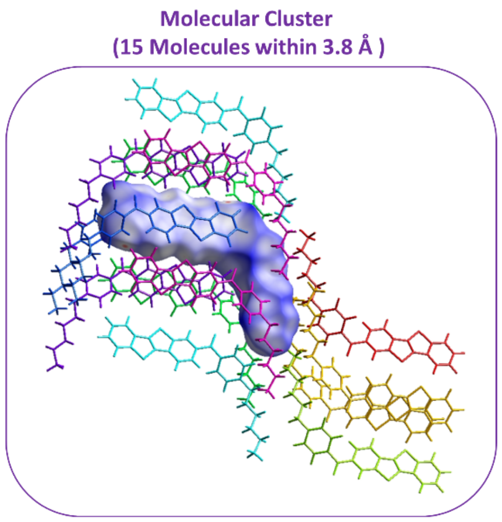

$$\text{NNSE} = \sum_{\text{Interaction Energies}} \frac{N \times Etot}{2} \quad (4) \quad = -166.0 \text{ kJ/mol}$$

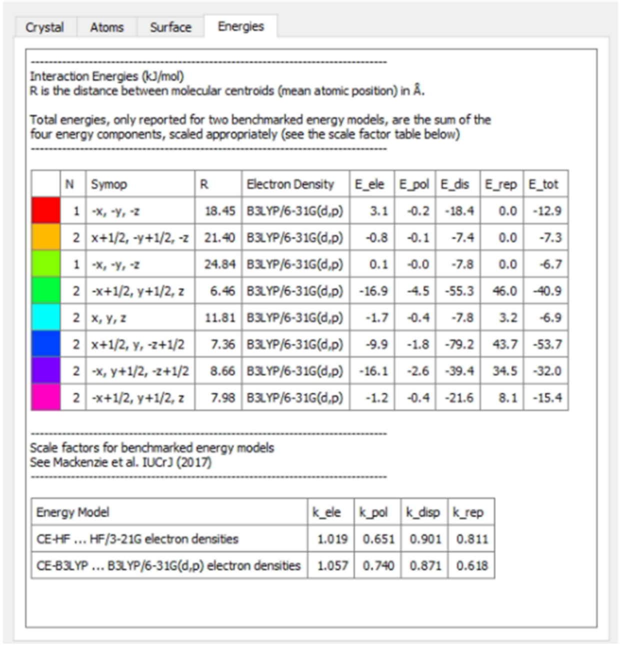
Crystal | Atoms | Surface | Energies

Interaction Energies (kJ/mol)
R is the distance between molecular centroids (mean atomic position) in Å.

Total energies, only reported for two benchmarked energy models, are the sum of the four energy components, scaled appropriately (see the scale factor table below)

	N	Symop	R	Electron Density	E_ele	E_pol	E_dis	E_rep	E_tot
(red)	1	-x, -y, -z	18.45	B3LYP/6-31G(d,p)	3.1	-0.2	-18.4	0.0	-12.9
(orange)	2	x+1/2, -y+1/2, -z	21.40	B3LYP/6-31G(d,p)	-0.8	-0.1	-7.4	0.0	-7.3
(yellow-green)	1	-x, -y, -z	24.84	B3LYP/6-31G(d,p)	0.1	-0.0	-7.8	0.0	-6.7
(green)	2	-x+1/2, y+1/2, z	6.46	B3LYP/6-31G(d,p)	-16.9	-4.5	-55.3	46.0	-40.9
(cyan)	2	x, y, z	11.81	B3LYP/6-31G(d,p)	-1.7	-0.4	-7.8	3.2	-6.9
(blue)	2	x+1/2, y, -z+1/2	7.36	B3LYP/6-31G(d,p)	-9.9	-1.8	-79.2	43.7	-53.7
(purple)	2	-x, y+1/2, -z+1/2	8.66	B3LYP/6-31G(d,p)	-16.1	-2.6	-39.4	34.5	-32.0
(magenta)	2	-x+1/2, y+1/2, z	7.98	B3LYP/6-31G(d,p)	-1.2	-0.4	-21.6	8.1	-15.4

Scale factors for benchmarked energy models
See Mackenzie et al. IUCrJ (2017)

Energy Model	k_ele	k_pol	k_disp	k_rep
CE-HF ... HF/3-21G electron densities	1.019	0.651	0.901	0.811
CE-B3LYP ... B3LYP/6-31G(d,p) electron densities	1.057	0.740	0.871	0.618

Figure S15. Pairwise intermolecular interaction energies (E_{tot}'s) of 15 ***m*-C$_6$PhCO-BTBT** molecules within a 3.8 Å distance range surrounding a central molecule (shown with a Hirshfeld Surface), computed using the DFT method with the 6-31G(d,p) basis set and CE-B3LYP model energies in CrystalExplorer21. Colors denote distinct crystallographic directions relative to the central molecule, with interaction energies computed accordingly. Atomic coordinates were obtained from crystallographic data. The formula (4) shows the calculation of nearest-neighbor shell interaction energy (NNSE) for the molecular cluster of 15 ***m*-C$_6$PhCO-BTBT** molecules.

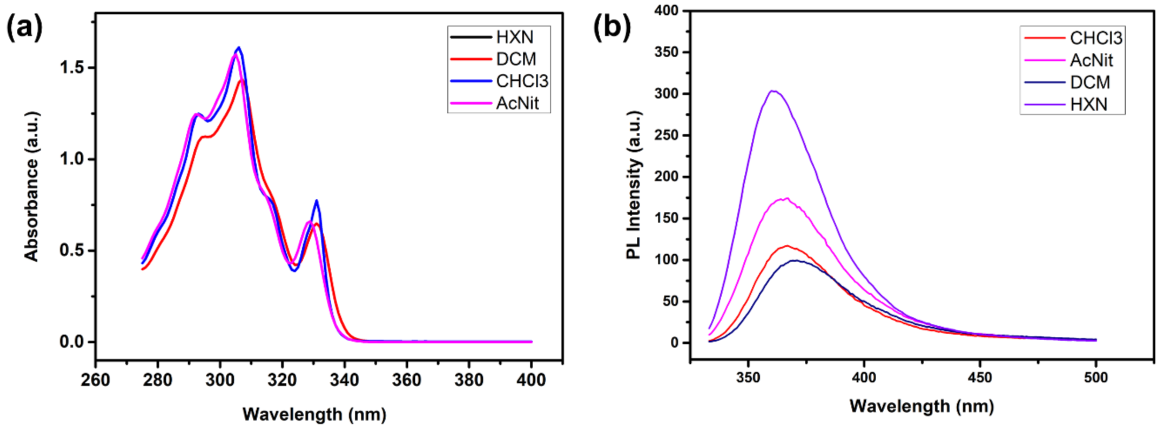

Figure S16. UV-Vis optical absorption (a) and photoluminescence ($\lambda_{excitation}$ = 330 nm) (b) spectra of **BTBT**solutions in different solvents with increasing polarity.

Table S5. Computed energy values, oscillator strengths, and configurations in canonical molecular orbital basis (with weight percentages) for the first sixsinglet excited state transitions of ***m*-C6PhCO-BTBT** calculated with TD-DFT (B3LYP/6-31G**).

**Excited State**	**Energy (eV)**	**Oscillator strength (*f*)**	**Configuration of Excited States in Canonical MO Basis**	**Weight Percentages**
S_1	3.38	0.2424	HOMO -> LUMO	84%
S_2	3.53	0.0528	HOMO-2 -> LUMO	49%
			HOMO-4 -> LUMO	25%
S_3	3.83	0.5682	HOMO-1 -> LUMO	88%
S_4	4.22	0.0020	HOMO-3 -> LUMO	66%
			HOMO -> LUMO+1	16%
S_5	4.31	0.0336	HOMO -> LUMO+1	74%
S_6	4.37	0.1079	HOMO-4 -> LUMO	39%
			HOMO-2 -> LUMO	31%
			HOMO-3 -> LUMO	10%

Intramolecular Reorganization Energy Calculations:

The intramolecular reorganization energy for hole (λ_h) and electron (λ_e) transports consists of two terms (λ_{h1}, λ_{h2} or λ_{e1}, λ_{e2}) based on the following equations (5) and (6), which corresponds to the geometrical relaxation energies in the neutral and the charged states during hole and electron transfer, respectively.

$$\lambda_h = \lambda_{h1} + \lambda_{h2} \quad (5)$$

$$\lambda_{h1} = E_{cation}\text{ (at neutral geometry) - }E_{cation}\text{ (equilibrium geometry)}$$

$$\lambda_{h2} = E_{neutral}\text{ (at cation geometry) - }E_{neutral}\text{(equilibrium geometry)}$$

$$\lambda_e = \lambda_{e1} + \lambda_{e2} \quad (6)$$

$$\lambda_{e1} = E_{anion}\text{ (at neutral geometry) – }E_{anion}\text{ (equilibrium geometry)}$$

$$\lambda_{e2} = E_{neutral}\text{ (at anion geometry) - }E_{neutral}\text{(equilibrium geometry)}$$

$E_{neutral}$ (equilibrium geometry), E_{cation} (equilibrium geometry), and E_{anion} (equilibrium geometry) are the energies of the molecule at its optimized equilibrium molecular geometries in the neutral, cation, and anion states, respectively. E_{cation} (at neutral geometry) and E_{anion} (at neutral geometry) are the single point energies of the molecular cation and anion, respectively, at the neutral molecular geometry. $E_{neutral}$ (at cation geometry) and $E_{netural}$ (at anion geometry) are the single point energies of the netural molecule at the corresponding cation and anion equilibrium geometries, respectively.

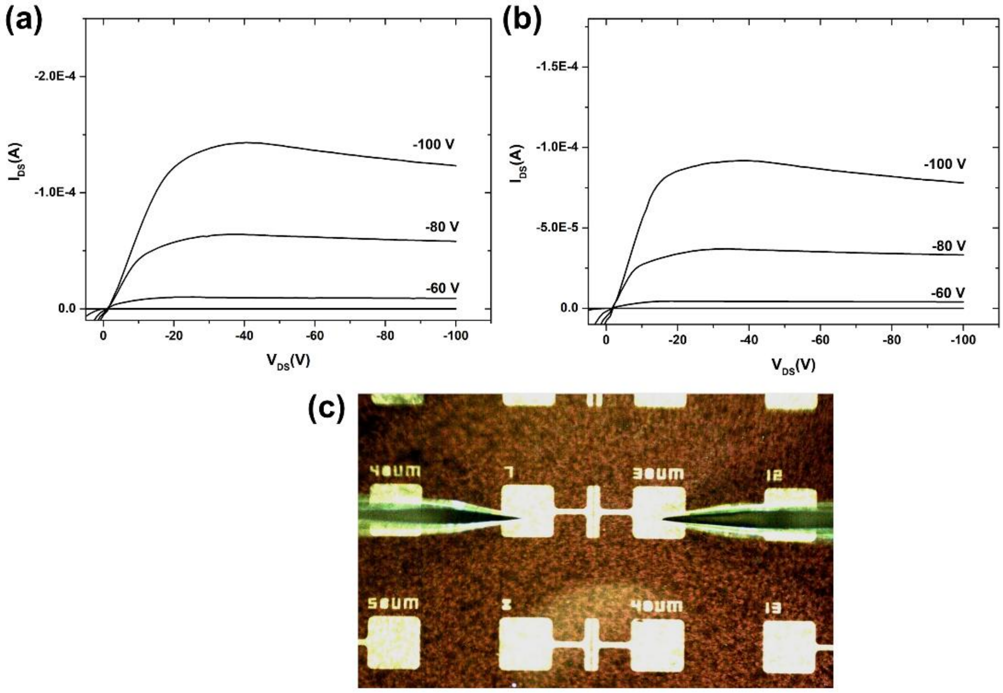

Figure S17. Output characteristics(V_{GS} = -100 V, -80 V, -60 V, -40 V, -20 V) for p^{++}-Si/SiO_2 (300 nm)/PS-brush (M_n = 5 kDa)/***m*-C$_6$PhCO-BTBT** (40-50 nm)/Au (50 nm) OFET devices with the semiconductor thin-films spin-coated from 2-methyltetrahydrofuran (a) and ethyl acetate (b), and annealed at 120 °C.(c) The top-view optical image of a section of the OFET devices during electrical characterization under ambient conditions. High density deposition mask (Ossila, E322) is used to define Au source-drain electrodes with variable channel lengths of 30, 40, 50, 60, 80 μm (Width = 1000 μm).

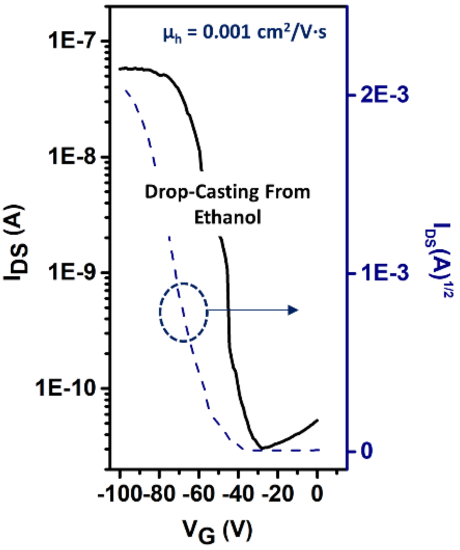

Figure S18. Transfer (V_{SD} = -100 V) characteristics for p^{++}-Si/SiO_2 (300 nm)/PS-brush (M_n = 5 kDa)/**m-C$_6$PhCO-BTBT**/Au (50 nm) OFET devices based on drop-casted semiconductor thin-films from ethanol solution (annealed at 120 °C). The $I^{1/2}$ *vs.* V_G plots used for the hole mobility calculations are shown in blue.

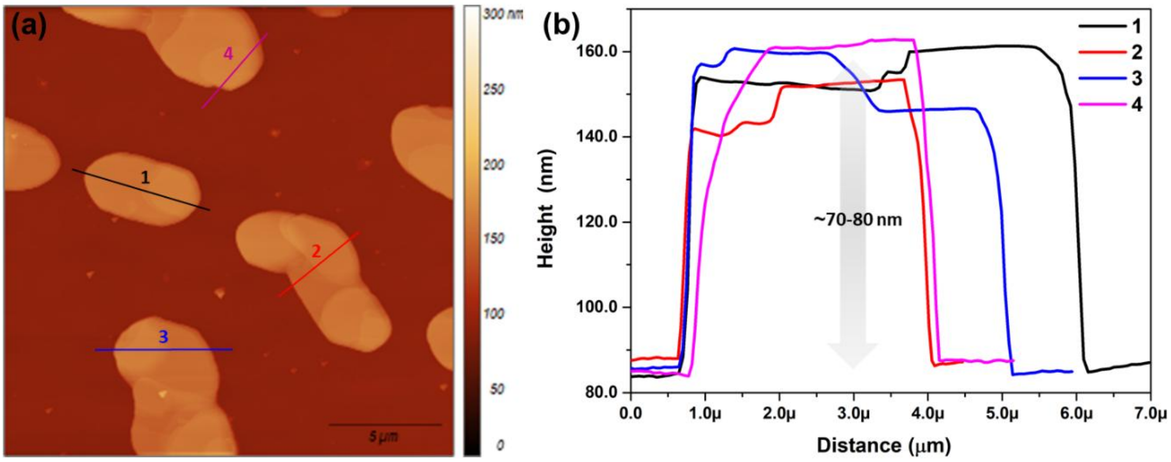

Figure S19. Tapping mode atomic force microscopy (AFM) topographic image (a) and the correspondingstep-height profiles (b) of the disintegrated micron-sized molecular domains (1-4) for ***m*-C$_6$PhCO-BTBT** thin-film on p^{++}-Si/SiO$_2$ (300 nm)/PS-brush (M$_n$ = 5 kDa) after thermal annealing at 130-140 °C.

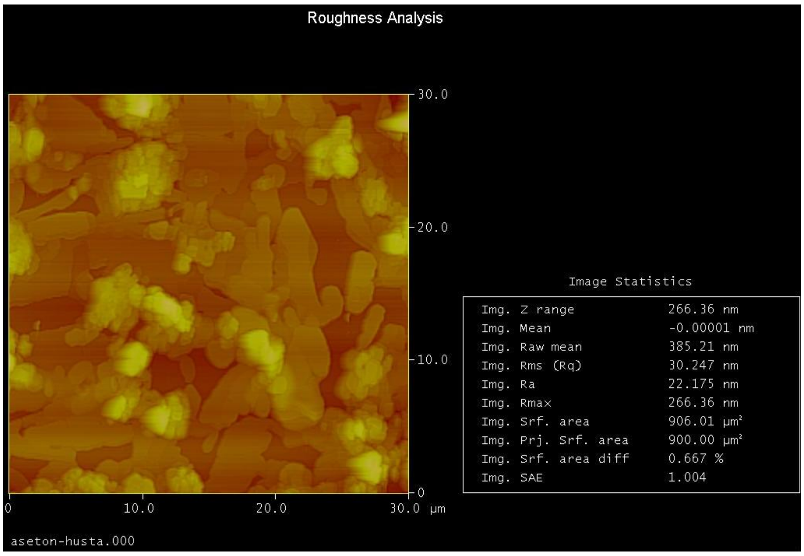

Figure S20. Tapping mode atomic force microscopy (AFM) topographic image and the roughness analysis of the spin-coated ***m*-C$_6$PhCO-BTBT** thin-film (from acetone and annealed at 120 °C) on p^{++}-Si/SiO$_2$ (300 nm)/PS-brush (M$_n$ = 5 kDa).

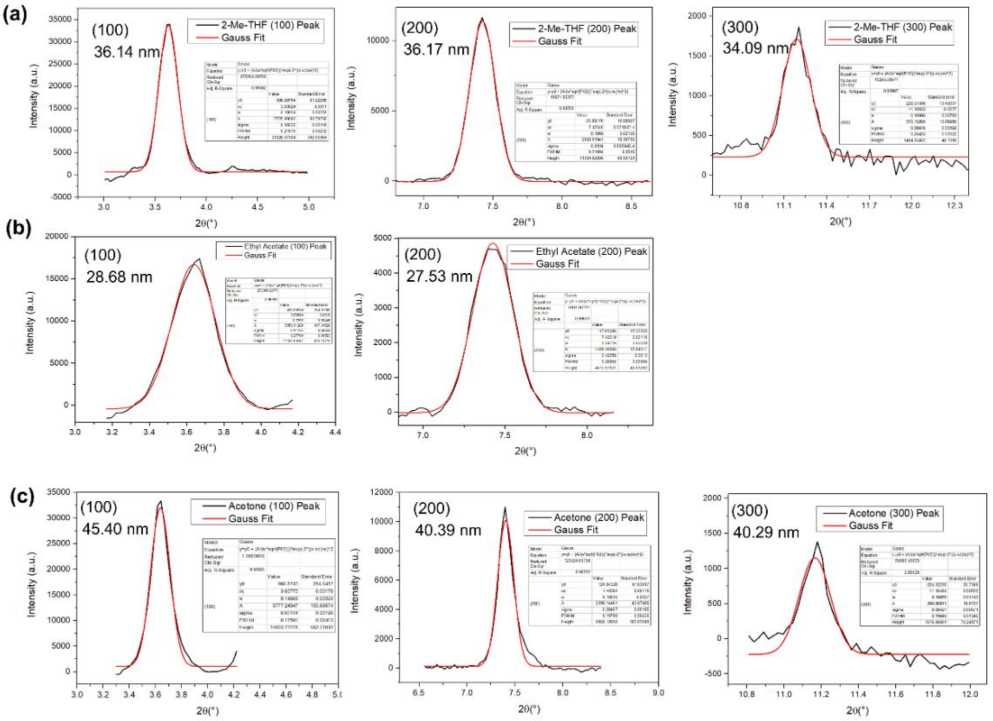

Figure S21. The Gauss fittings for the ***m*-C$_6$PhCO-BTBT**(100), (200), and (300) XRD peaks recorded in thin-films from 2-methyltetrahydrofuran (a), ethyl acetate (b), and acetone (c),and the corresponding Adj. R-squared, β (fwhm), and 2θ (peak position) values to be used in the Scherrer Equation (L = K·λ/β·cosθ) in which K is the Scherrer constant (0.9), λ is the wavelength of the radiation for the Cu K_α X-ray source (0.15406 nm), and β (in radians) and θ (in degrees) are the fwhm and the peak position values for the diffraction peaks, respectively.

Reliability Factor and Effective Mobility

It is very important to obtain an extended linear region in the $I_{DS}^{1/2}$*vs.* V_{GS} plots to define the ideal FET device operation and to use the Shockley model as the physical model for FET operation. The saturation mobilities (μ_{sat}) for our present OFETs (i.e., processed from 2-methyltetrahydrofuran, ethyl acetate, acetone, and ethoxybenzene) are calculated from these extended linear regions in the $I_{DS}^{1/2}$*vs.* V_{GS} transfer plots over a wide V_{GS} voltage ranges of >50 V. However, a high V_{th} is indicative of high deep trap density and carrier injection problems. Therefore, an electrically equivalent ideal FET is defined to exhibit the same I_{DS}^{max} at the V_{GS}^{max} and to operate as an ideal transistor according to the Shockley FET equations (i.e., a linear transfer characteristics with an ideal V_{th} = 0 V). According to these definitions, a reliability factor (r_{sat}) and effective mobility (μ_{eff}) are defined using the following equation (7):

$$r_{sat} = \frac{\left(\frac{\sqrt{|I_{ds}|^{max}} - \sqrt{|I_{ds}|^{0}}}{|V_{gs}|^{max}} \right)^2}{\left(\frac{\sqrt{|I_{ds}|}}{V_{gs}} \right)^2_{claimed}} \quad (7)$$

Note that the squared slope for the dashed-black and dashed-red linear fits in **Figure S22** corresponds to the numerator and denominator, respectively, in the above equation. The effective mobility (μ_{eff}) could be calculated using the equation $\mu_{eff} = r_{sat} \times \mu_{sat}$. Due to our relatively large V_{th}, the reliability factors reach the highest values of 32-38%.

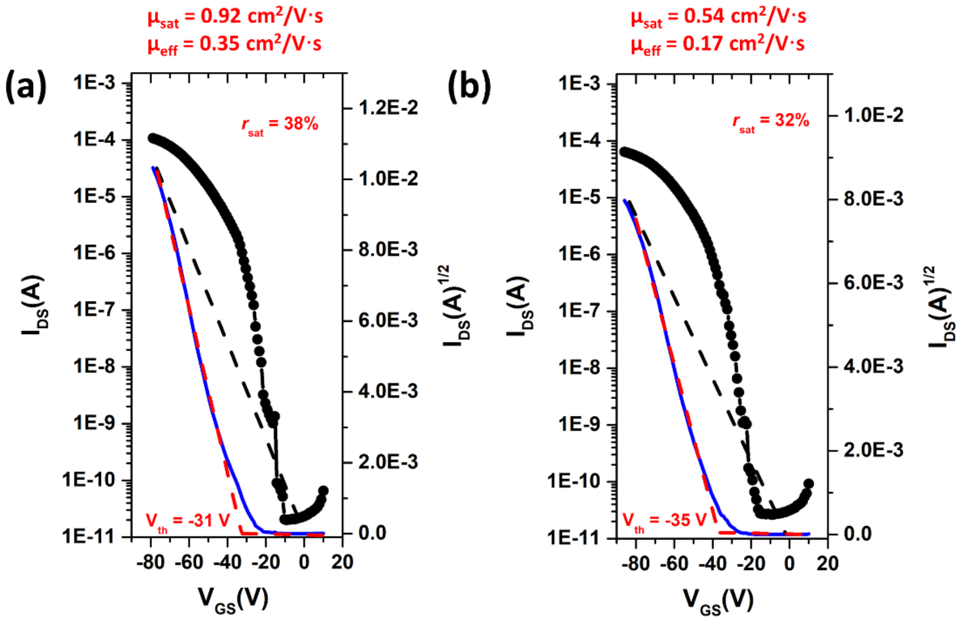

Figure S22. The reliability factors(r_{sat}'s) and the effective mobilities (μ_{eff}'s) calculated based on the transfer curves of the p^{++}-Si/SiO_2/PS-brush (M_n = 5 kDa)/***m*-C₆PhCO-BTBT**/AuOFET devices in which the semiconductor layer is spin-coated from 2-methyltetrahydrofuran (a) and ethyl acetate (b).

REFERENCES

[1] D. Ho, J. Lee, S. Park, Y. Park, K. Cho, F. Campana, D. Lanari, A. Facchetti, S. Seo, et al., "Green solvents for organic thin-film transistor processing," *J. Mater. Chem. C* **8**, 5786–5794 (Royal Society of Chemistry, 2020).

[2] D. H. Harris, S. Brixi, B. S. Gelfand, and G. C. Welch, "A N–H functionalized perylene diimide with strong red-light absorption for green solvent processed organic electronics," *J. Mater. Chem. C* **8**, 9811–9815 (2020).

[3] Y. Lee, D. Ho, F. Valentini, T. Earmme, A. Marrocchi, L. Vaccaro, and C. Kim, "Improving the charge transport performance of solution-processed organic field-effect transistors using green solvent additives," *J. Mater. Chem. C* **9**, 16506–16515 (Royal Society of Chemistry, 2021).

[4] M. Lee, S. Yun, D. Ho, T. Earmme, A. Marrocchi, L. Vaccaro, and C. Kim, "Green solvent-processed complementary-like inverters based on ambipolar organic thin-film transistors," *J. Ind. Eng. Chem.* **105**, 231–237 (The Korean Society of Industrial and Engineering Chemistry, 2022).

[5] H. Opoku, B. Nketia-Yawson, E. S. Shin, and Y. Y. Noh, "Controlling organization of conjugated polymer films from binary solvent mixtures for high performance organic field-effect transistors," *Org. Electron.* **41**, 198–204 (Elsevier B.V, 2017).

[6] H. Opoku, B. Nketia-Yawson, E. S. Shin, and Y. Y. Noh, "Organic field-effect transistors processed by an environmentally friendly non-halogenated solvent blend," *J. Mater. Chem. C*

6, 661–667 (Royal Society of Chemistry, 2018).

[7] S. Yun, A. Marrocchi, L. Vaccaro, and C. Kim, "Effects of solution-shearing process parameters on charge carrier mobility in green solvent-processed organic field-effect transistors," *Synth. Met.* **291**, 117209 (Elsevier B.V., 2022).

[8] S. H. Hong, D. W. Kim, and S. Y. Park, "Aqueous-alcohol-processable indolo[3,2-b]indole-based crystalline small molecules for organic field effect transistors with oligo(ethylene glycol) side chains," *Dye. Pigment.* **211**, 111093 (Elsevier Ltd, 2023).

[9] B. Lim, H. Sun, J. Lee, and Y. Y. Noh, "High performance solution processed organic field effect transistors with novel diketopyrrolopyrrole-containing small molecules," *Sci. Rep.* **7**, 1–8 (Springer US, 2017).

[10] S. Sanda, R. Nakamichi, T. Nagase, T. Kobayashi, K. Takimiya, Y. Sadamitsu, and H. Naito, "Effect of non-chlorinated solvents on the enhancement of field-effect mobility in dioctylbenzothienobenzothiophene-based top-gate organic transistors processed by spin coating," *Org. Electron.* **69**, 181–189 (Elsevier, 2019).

[11] G. Zhang, W. Zhou, M. Kim, M. Sun, H. Lu, L. Qiu, K. Cho, and Y. Ding, "Acceptor-donor-acceptor molecule processed using polar non-halogenated solvents for organic field-effect transistors," *J. Mater. Chem. C* **8**, 6496–6502 (Royal Society of Chemistry, 2020).

[12] Z. B. Henson, P. Zalar, X. Chen, G. C. Welch, T. Q. Nguyen, and G. C. Bazan, "Towards environmentally friendly processing of molecular semiconductors," *J. Mater. Chem. A* **1**, 11117–11120 (2013).

[13] D. Corzo, D. Rosas-Villalva, C. Amruth, G. Tostado-Blázquez, E. B. Alexandre, L. H. Hernandez, J. Han, H. Xu, M. Babics, et al., "High-performing organic electronics using terpene green solvents from renewable feedstocks," *Nat. Energy* **8**, 62–73 (Springer US, 2023).

[14] G. J. N. Wang, F. Molina-Lopez, H. Zhang, J. Xu, H. C. Wu, J. Lopez, L. Shaw, J. Mun, Q. Zhang, et al., "Nonhalogenated Solvent Processable and Printable High-Performance Polymer Semiconductor Enabled by Isomeric Nonconjugated Flexible Linkers" *Macromolecules* **51**, 4976–4985 (2018).

[15] J. R. Matthews, W. Niu, A. Tandia, A. L. Wallace, J. Hu, W. Y. Lee, G. Giri, S. C. B. Mannsfeld, Y. Xie, et al., "Scalable synthesis of fused thiophene-diketopyrrolopyrrole semiconducting polymers processed from nonchlorinated solvents into high performance thin film transistors," *Chem. Mater.* **25**, 782–789 (2013).

[16] B. Fu, C. Y. Wang, B. D. Rose, Y. Jiang, M. Chang, P. H. Chu, Z. Yuan, C. Fuentes-Hernandez, B. Kippelen, et al., "Molecular engineering of nonhalogenated solution-processable bithiazole-based electron-transport polymeric semiconductors," *Chem. Mater.* **27**, 2928–2937 (2015).

[17] K. Takimiya, H. Ebata, K. Sakamoto, T. Izawa, T. Otsubo, and Y. Kunugi, "2,7-Diphenyl[1]benzothieno[3,2-b]benzothiophene, a new organic semiconductor for air-stable organic field-effect transistors with mobilities up to 2.0 cm2 V-1 s-1," *J. Am. Chem. Soc.* **128**, 12604–12605 (2006).

[18] H. Ebata, T. Izawa, E. Miyazaki, K. Takimiya, M. Ikeda, H. Kuwabara, and T. Yui, "Highly soluble [1]benzothieno[3,2-b]benzothiophene (BTBT) derivatives for high-performance, solution-processed organic field-effect transistors," *J. Am. Chem. Soc.* **129**, 15732–15733 (2007).

[19] Y. Yuan, G. Giri, A. L. Ayzner, A. P. Zoombelt, S. C. B. Mannsfeld, J. Chen, D. Nordlund, M. F. Toney, J. Huang, et al., “Ultra-high mobility transparent organic thin film transistors grown by an off-centre spin-coating method,” *Nat. Commun.* **5**, 1–9 (Nature Publishing Group, 2014).

[20] Y. He, M. Sezen, D. Zhang, A. Li, L. Yan, H. Yu, C. He, O. Goto, Y.-L. Loo, et al., “High Performance OTFTs Fabricated Using a Calamitic Liquid Crystalline Material of 2-(4-Dodecyl phenyl)[1]benzothieno[3,2- b][1]benzothiophene,” *Adv. Electron. Mater.* **2**, 1600179 (2016).

[21] K. He, W. Li, H. Tian, J. Zhang, D. Yan, Y. Geng, and F. Wang, “Asymmetric Conjugated Molecules Based on [1]Benzothieno[3,2-b][1]benzothiophene for High-Mobility Organic Thin-Film Transistors: Influence of Alkyl Chain Length,” *ACS Appl. Mater. Interfaces* **9**, 35427–35436 (2017).

[22] S. Guo, Y. He, I. Murtaza, J. Tan, J. Pan, Y. Guo, Y. Zhu, Y. He, and H. Meng, “Alkoxy substituted [1]benzothieno[3,2-b][1]benzothiophene derivative with improved performance in organic thin film transistors,” *Org. Electron.* **56**, 68–75 (2018).

[23] Y. He, W. Xu, I. Murtaza, D. Zhang, C. He, Y. Zhu, and H. Meng, “Molecular phase engineering of organic semiconductors based on a [1]benzothieno[3,2-b][1]benzothiophene core,” *RSC Adv.* **6**, 95149–95155 (Royal Society of Chemistry, 2016).

[24] G. Schweicher, V. Lemaur, C. Niebel, C. Ruzié, Y. Diao, O. Goto, W. Lee, Y. Kim, J. Arlin, et al., “Bulky End-Capped [1]Benzothieno[3,2- b]benzothiophenes: Reaching High-Mobility Organic Semiconductors by Fine Tuning of the Crystalline Solid-State Order,” *Adv. Mater.* **27**, 3066–3072 (2015).

[25] M. R. Reddy, H. Kim, C. Kim, and S. Seo, “2-Thiopene[1]benzothieno[3,2- b]benzothiophene derivatives as solution-processable organic semiconductors for organic thin-film transistors,” *Synth. Met.* **235**, 153–159 (Elsevier, 2018).

[26] G. H. Roche, Y.-T. Tsai, S. Clevers, D. Thuau, F. Castet, Y. H. Geerts, J. J. E. Moreau, G. Wantz, and O. J. Dautel, “The role of H-bonds in the solid state organization of [1]benzothieno[3,2-b][1]benzothiophene (BTBT) structures: bis(hydroxy-hexyl)-BTBT, as a functional derivative offering efficient air stable organic field effect transistors (OFETs),” *J. Mater. Chem. C* **4**, 6742–6749 (2016).

[27] M. Ullah, R. Wawrzinek, R. C. R. Nagiri, S. C. Lo, and E. B. Namdas, “UV–Deep Blue–Visible Light-Emitting Organic Field Effect Transistors with High Charge Carrier Mobilities,” *Adv. Opt. Mater.* **5**, 1–7 (2017).

[28] A. Y. Amin, A. Khassanov, K. Reuter, T. Meyer-Friedrichsen, and M. Halik, “Low-Voltage Organic Field Effect Transistors with a 2-Tridecyl[1]benzothieno[3,2- b][1]benzothiophene Semiconductor Layer,” *J. Am. Chem. Soc.* **134**, 16548–16550 (2012).

[29] H. Iino, T. Usui, and J. I. Hanna, “Liquid crystals for organic thin-film transistors,” *Nat. Commun.* **6**, 1–8 (Nature Publishing Group, 2015).

[30] H. Monobe, L. An, P. Hu, B. Q. Wang, K. Q. Zhao, and Y. Shimizu, “Charge transport property of asymmetric Alkyl-BTBT LC semiconductor possessing a fluorophenyl group,” *Mol. Cryst. Liq. Cryst.* **647**, 119–126 (Taylor & Francis, 2017).

[31] P. Tisovský, A. Gáplovský, K. Gmucová, M. Novota, M. Pavúk, and M. Weis, “Synthesis and characterization of new [1]benzothieno[3,2-b]benzothiophene derivatives with alkyl-thiophene core for application in organic field-effect transistors,” *Org. Electron.* **68**, 121–128 (Elsevier, 2019).

[32] W. Park, C. Yun, S. Yun, J. Lee, S. Bae, D. Ho, T. Earmme, C. Kim, and S. Seo, “Journal of

Industrial and Engineering Chemistry organic semiconductor for solution-processed organic thin film transistors," *J. Ind. Eng. Chem.* **114**, 161–170 (The Korean Society of Industrial and Engineering Chemistry, 2022).

[33] S. Yun, C. Yun, D. Ho, W. Chae, T. Earmme, C. Kim, and S. Seo, "Side chain engineering of [1]benzothieno[3,2-b]benzothiophene (BTBT)-based semiconductors for organic field-effect transistors," *Synth. Met.* **285**, 117022 (Elsevier B.V., 2022).

[34] T. Adrian, S. Hofer, T. Salzillo, C. Ruzié, G. Schweicher, R. Resel, and M. Mas-Torrent, "Mobility anisotropy in the herringbone structure of asymmetric Ph-BTBT-10 in solution sheared thin film transistors †," *J. Mater. Chem. C* **9**, 7186–7193 (2021).

[35] M. Qi, D. Zhang, Y. Zhu, C. Zhao, A. Li, F. Huang, Y. He, and H. Meng, "Anthracene-[1]benzothieno[3,2-b][1]benzothiophene (BTBT) dyad and triads as p-type semiconductors for organic field-effect transistors and phototransistors," *J. Mater. Chem. C* **12**, 6578–6587 (Royal Society of Chemistry, 2024).

[36] S. Inoue, H. Minemawari, J. Tsutsumi, M. Chikamatsu, T. Yamada, S. Horiuchi, M. Tanaka, R. Kumai, M. Yoneya, et al., "Effects of Substituted Alkyl Chain Length on Solution-Processable Layered Organic Semiconductor Crystals," *Chem. Mater.* **27**, 3809–3812 (2015).
